# Maternal Colonization With Group B *Streptococcus* and Serotype Distribution Worldwide: Systematic Review and Meta-analyses

**DOI:** 10.1093/cid/cix658

**Published:** 2017-11-06

**Authors:** Neal J Russell, Anna C Seale, Megan O’Driscoll, Catherine O’Sullivan, Fiorella Bianchi-Jassir, Juan Gonzalez-Guarin, Joy E Lawn, Carol J Baker, Linda Bartlett, Clare Cutland, Michael G Gravett, Paul T Heath, Kirsty Le Doare, Shabir A Madhi, Craig E Rubens, Stephanie Schrag, Ajoke Sobanjo-ter Meulen, Johan Vekemans, Samir K Saha, Margaret Ip, for the GBS Maternal Colonization Investigator Group, Edwin Asturias, Rajid Gaind, Parveen Kumar, Beena Anthony, Lola Madrid, Quique Bassat, Chendi Zhu, Mingjing Luo, Daram Nagarjuna, Subradeep Majumder

**Affiliations:** 1 Maternal, Adolescent, Reproductive and Child Health Centre, London School of Hygiene & Tropical Medicine, United Kingdom;; 2 King’s College London, United Kingdom;; 3 College of Health and Medical Sciences, Haramaya University, Dire Dawa, Ethiopia;; 4 Centre for International Child Health, Imperial College London, United Kingdom;; 5 Paediatric Infectious Diseases Research Group, St George’s, University of London, United Kingdom;; 6 Hospital Clínica Corpas, Bogotá, Colombia;; 7 Departments of Pediatrics and Molecular Virology and Microbiology, Baylor College of Medicine, Houston, Texas;; 8 Department of International Health, Johns Hopkins Bloomberg School of Public Health, Baltimore, Maryland;; 9 Medical Research Council: Respiratory and Meningeal Pathogens Research Unit, and Department of Science and Technology/National Research Foundation: Vaccine Preventable Diseases, Faculty of Health Sciences, University of the Witwatersrand,Johannesburg, South Africa;; 10 Global Alliance to Prevent Prematurity and Stillbirth; 11 Department of Obstetrics and Gynecology, University of Washington, Seattle, Washington;; 12 National Institute for Communicable Diseases, National Health Laboratory Service, Johannesburg, South Africa;; 13 Department of Global Health, University of Washington, Seattle;; 14 National Center for Immunization and Respiratory Diseases, Centers for Disease Control and Prevention, Atlanta, Georgia;; 15 Bill & Melinda Gates Foundation, Seattle, Washington;; 16 World Health Organization, Geneva, Switzerland;; 17 Bangladesh Institute of Child Health, Dhaka;; 18 Department of Microbiology, Faculty of Medicine, Chinese University of Hong Kong

**Keywords:** group B *Streptococcus*, colonization, vaginal, pregnancy, serotypes

## Abstract

**Background:**

Maternal rectovaginal colonization with group B *Streptococcus* (GBS) is the most common pathway for GBS disease in mother, fetus, and newborn. This article, the second in a series estimating the burden of GBS, aims to determine the prevalence and serotype distribution of GBS colonizing pregnant women worldwide.

**Methods:**

We conducted systematic literature reviews (PubMed/Medline, Embase, Latin American and Caribbean Health Sciences Literature [LILACS], World Health Organization Library Information System [WHOLIS], and Scopus), organized Chinese language searches, and sought unpublished data from investigator groups. We applied broad inclusion criteria to maximize data inputs, particularly from low- and middle-income contexts, and then applied new meta-analyses to adjust for studies with less-sensitive sampling and laboratory techniques. We undertook meta-analyses to derive pooled estimates of maternal GBS colonization prevalence at national and regional levels.

**Results:**

The dataset regarding colonization included 390 articles, 85 countries, and a total of 299924 pregnant women. Our adjusted estimate for maternal GBS colonization worldwide was 18% (95% confidence interval [CI], 17%–19%), with regional variation (11%–35%), and lower prevalence in Southern Asia (12.5% [95% CI, 10%–15%]) and Eastern Asia (11% [95% CI, 10%–12%]). Bacterial serotypes I–V account for 98% of identified colonizing GBS isolates worldwide. Serotype III, associated with invasive disease, accounts for 25% (95% CI, 23%–28%), but is less frequent in some South American and Asian countries. Serotypes VI–IX are more common in Asia.

**Conclusions:**

GBS colonizes pregnant women worldwide, but prevalence and serotype distribution vary, even after adjusting for laboratory methods. Lower GBS maternal colonization prevalence, with less serotype III, may help to explain lower GBS disease incidence in regions such as Asia. High prevalence worldwide, and more serotype data, are relevant to prevention efforts.

Group B *Streptococcus* (GBS; *Streptococcus agalactiae*) via maternal rectovaginal colonization, causes a spectrum of disease including maternal infection, stillbirth, and early- and late-onset sepsis in newborns, and may contribute to preterm delivery and hypoxic ischemic encephalopathy [1]. Thus, ascertaining the worldwide prevalence and serotype distribution of GBS colonizing the rectovaginal tracts of pregnant women is critical [2–4].

There may be true differences in GBS maternal colonization prevalence, with variation reported by region [5], ethnicity, and socioeconomic status [6]. However, some of this variation may be due to methodological issues, such as time of GBS screening (during pregnancy or at delivery [7]), sampling site (in particular, whether rectal samples were performed [8–11]), and laboratory culture techniques, notably use of selective enrichment broth [12]. There is no established international standard for sampling for maternal GBS colonization; however, the recommendation by the Centers for Disease Control and Prevention (CDC) [13] of rectovaginal swabs at 35–37 weeks’ gestation with selective enrichment broth is frequently referred to, but not always applied especially in low- and middle-income settings. Reviews that do not take into account these sources of variation may be misleading, especially if the methods differ in certain regions, and may underestimate prevalence when methods are less sensitive, but may exclude large geographical areas if strict criteria are followed.

A recent review, based on studies using the recommended methods described above, estimated maternal GBS prevalence as 17.9% (95% confidence interval [CI], 16.2%–19.7%) worldwide, ranging from 11.1% (95% CI, 6.8%–15.3%) in Southeast Asia to 22.4% in Africa (95% CI, 18.1%–26.7%) [5]. This review included 78 studies from 37 countries, with major gaps in some regions, notably Africa and Asia. By employing broader inclusion criteria, we aimed to capture the largest possible geographical spread of data on prevalence of maternal GBS colonization, while also collecting variables related to specimen collection and processing to adjust for studies where less sensitive methods were used.

In addition to the prevalence of GBS colonization in pregnant women, serotype distribution, which has not previously been systematically reviewed, is also important, both in terms of associations with invasive disease and thus potential vaccine relevance. There are currently 10 GBS serotypes (Ia, Ib, II, III, IV, V, VI, VII, VIII, IX) identified, based on the immunologic reactivity of the GBS capsular polysaccharides [14]. Some serotypes are associated with more virulent clones and thus a propensity to invasive GBS disease [2]. This particularly applies to serotype III, which is frequently associated with the hypervirulent clonal complex (CC) 17, a common cause of late-onset GBS disease [15–21] and, in particular, of meningitis [22]. Two of the 3 maternal vaccines in development are serotype-specific [23, 24] and their coverage will depend on the circulating serotypes.

This paper is the second in an 11-article supplement estimating the burden of group B streptococcal disease in pregnant women, stillbirths, and infants, which is important in terms of public health policy, notably to inform vaccine development [1]. The supplement includes systematic reviews and meta-analyses on adverse outcomes associated with GBS around birth [25–32] to provide input parameters for worldwide estimates [23]. Figure 1 shows the disease schema for GBS, and the important first step of maternal colonization, which is the focus of this article.

**Figure 1.**
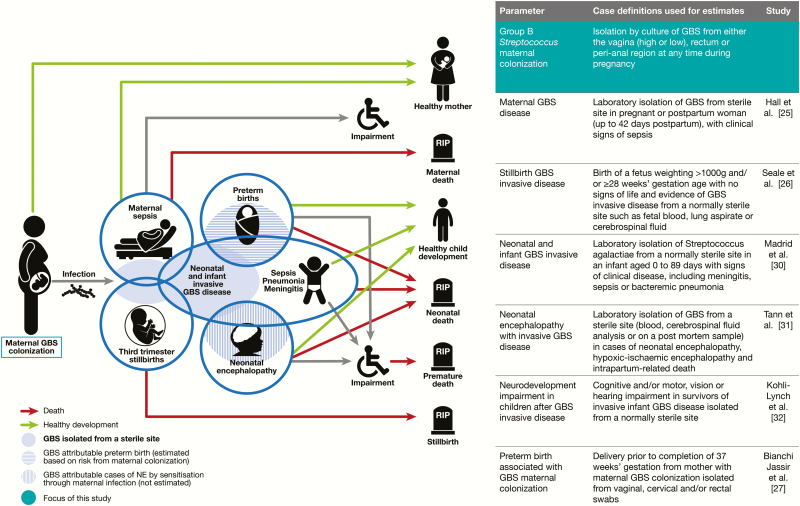
Maternal group B *Streptococcus* (GBS) colonization in GBS disease schema, as described by Lawn et al [1]. Abbreviations: GBS, group B *Streptococcus*; NE, neonatal encephalopathy.

The objectives of this study were to:

1. Undertake comprehensive and systematic literature reviews and meta-analyses of
  a. maternal GBS colonization prevalence for countries, regions, and worldwide; and
  b. serotype distribution of GBS in maternal colonization.
2. Assess the inclusion criteria for data estimating the burden of GBS in pregnancy for women, stillbirth, and infants, with and without additional adjustment for these criteria;
3. Evaluate the data gaps and make recommendations for future research.

## METHODS

This article is part of a study entitled “Systematic estimates of the burden of GBS worldwide in pregnant women, stillbirths and infants.” The protocol was approved by the Clinical Research Ethics Committee (reference number 11966) at the London School of Hygiene & Tropical Medicine and approved on 30 November 2016.

### Definitions

GBS colonization was defined as isolation by culture of GBS from either the vagina (high or low), rectum, or perianal region at any time during pregnancy.

### Search Strategy and Selection Criteria

We identified data by systematic review of the published literature and through development of an investigator group of clinicians, researchers, and relevant professional institutions worldwide. The reviews and meta-analyses are reported according to international guidelines [1, 33, 34].

Our search of published literature, dated up to 30 January 2017, included Medline, Embase, Scopus, Literature in the Health Sciences in Latin America and the Caribbean (LILACS), and the World Health Organization Library Information System (WHOLIS) using search terms relating to mothers, pregnancy, and streptococci, with no language or date restrictions (see Supplementary Table 1 for full search terms). To ensure inclusion of data in languages that may otherwise be missed in these databases, we also searched a Chinese database (http://www.cnki.com.cn/index.htm), with a time restriction of 3 years, and a Russian database (Cyberleninka), with no date restrictions. Abstraction of data from articles in foreign languages was done with translators and only automated if translators were unavailable.

Finally, we searched reference lists of all relevant articles published after 2005, and other publications and reviews [5], focused on regions (Europe [35], Latin America [36], and low-income contexts [37]), as well maternal GBS serotypes [38].

We screened titles and abstracts according to specified inclusion and exclusion criteria, followed by selection of full texts, and abstraction, as detailed below.

### Inclusion and Exclusion Criteria

We included studies where the population and study design were described, reporting prevalence of group B streptococcal colonization in pregnant women, either during pregnancy (at any gestation) or in labor, as well as on the prevalence of the serotypes of colonizing isolates. Studies were included irrespective of sample type (taken from the vagina [high or low] and/or rectum and/or perianal region) and culture technique, as long as laboratory and sampling techniques were described (for subsequent sensitivity and secondary analyses). Although no date restrictions were applied to the initial search, for United Nations (UN)–defined “developed regions” (which were expected to have adequate recent data), data on maternal GBS colonization prevalence and on colonizing serotypes were only included in the analysis if published after the year 2000, unless a particular developed country only had data before this period.

We excluded studies involving nonpregnant women, where results for pregnant women could not be separately extracted. If prevalence estimates were based on <200 women sampled from that country, these were not included in the final estimation process. We also did not derive prevalence estimates from studies in developed regions that focused solely on comparison of laboratory methods. Studies reporting prevalence of GBS colonization using molecular methods only for detection (such as polymerase chain reaction) or GBS bacteriuria alone were also excluded, due to their lack of comparability with conventional methods, limiting cross-country and regional comparison. Data on serotypes were included if they were clearly identified as colonizing pregnant women vaginally or rectally, and were not from invasive disease. Data were included where they described a cohort of women, or pooled laboratory samples, and studies were excluded if they included <10 bacterial isolates.

### Data Abstraction and Analysis

Two researchers (N. R. and M. O.) abstracted data independently into standard Excel abstraction forms with information on sampling and laboratory methodology and relevant study criteria. Differences in abstraction were resolved through discussion with a third researcher (A. S.). Abstracted data included selection of study participants, description of study setting and participants, culture methods, swab site, and timing of swabs (at delivery or at specified gestational ages). These factors allowed an assessment of the potential for bias in each study.

### Maternal Group B *Streptococcus* Colonization Prevalence: Meta-analyses of Reported Data

We undertook meta-analyses using random effects to estimate the prevalence of maternal GBS colonization worldwide and at national, UN subregion, and regional levels, and used the same approach to estimate the prevalence of maternal GBS serotypes from national to regional levels worldwide.

### Sensitivity Analyses to Inform Adjustment for Biases

Sensitivity analyses were performed to assess potential biases in the data and inform adjustments. We examined:

1. Sample site collection comparing vaginal (high and low) sampling, with rectal sampling and rectovaginal sampling.
2. Microbiological methods (specifically, whether selective enrichment was used).
3. Sample timing (before 35 weeks’ gestation, or at delivery).
4. Rural or urban setting.

We calculated adjustment factors for:

1. Sample site: where only the vagina had been sampled (compared to rectovaginal).
2. Microbiological methods: for the addition of selective enrichment, compared to nonselective agar alone, and to conventional selective agars (blood agars with antibiotics, including Columbia colistin–nalidixic acid [CNA] and neomycin–nalidixic acid [NNA]. (Adjustment was not applied for new [higher sensitivity] selective agars of equivalent sensitivity to selective enrichment.)

However, where both sample site and microbiological methods were insensitive (sampling sites of high vagina or cervix, or rectal swab alone, and studies with combinations of low vaginal swabs but no selective enrichment), or adjustment was not possible due to insufficient data, we excluded studies from final estimates of maternal GBS prevalence. Adjustment factors were not calculated for sample timing or rural or urban setting as studies have not shown a consistent relationship between these factors and colonization prevalence [39–52].

### Maternal Group B *Streptococcus* Colonization Prevalence: Meta-analyses With Adjusted Data

We repeated the initial meta-analyses to estimate the prevalence of maternal GBS colonization and serotype distribution worldwide and by region, subregion, and country level using studies including vaginorectal samples with selective enrichment or with selective agar of equivalent sensitivity, and, after adjustment, vaginal-only samples with selective enrichment or selective agar and vaginorectal samples with conventional selective agar only.

### Meta-analyses of Maternal Group B *Streptococcus* Colonizing Serotypes

Data on serotypes were extracted as reported, as numbers of each serotype identified, with a denominator of number of serotyped samples rather than number of women. Individual meta-analyses were performed on the prevalence of each serotype at national, UN subregional, and regional levels, and the outputs of these meta-analyses were transformed into percentages.

## RESULTS

### Study Selection

**Figure 2.**
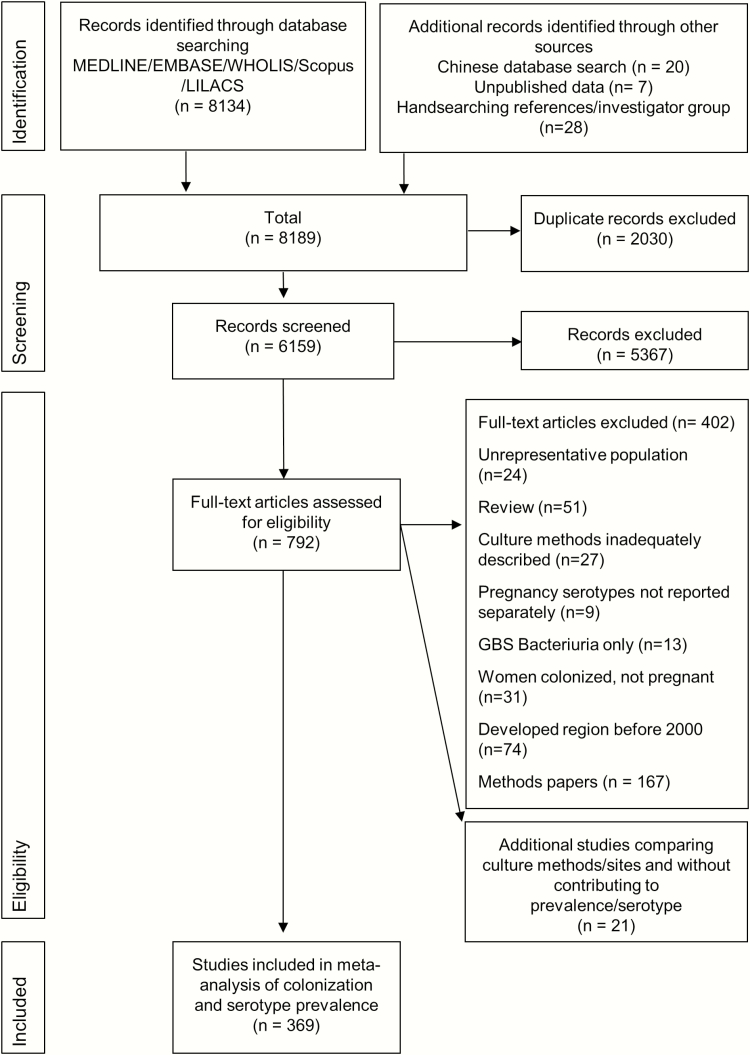
Data search and included studies for maternal group B *Streptococcus* colonization. Abbreviations: LILACS, Latin American and Caribbean Health Sciences Literature; WHOLIS, World Health Organization Library Information System.

We identified 8134 articles, 791 of which were retained after title and abstract screening for review of full texts (Figure 2). An additional 11 articles were identified from the Chinese database and 10 from searching reference lists of the original set of articles. A further 8 unpublished datasets containing anonymized data on 8601 pregnant women were shared by investigators in South Africa, Mozambique, Guatemala, India, and Bangladesh (Supplementary Table 2). The characteristics of the published and unpublished studies are listed in the Supplementary Materials. The majority of studies were in English, although 70 studies were in 17 other languages. The process of selection is detailed in Figure 2. The final analysis included 390 studies (including 412 data points), of which 317 reported maternal GBS colonization prevalence, and 119 reported data on maternal colonizing serotypes (52 included serotype data alone). Forty studies were included in sensitivity analyses to assess sampling site and microbiological methods (21 of which did not otherwise contribute to colonization or serotype data).

### Study Characteristics

This review included data on colonization prevalence from 299924 pregnant women, with serotype data on 16882 maternal samples (16181 of which were typeable by either molecular or conventional methods). Of studies reporting colonization prevalence, 31 (10%) described inclusion of rural participants. Eighty-two (26%) described testing for GBS colonization at delivery, and 94 (30%) described including samples from women tested before 35 weeks’ gestation. Selective culture methods were used in 249 studies (79%), and 215 studies (68%) used rectal as well as vaginal swabs (Supplementary Table 3).

**Figure 3.**
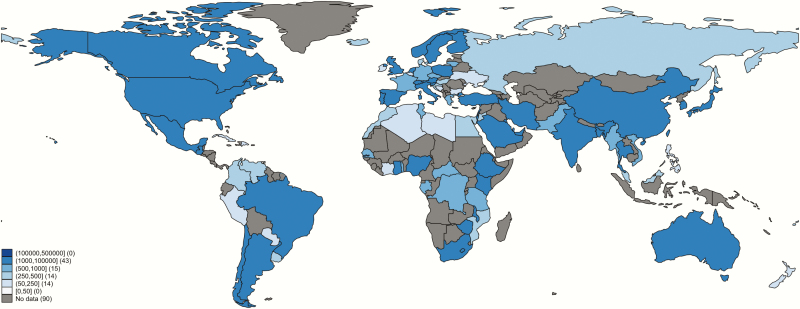
Geographic distribution of included data, showing the range of number of women tested per country. Data for Algeria, Libya, Portugal, and Qatar were excluded from final analyses due to inadequate description of culture methods. Borders of countries/territories in the map do not imply any political statement.

There were 88 studies on colonization prevalence from developed regions (28%), and 229 from low- or middle-income contexts, 45 (19%) of which were published before the year 2000. The geographical distribution of available prevalence data was uneven (Figure 3), with some subregions underrepresented. In particular, there were no data from Central Asia, and data were sparse for Andean Latin America, Oceania, North Africa, and western and central sub-Saharan Africa (Figure 3). Of note, several countries with large populations, such as Russia, had surprisingly few data. A full list of countries included by region and country is in Supplementary Table 4.

**Figure 4.**
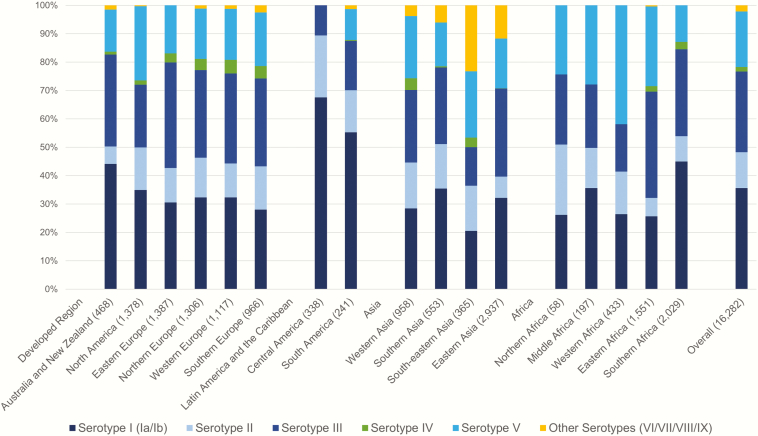
Maternal group B *Streptococcus* colonizing serotype distribution by United Nations subregion.

For maternal colonizing serotypes, the geographical distribution is summarized in Figure 4, and shown in detail in Supplementary Table 5 and Supplementary Figure 1. Developed countries had the largest number of studies, followed by sub-Saharan Africa where a number of large studies have recently been published [53, 54]. Northern Africa had the fewest serotyped isolates (58) of all regions with data. No serotype data were available for central Asia, Melanesia, or the Caribbean. Seven studies (3 of which were from Central America) did not differentiate between serotype Ia and Ib, and therefore a combined serotype I prevalence is reported in Figure 4, with a breakdown into Ia and Ib shown in Supplementary Table 5.

### Maternal Group B *Streptococcus* Colonization Prevalence: Meta-analyses of Reported Data

Including all studies regardless of sample site or microbiological methods and without adjustment, the overall prevalence of maternal GBS colonization worldwide was 15% (95% confidence interval [CI], 14%–16%) (Table 1). Prevalence was highest in the Caribbean (34% [95% CI, 29%–38%]) and lowest in Melanesia (2% [95% CI, 1%–4%]); however, this included data from only 1 study. Europe, North America, and Australia had similar prevalence (95% CI, 15%–21%), with a slightly higher prevalence in Southern Africa (25% [95% CI, 22%–29%]), and seemingly lower prevalence in Western Africa (14%), Central America (10% [95% CI, 7%–14%]), and South, South-Eastern, and Eastern Asia (95% CI, 9%–12%). A list of maternal GBS colonization prevalence by country is presented in Supplementary Table 6.

**Table 1.** Maternal Group B *Streptococcus* Colonization Prevalence Results From Meta-analyses With Reported Data and Meta-analyses With Adjusted Data

Region/ Subregions	No. of Countries	No. of Pregnant Women Tested	Reported Prevalence, %	95% Confidence Interval	Prevalence From Studies With Recommended Methods Only^a^, %	95% Confidence Interval	Adjusted Prevalence^b^, %	95% Confidence Interval
Developed regions	29	144604	18.4	17.0–19.8	21	19.6–22.3	19.2	17.7–20.7
Australia and New Zealand	2	2369	23.3	18.8–27.8	23.3	18.8–27.8	23.3	18.8–27.8
North America	2	27462	22.0	19.2–24.8	23.0	20.9–25.1	23.2	21.1–25.3
Northern Europe	7	6702	20.6	16.6–24.7	24.1	21.9–26.4	22.2	19.1–25.4
Eastern Europe	7	15737	20.8	17.3–24.4	22.9	18.7–27.2	23.0	19.2–26.8
Southern Europe	5	42870	15.4	12.2–18.7	16.7	14.7–18.6	17.6	14.5–20.8
Western Europe	6	49464	15.2	13.1–17.3	18.3	16.0–20.7	19.5	13.9–25.1
Americas	13	20507	18.3	15.8–20.7	19.6	16.7–22.5	20.9	18.1–23.7
South America	8	16141	15.9	13.5–18.2	15.7	13.0–18.5	18.4	15.5–21.3
Central America	2	3229	10.2	6.7–13.8	15.7	13.3–18.0	17.1	13.2–21.0
Caribbean	3	1137	33.5	28.8–38.3	33.5	28.8–38.3	34.7	29.5–39.9
Asia	20	98842	11.0	10.0–12.0	11.6	10.5–12.7	12.8	11.8–13.9
Western Asia	7	15124	14.3	11.-16.6	14.5	11.7–17.4	14.7	12.1–17.4
Central Asia	0	…	…	…	…	…	…	…
Southern Asia	4	15838	10.0	8.3–11.6	10.0	7.5–12.6	12.5	10.2–14.8
South-Eastern Asia	6	4591	12.0	9.3–14.7	14.4	9.5–19.2	14.4	11.5–17.4
Eastern Asia	3	63289	9.2	7.6–10.8	9.1	8.2–10.0	11.1	9.9–12.4
Africa	19	36130	18.2	16.1–20.4	20.7	17.6–23.7	21.3	18.5–24.2
Northern Africa	3	1923	20.0	15.8–24.3	20.5	15.5–25.4	22.9	17.9–28.0
Western Africa	6	4860	13.6	9.0–18.3	17.2	6.2–28.3	17.5	10.8–24.1
Middle Africa	3	2058	18.6	16.9–20.3	19.3	15.9–22.7	23.9	14.7–33.1
Eastern Africa	6	14071	18.2	15.0–21.5	19.4	15.5–23.3	19.4	15.9–23.0
Southern Africa	1	13218	25.3	22.1–28.5	29.5	27.4–31.5	28.9	26.6–31.2
Oceania	1	440	19.0	6.8–31.3	…	…	…	…
Melanesia	1	440	2.0	0.6–3.5	…	…	…	…
Overall		300176	15.2	14.3–16.0	17.4	16.3–18.5	18.0	16.9–19.1

^a^Recommended methods refers to studies including both rectal (or perianal) and vaginal swabs, and with selective enrichment or a selective agar proven to provide equivalent sensitivity.

^b^Adjusted prevalence for sample site and microbiological methods.

### Sensitivity Analyses to Inform Adjustment for Biases

Sensitivity analyses were performed on:

1. Sample site collection: studies using CDC-recommended sampling with rectovaginal swabs and selective enrichment (or selective agar of equivalent sensitivity):

Including only studies using CDC-recommended methods, we found 188 studies with a maternal GBS colonization prevalence of 17% (95% CI, 16%–19%), higher than the initial analysis with all the included studies. The prevalence for subregions and countries also changed because of geographic tendencies to use different methods (see Table 1 and Supplementary Materials, respectively). Some regions with low prevalence on crude analysis were excluded from this analysis, but some in some regions such as some Asian countries, the low prevalence persisted.

2. Sample timing (before and after 35 weeks’ gestation, or at delivery):

The overall prevalence of maternal GBS colonization in studies that reported samples from pregnant women before 35 weeks’ gestation was 17% (95% CI, 15%–18%), then 15% (95% CI, 13%–16%) in those sampled after 35 weeks, and 14% (95% CI, 13%–16%) at delivery.

3. Rural or urban settings:

In mixed urban/rural settings, the prevalence of maternal GBS colonization was 20% (95% CI, 17%–23%) (24 studies from 14 subregions), and 21% (95% CI, 15%–27%) in exclusively rural settings (6 studies) (Supplementary Figures 2 and 3).

### Adjustments to Address Biases

We calculated adjustment factors for sample site and microbiological methods (Table 2):

**Table 2.** Adjustment Factors to Address Biases

Addition or Inclusion	Comparison Method (of Lower Sensitivity)	CDC-Recommended Method	No. of Studies	Adjustment Factor (Factor Increase in Sensitivity)	(95% CI)
Addition of rectal swabs to vaginal swabs (vaginal vs vaginorectal sampling)	Vaginal only	Rectovaginal	27	1.4	(1.3–1.6)
Inclusion of selective enrichment broth to unselective agar	Blood agar alone without antibiotics	Agar + selective enrichment broth - Todd Hewitt + gentamicin and nalidixic acid - Todd-Hewitt + colistin and nalidixic acid	13	1.9	(1.6–2.1)
Inclusion of selective enrichment broth to a blood agar including antibiotics	Blood agar with antibiotics - Columbia colistin–nalidixic acid - Neomycin–nalidixic acid	Agar + selective enrichment broth - Todd Hewitt + gentamicin and nalidixic acid - Todd-Hewitt + colistin and nalidixic acid	10	1.5	(1.3–1.7)

Most common examples are shown. For more details and meta-analyses, see the Supplementary Materials.

Abbreviations: CDC, Centers for Disease Control and Prevention; CI, confidence interval.

• For sample site: comparing sampling vaginorectally vs vaginally only, based on 27 studies, the increase in detection (risk ratio) was 1.4 (95% CI, 1.3–1.6) (Supplementary Table 7 and Supplementary Figure 4).
• For microbiological methods: comparing a conventional selective agar (blood agar with antibiotics: CNS [most commonly] or NNA) with and without enrichment (10 studies), the increase in detection (risk ratio) was 1.5 (95% CI, 1.3–1.7) (Supplementary Table 8 and Supplementary Figure 5). Compared to an unselective agar (eg, sheep blood agar alone) with and without selective enrichment (13 studies), the relative increase in sensitivity with selective enrichment was 1.9 (95% CI, 1.6–2.1) (Supplementary Table 9 and Figure 6).

### Maternal Group B *Streptococcus* Colonization Prevalence: Meta-analyses With Adjusted Data

**Figure 5.**
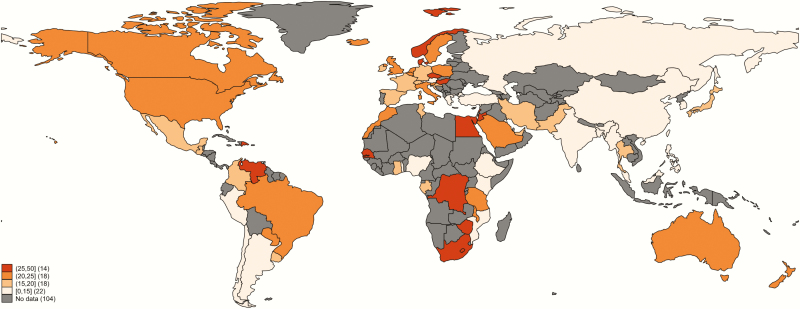
Prevalence of group B *Streptococcus* (GBS) colonization by country, adjusting for sampling site and laboratory culture method. Borders of countries/territories in map do not imply any political statement.

The overall prevalence of maternal GBS was 18% (95% CI, 17%–19%) (Table 1 and Supplementary Figure 7). The adjusted prevalence of GBS colonization by country is shown in Figure 5 (detailed in Supplementary Table 6). The Caribbean had the highest prevalence of colonization (35% [95% CI, 35%–40%]), and Southern Asia and Eastern Asia had the lowest prevalence of GBS colonization (13% and 11%, respectively) (Supplementary Figures 8–11). Within these subregions, the Republic of Korea (8% [95% CI, 7%–9%]), Myanmar (9% [95% CI, 7%–11%]), India (10% [95% CI, 7%–12%]), Bangladesh (11% [95% CI, 4%–18%]), and China (11% [95% CI, 10%–13%]) had the lowest prevalence, with higher prevalence found in Iran (16% [95% CI, 12%–20%]), Japan (16% [95% CI, 12%–20%]), and Pakistan (20% [95% CI, 6%–34%]). Importantly, some the data in some countries and regions could not be adjusted for (eg, Fiji, Melanesia) due to inadequate methods in the studies.

### Meta-analyses of Maternal Group B *Streptococcus* Colonizing Serotypes

Serotypes Ia, Ib, II, III, and V colonized the rectovaginal tracts of women in all regions, accounting for 98% of serotypes globally; however, variation existed in the reported prevalence of these serotypes and, perhaps most importantly, in the prevalence of serotype III. Compared to an overall serotype III prevalence of 25%, Central America (11% of colonized women [95% CI, 7%–14%]) and South-Eastern Asia (12% [95% CI, 6%–18%]), as well as some South Asian countries including India (11% [95% CI, 0–23%]) and Bangladesh (11% [95% CI, 7%–15%]), had a lower reported prevalence of serotype III (Figure 4). In particular, if the region of South Asia is separated from Iran (included in the UN Southern Asia subregion), then it has a particularly low prevalence of serotype III (10.4%). Other regional differences included greater serotype V prevalence (along with lower serotype III prevalence) in Western Africa. Other serotypes (VI, VII, VIII, and IX) appear to be much more frequently reported in Southern, South-Eastern, and Eastern Asia (Supplementary Tables 10 and 11; Supplementary Figures 12–16). Together they account for 20% of serotypes in South-Eastern Asia, for example.

## DISCUSSION

GBS colonizes pregnant women in all regions of the world in which studies have been conducted. The prevalence rates vary in different geographical regions, and a strength of our review is that we sought to account as much as possible for variation due to differences in sampling and methodology, to shed light on true epidemiological variation. The worldwide prevalence postadjustment was estimated at 18% (95% CI, 17%–19%) whereas prevalence preadjustment was 15% (95% CI, 14%–16%). Some regions had very different prevalence estimates after adjustment, which demonstrates how prevalence may have been underestimated previously. The data in some countries were inadequate and could not be adjusted for, and their crude prevalences are likely to be significant underestimates of true prevalence. However, considerable regional variation remained; in particular, Southern and Eastern Asian countries had a lower estimated prevalence of maternal GBS colonization. In addition, there were clear regional differences in colonizing serotypes. Notably, serotype III was less frequently found in Asia, with otherwise less common serotypes such as VI, VII, and VIII more frequently found. Within Africa, serotype V is more frequently reported in Western Africa than in other regions.

Differences in prevalence of GBS colonization and serotype distribution among mothers in different regions may help to explain apparent differences in incidence of newborn invasive GBS disease. Low apparent incidence of neonatal early-onset GBS disease in South Asia might, for example, be partly explained by a combination of lower overall prevalence of maternal GBS colonization and a lower prevalence of serotype III in those who are colonized. However, we need more data, particularly with sensitive methods, on maternal GBS colonization prevalence and serotypes, particularly from the countries where there were limited or no data, and where colonization prevalence was very different to that found elsewhere (eg, Southern Asia, Melanesia, Central America, and Central Asia).

This is the largest systematic review and meta-analysis to date of published and unpublished data on maternal GBS colonization and serotype distribution globally and involved 299924 pregnant women, with pooled estimates of maternal GBS colonization prevalence made for 82 countries, in comparison with 73791 women and 37 countries included in the most recent previous review [5]. This is also the first global systematic review of serotypes colonizing pregnant women, including 16181 bacterial isolates. However, there are limitations. The majority of studies with the most sensitive sampling and microbiological techniques and the largest sample sizes came from high-income contexts. With the exception of a few recent reports [53, 54], studies from low-income contexts have frequently used less-sensitive sampling and microbiological methods, and have had small sample sizes and overrepresentation of urban referral hospitals. For many low-income contexts in particular, the data are thus potentially biased toward urban settings. Few studies directly compared urban and rural prevalence of GBS colonization, and these have shown conflicting results [49, 53, 55], as indeed have studies comparing primary and tertiary care [56] and high and low socioeconomic status [6, 53, 57–59]. Therefore, there may be variation, in different local contexts, in the extent and direction in which these factors influence maternal GBS colonization prevalence. However, the reported variation may also be due to selection biases, especially for varying levels of care. In this review, although there were few direct comparisons, the overall maternal GBS colonization prevalence in rural contexts was comparable to urban contexts.

Other limitations include differences across studies in the timing of swabs. Screening later in pregnancy is more predictive of GBS colonization during labor and therefore of the risk of neonatal invasive disease [44, 51, 60]. This review demonstrated a marginally higher prevalence in studies with sampling before 35 weeks (16.5% [95% CI, 14.9%–18.0%] vs 15.1% [95% CI, 13.8%–16.4%] after 35 weeks) which is supported by some longitudinal studies showing modest downward trends in prevalence during pregnancy, but contradicted by others [39–52, 61–64]. Current evidence suggests that overall population prevalence is relatively stable during pregnancy even if fluctuant at an individual level and that, for the purposes of population-level estimates of colonization, sampling pregnant women in the second trimester or third is unlikely to bias an overall estimate, even if swabs early in pregnancy are poor predictors of colonization at delivery.

We addressed some of the limitations in the data through adjustment where less-sensitive sampling or microbiological methods had been used and allowed inclusion of data from more low-income contexts. This assumed a consistent difference in sensitivity, which may not hold for all populations. A single recent study in South Africa found that selective enrichment had lower sensitivity when used on rectal samples compared to direct plating onto selective agars [65], although the order of plating may have contributed to this. Overall, however, from our analyses (Supplementary Figures 1–3), the increase in sensitivity when the most sensitive methods were used was consistent, and adjustment factors were tightly defined within 95% confidence intervals. Other factors that could affect the sensitivity of methods in different settings could not be accounted for, such as use of blood agar without specifying the source from which the blood was derived, which would lead to lower sensitivity if human blood, with or without ethylenediaminetetraacetic acid, were used instead of sheep blood, for example.

Our comprehensive review of GBS maternal colonization and serotype distribution highlights the important gaps in data that still exist. Future research on maternal GBS colonization should prioritize high-quality data from low-income contexts, especially rural populations and regions where there are large data gaps, such as South and Central Asia, Central and Western Africa, and Oceania. More phylogenetic data, including sequence type clonal complex and serotype distributions, are also needed to understand the emergence and relationship between colonization and disease.

Despite data gaps, it is clear that GBS is present in all regions of the world as a pathogen colonizing pregnant women, and this finding has important implications for public health policy. The myths that GBS is only a pathogen in high-income contexts are no longer tenable. The associated burden would be amenable to prevention by intrapartum antibiotic prophylaxis or maternal immunization. Improved data, including on serotypes, are important to guide effective decision making, and also monitor the impact of intervention (Table 3).

**Table 3.** Key Findings and Implications

What’s new about this? • This dataset covers 85 countries and includes 299924 pregnant women, more than doubling the size of previous reviews, benefiting from translating 70 articles from 17 languages, and accessing unpublished data. In addition, we have undertaken meta-analyses showing consistently higher capture of GBS when sampling is rectovaginal (1.4 [95% CI, 1.3–1.6]) compared to vaginal only, or when selective enrichment is practiced (1.5 [95% CI, 1.3–1.7]). These findings allowed us to adjust input data, increasing comparability.
What was the main finding? • We found a worldwide pooled estimate of 18% (95% CI, 17%–19%) for maternal GBS colonization prevalence, but with regional variation in prevalence (95% CI, 11%–35%), and also for serotype distribution.
How can the data be improved? • Data gaps persist, as while 85 countries had useable data, more than half of 195 UN member states do not. Comparability would be improved by more standard sampling (rectovaginal swabs), laboratory methods (broth enrichment), and even newer more sensitive methods, with more reporting of serotypes and MLST types.
What does it mean for policy and programs? • Our findings suggest that GBS is a common worldwide colonizer of pregnant women and that a GBS vaccine could be valuable in reducing the burden of GBS disease not just in high-income contexts.

Abbreviations: CI, confidence interval; GBS, group B *Streptococcus*; MLST, multilocus sequence typing; UN, United Nations.

## Supplementary Data

Supplementary materials are available at *Clinical Infectious Diseases* online. Consisting of data provided by the authors to benefit the reader, the posted materials are not copyedited and are the sole responsibility of the authors, so questions or comments should be addressed to the corresponding author.

## Supplementary Material

Supplement_Material

## Notes

***Author contributions.*** The concept of the estimates and the technical oversight of the series was led by J. E. L. and A. C. S. The reviews, analyses, and first draft of the paper were undertaken by N. R. with A. C. S., S. K. S., and M. I. Other specific contributions were made by M. O., C. O. S., F. B. J., and J. G. G. The GBS Estimates Expert Advisory Group (C. J. B., L. B., C. C., M. G. G., P. T. H., K. L. D., S. A. M., C. E. R., S. S., A. S.-t. M., J. V.) contributed to the conceptual process throughout, notably on the disease schema and data inputs. The GBS Maternal Colonization Investigator Group (see above) input data for the analyses, and C. Z. and M. L. helped with the search in the Chinese database. All the authors reviewed and gave input to the manuscript.

***Acknowledgments.*** The authors thank Ipek Gurol, Laura Ferreras, Monika Ogorek, Jana Zitha, Kazuyo Machiyama, Ketevan Glonti, Tapan Bhattacharyya, Lola Madrid, Fiorella Bianchi-Jassir, Vladimir Gordeev, Debora Pedrazzoli, and Ludovica Ghilardi for translation of papers in different languages; Francesca Cavallaro for statistical and analytical support, and Jane Falconer for technical support on initial searches; Claudia da Silva for administrative assistance; and Alegria Perez for coordinating author signatures.

***GBS Maternal Colonization Investigator Group.*** Edwin Asturias (Children’s Hospital Colorado), Rajid Gaind (Vardhman Mahavir Medical College and Safdarjung Hospital, New Delhi, India), Parveen Kumar (Division of Neonatology, Department of Pediatrics, Post Graduate Institute of Medical Education & Research, Chandigarh, India), Beena Anthony, Lola Madrid (ISGlobal, Barcelona Institute for Global Health), Quique Bassat (ISGlobal, Barcelona Institute for Global Health, and ICREA, Barcelona, Spain), Chendi Zhu (Department of Microbiology, Faculty of Medicine, Chinese University of Hong Kong), Mingjing Luo (Department of Microbiology, Faculty of Medicine, Chinese University of Hong Kong), Daram Nagarjuna (Dr B. R. Ambedkar Center for Biomedical Research, Delhi India), and Subradeep Majumder (Vardhman Mahavir Medical College and Safdarjung Hospital, New Delhi, India).

***Financial support.*** This supplement was supported by a grant to the London School of Hygiene & Tropical Medicine from the Bill & Melinda Gates Foundation (Grant ID: OPP1131158).

***Supplement sponsorship.*** This article appears as part of the supplement “The Burden of Group B *Streptococcus* Worldwide for Pregnant Women, Stillbirths, and Children,” sponsored by the Bill & Melinda Gates Foundation and coordinated by the London School of Hygiene & Tropical Medicine.

***Potential conflicts of interest.*** Many contributors to this supplement have received funding for their research from foundations, especially the Bill & Melinda Gates Foundation, and several from Wellcome Trust, Medical Research Council UK, the Thrasher Foundation, the Meningitis Research Foundation, and one individual from the US National Institutes of Health. Members of the Expert Advisory Group received reimbursement for travel expenses to attend working meetings related to this series. A. S.-t. M. works for the Bill & Melinda Gates Foundation. C. J. B. has served as a member of the Presidential Advisory Committee for Seqirus Inc and of the CureVac Inc Scientific Advisory Committee, as well as undertaken consultancy work for Pfizer Inc. C. C. has received institutional compensation from Novartis for conducting GBS studies. P. T. H. has been a consultant to Novartis and Pfizer on GBS vaccines but received no funding for these activities. M. I. has undertaken sponsored research from Pfizer on pneumococcal disease in adults and from Belpharma Eumedica (Belgium) on temocillin antimicrobial susceptibility in Enterobacteriaceae. K. L. D. has received funding by the Bill & Melinda Gates Foundation to work on research on GBS serocorrelates of protection to inform vaccine trials, and travel expenses from Pfizer to attend a meeting on an investigator-led project on GBS. S. A. M. has collaborated on GBS grants funded by GlaxoSmithKline and by Pfizer and received personal fees for being member of its advisory committee; he has also collaborated on a GBS grant funded by Minervax. All other authors report no potential conflicts of interest. All authors have submitted the ICMJE Form for Disclosure of Potential Conflicts of Interest. Conflicts that the editors consider relevant to the content of the manuscript have been disclosed.
