# Supplementary material for: Maternal Colonization With Group B Streptococcus and Serotype Distribution Worldwide: Systematic Review and Meta-analyses
Source: Clin Infect Dis. 2017 Nov 6;65(Suppl 2):S100–11. doi: 10.1093/cid/cix658 (PMC5848259; doi:10.1093/cid/cix658)
Supplement: Supplement_Material [file cix658_suppl_supplement_material.pdf]

The burden of Group B *Streptococcus* for pregnant women, stillbirths and children

Paper 2: Maternal colonization with Group B *Streptococcus* and serotype distribution worldwide: systematic review and meta-analyses

Supplementary information

Contents

The burden of Group B *Streptococcus* for pregnant women, stillbirths and children 1

Paper 2: Maternal colonization with Group B *Streptococcus* and serotype distribution worldwide: systematic review and meta-analyses 1

Supplementary information 1

Supplementary Table S1: Search terms 4

Supplementary Table S2: Maternal GBS colonization: study characteristics of unpublished data 5

Supplementary Table S3: Maternal GBS colonization: study characteristics by analysis type 6

Supplementary Table S4: Study Characteristics: Maternal GBS Colonization in pregnancy 7

Supplementary Table S5: Study Characteristics: Maternal GBS Colonization in pregnancy with serotype data. 19

Supplementary Table S6: Maternal GBS colonization prevalence, by country 24

Supplementary Table S7: Maternal GBS colonization prevalence: comparison of sample site. 28

Supplementary Table S8: Maternal GBS Colonization prevalence: studies included in comparison of culture methods 30

Supplementary Table S9: Maternal GBS colonization prevalence: studies included in comparison of culture methods 31

Supplementary Table S10: Maternal GBS serotype distribution by UN sub-region 32

Supplementary Table S11: Maternal GBS serotype distribution by country 33

Supplementary Figure S1: Global distribution of maternal GBS colonization serotype data 35

Supplementary Figure S2: GBS Colonization prevalence: Setting described as rural 36

Supplementary Figure S3: GBS Colonization prevalence: setting described as mixed rural/urban 37

Supplementary Figure S4: Risk ratio for comparison between sampling site (inclusion of rectal swab sample) 38

Supplementary Figure S5: Risk ratio for selective enrichment compared to selective conventional agar* alone without enrichment 39

Supplementary Figure S6: Risk ratio for detection with selective enrichment compared to unselective agar alone 40

Supplementary Figure S7: Maternal GBS prevalence by sub-region 41

Supplementary Figure S8: Maternal GBS prevalence in developed region: adjusted 42

Supplementary Figure S9: Maternal GBS prevalence in Africa: adjusted 43

Supplementary Figure S10: Maternal GBS prevalence in Latin America and the Caribbean: adjusted ` 44

Supplementary Figure S11: Maternal GBS prevalence in Asia: adjusted` 45

Supplementary Figure S12: Maternal GBS colonization serotypes distribution worldwide 46
Supplementary Figure S13: GBS maternal colonization serotypes distribution in developed regions 47
Supplementary Figure S14: GBS maternal colonization serotypes distribution in Africa .. 48
Supplementary Figure S15: GBS maternal colonization serotypes distribution in South and Central America... 49
Supplementary Figure S16: GBS maternal colonization serotypes distribution in Asia 50
References.. 51

Supplementary Table S1: Search terms

**Matern* OR pregnan* OR Antenatal OR Antepartum OR Vagin* OR Recto-Vagin* OR vagino-rectal OR rectovaginal OR Obstetric*OR Pregnancy {MeSH} OR Vagina {MeSH} AND Streptococcus agalactiae OR group b streptococc* OR Streptococc* group B OR Streptococcus agalactiae {MeSH} AND Epidemiolog* OR Prevalence OR Colonis* OR Coloniz* OR Frequenc* OR Screen* OR Carriage OR Serotyp* OR Rate OR Epidemiology {MeSH} OR Prevalence {MeSH} OR Screening {MeSH}**
AND
Streptococcus
Streptococcal
Streptococci AND (Group AND B) or Agalactiae
Streptococcus Agalactiae MeSH Terms

Supplementary Table S2: Maternal GBS colonization: study characteristics of unpublished data

Country	Investigator	Years	Colonization	Serotypes	Number of women tested
**South Africa**	Cutland	2014-2016	Y	Y	4649
**Mozambique**	Madrid	2014-2015	Y	Y	320
**Morocco**	Bassat	2013	Y	N	347
**Guatemala**	Asturius		Y	N	990
**India**	Gaind	2015-2016	Y	N	400
**India**	Kumar	2003-2006	Y	Y	657
**Bangladesh**	Saha	2012-2013	Y	Y	1166
**India**	Anthony		Y	N	72
**Overall**					8601

Supplementary Table S3: Maternal GBS colonization: study characteristics by analysis type

**Study Characteristics**	**Number of studies**
Colonization prevalence	317
Serotype distribution	119
Selective enrichment / High sensitivity selective agar	249
Samples included rectal site	215
Timing of swab described as before 35 weeks	94
Timing of swab described as at delivery	82
Published before year 2000	45
Described inclusion of a Rural population	31
Contributed to adjustment factor analysis	39
Unpublished data sets	8
Total	390

Supplementary Table S4: Study Characteristics: Maternal GBS Colonization in pregnancy

Region	Country	Author	Year	Gestational age when swab taken	Sample Site for Swab	Selective enrichment / agar*	Women tested (n)	GBS Colonized (n)	Prevalence (%)
**Australia and New**	Australia	Gilbert[1]	2002	throughout	Recto/perianal and vaginal	Y	1096	296	27
**Australia and New**	Australia	Hiller[2]	2005	32/36/labor	Recto/perianal and vaginal	Y	865	168	19
**Australia and New**	Australia	Taylor[3]	2006	throughout pregnancy	Recto/perianal and vaginal	Y	168	43	26
**Australia and New Zealand**	New Zealand	Grimwood[4]	2002	35-37	Recto/perianal and vaginal	Y	240	52	22
**Caribbean**	Dominica	Fernandez[5]	2006	delivery	Recto/perianal and vaginal	Y	207	90	43
**Caribbean**	Trinidad	Orrett[6]	2004	3rd trimester	Recto/perianal and vaginal	Y	405	130	32
**Caribbean**	Trinidad	Orrett[7]	2003	3rd trimester	Recto/perianal and vaginal	Y	201	66	33
**Caribbean**	Trinidad	Orrett[8]	1994	3rd trimester	Recto/perianal and vaginal	Y	204	64	31
**Caribbean**	Cuba	Cruz[9]	2014	35-37	Recto/perianal and vaginal	Y	120	33	28
**Central America**	Mexico	Gonzalez[10]	2002	3rd trim	Recto/perianal and vaginal	N	691	97	14
**Central America**	Mexico	Gonzalez[11]	2004		Recto/perianal and vaginal	N	98	8	8
**Central America**	Mexico	Solorzano-Santos[12]	1989	throughout pregnancy	Vaginal	Y	340	35	10
**Central America**	Mexico	Lourdes-Collado[13]	1981	38	Cervical/rectal	Y	200	8	4
**Central America**	Mexico	Ocampo-Torres[14]	2000	labor	Recto/perianal and vaginal	N	910	78	9
**Central America**	Guatema	Asturius	2016	>35 weeks	Recto/perianal and vaginal	Y	990	155	16
**Eastern Africa**	Zimbabw	Mavenyengwa[1	2010	20/26wk/delivery	Recto/perianal and vaginal	Y	672	142	21
**Eastern Africa**	Zimbabw	Mavenyengwa[1	2006	20-30	Recto/perianal and vaginal	Y	300	138	46
**Eastern Africa**	Zimbabw	Mavenyengwa[1	2006	20-30	Recto/perianal and vaginal	Y	100	60	60
**Eastern Africa**	Zimbabw	Whitney[17]	2004	20-32	Vaginal	Y	210	25	12
**Eastern Africa**	Zimbabw	Moyo[18]	2000	throughout	Recto/perianal and vaginal	Y	206	65	32

**Eastern Africa**	Zimbabw	Mason[19]	1996	?	Vaginal	N	399	86	22
**Eastern Africa**	Zimbabw	Mason[20]	1989	labor	Cervical/urethral	N	81	16	20
**Eastern Africa**	Malawi	Gray[21]	2011	3rd trimester	Recto/perianal and vaginal	Y	1857	390	21
**Eastern Africa**	Malawi	Dzowela[22]	2005	>34wks	Recto/perianal and vaginal	N	97	16	16
**Eastern Africa**	Kenya	Seale[23]	2016	delivery	Recto/perianal and vaginal	Y	526	47	9
**Eastern Africa**	Kenya	Seale[23]	2016	delivery	Recto/perianal and vaginal	Y	5470	608	11
**Eastern Africa**	Kenya	Seale[23]	2016	delivery	Recto/perianal and vaginal	Y	1971	279	14
**Eastern Africa**	Tanzania	Ernest[24]	2015	28-42wks	Recto/perianal and vaginal	N	295	28	9
**Eastern Africa**	Tanzania	Joachim[25]	2009	>37	Recto/perianal and vaginal	Y	300	69	23
**Eastern Africa**	Ethiopia	Woldu[26]	2014	35-37	Recto/perianal and vaginal	Y	300	22	7
**Eastern Africa**	Ethiopia	Alemseged[27]	2015	3rd trim	Vaginal	Y	139	19	14
**Eastern Africa**	Ethiopia	Mohammed[28]	2012	35-37	Recto/perianal and vaginal	Y	139	29	21
**Eastern Africa**	Ethiopia	Gebremeskel[29	2015	35-37	Recto/perianal and vaginal	Y	150	17	11
**Eastern Africa**	Ethiopia	Mengist[30]	2016	35-37weeks	Recto/perianal and vaginal	Y	126	24	19
**Eastern Africa**	Mozambi que	De Steenwinkel [31]	2008	35-37	Recto/perianal and vaginal	Y	113	2	2
**Eastern Africa**	Mozambi	Madrid	2016	34-37wks (200),	Recto/perianal and vaginal	Y	320	68	21
**Eastern Africa**	Ethiopia	Woldu[26]	2014	35-37	Recto/perianal and vaginal	Y	300	22	7
**Eastern Asia**	China	Yim[32]	1995	16-24weeks	Vaginal	N	367	4	1
**Eastern Asia**	China	Tsui[33]	2009	booking	Recto/perianal and vaginal	Y	1002	104	10
**Eastern Asia**	China	Wang[34]	2015	35-37	Recto/perianal and vaginal	N	863	56	6
**Eastern Asia**	China	Lu[35]	2014	35-37	Recto/perianal and vaginal	N	2850	201	7
**Eastern Asia**	China	Yanmin Ma[36]	2000	3 periods <20/20-	Vaginal	Y	1039	115	11
**Eastern Asia**	China	Zhang[37]	1995	delivery	Vaginal	N	600	48	8
**Eastern Asia**	China	Liang[38]	1986	>37wks	Cervical/rectal	Y	168	32	19
**Eastern Asia**	China	bsq[39]	2015	36-38 weeks	Recto/perianal and vaginal	Y	350	32	9
**Eastern Asia**	China	gxm[40]	2015	34-37 weeks	Recto/perianal and vaginal	N	1394	52	4
**Eastern Asia**	China	xax[41]	2015	35-37	Vaginal	N	600	52	9
**Eastern Asia**	China	wcl[42]	2015	35-37weeks	Recto/perianal and vaginal	Y	1282	106	8
**Eastern Asia**	China	zqn[43]	2015	36-39	Vaginal	Y	460	38	8
**Eastern Asia**	China	hr[44]	2015	35-37 weeks	Vaginal	Y	1305	157	12

**Eastern Asia**	China	zlh[45]	2015	35-37 weeks	Recto/perianal and vaginal	Y	10141	923	9
**Eastern Asia**	China	wj[46]	2015	35-42	Recto/perianal and vaginal	N	484	31	6
**Eastern Asia**	China	xdy[47]	2015	36-38 weeks	Recto/perianal and vaginal	Y	268	42	16
**Eastern Asia**	China	cy[48]	2015	35-37weeks	Recto/perianal and vaginal	Y	719	70	10
**Eastern Asia**	China	tlj[49]	2013	35-37	Recto/perianal and vaginal	Y	1580	110	7
**Eastern Asia**	China	whj[50]	2013	34-38	Recto/perianal and vaginal	Y	426	81	19
**Eastern Asia**	China	ch[51]	2013	35-37	Recto/perianal and vaginal	Y	500	72	14
**Eastern Asia**	China	sdh[52]	2013	35-38	Recto/perianal and vaginal	N	221	21	10
**Eastern Asia**	China	zy[53]	2013	36-38	Recto/perianal and vaginal	Y	300	25	8
**Eastern Asia**	China	wx[54]	2013	34-36	Rectal only	N	2776	60	2
**Eastern Asia**	China	hyj[55]	2013	35-37	Recto/perianal and vaginal	N	445	17	4
**Eastern Asia**	China	hgc[56]	2013	35-37	Recto/perianal and vaginal	Y	652	49	8
**Eastern Asia**	China	Xie[57]	2016		Vaginal	Y	200	20	10
**Eastern Asia**	China	Yan[58]	2016	?	Recto/perianal and vaginal	Y	398	21	5
**Eastern Asia**	China	Yang[59]	2014	>37wks	Vaginal	N	354	22	6
**Eastern Asia**	China	Li[60]	2016	35-37	Recto/perianal and vaginal	N	12200	2529	21
**Eastern Asia**	China	Fu[61]	2004	>35weeks	Recto/perianal and vaginal	Y	374	56	15
**Eastern Asia**	S.Korea	Kim[62]	2012	35-37	Recto/perianal and vaginal	Y	1845	169	9
**Eastern Asia**	S.Korea	Lee[63]	2010	35-37	Recto/perianal and vaginal	Y	2624	211	8
**Eastern Asia**	S.Korea	Hong[64]	2010	35-37	Recto/perianal and vaginal	Y	1205	121	10
**Eastern Asia**	S.Korea	Uh[65]	1997	delivery	Recto/perianal and vaginal	Y	459	27	6
**Eastern Asia**	S.Korea	Yook[66]	2013	35-37 weeks	Recto/perianal and vaginal	Y	5095	410	8
**Eastern Asia**	S.Korea	Kim[67]	2015	>20 weeks	Recto/perianal and vaginal	Y	107	8	7
**Eastern Asia**	Japan	Terakubo[68]	2002		Vaginal	Y	1404	187	13
**Eastern Asia**	Japan	Matsubara[69]	2002	28wks	Vaginal	Y	583	48	8
**Eastern Asia**	Japan	Kubota[70]	2002	22-36	Vaginal	N	4025	408	10
**Eastern Asia**	Japan	Morozumi[71]	2015	36-39wks	Vaginal	Y	1226	154	13
**Eastern Europe**	Hungary	Losonczi[72]	2002	36-37 weeks	Recto/perianal and vaginal	N	245	67	27
**Eastern Europe**	Hungary	Abrok[73]	2015	pregnancy	Vaginal	Y	100	27	27
**Eastern Europe**	Ukraine	Perebendyuk[74	2013		Recto/perianal and vaginal	Y	52	10	19
**Eastern Europe**	Bulgaria	Kovachev[75]	2003	18wk/24wk	Vaginal	N	110	18	16

**Eastern Europe**	Czech	Motlova[76]	2004	delivery	Recto/perianal and vaginal	Y	586	172	29
**Eastern Europe**	Slovenia	Lucovnik[77]	2016	throughout (84% 35-	Vaginal	Y	1064	184	17
**Eastern Europe**	Slovenia	Lucovnik[77]	2016	throughout (84% 35-	Recto/perianal and vaginal	Y	464	73	16
**Eastern Europe**	Poland	Brzychczy-Wloch[78]	2013	35-37	Recto/perianal and vaginal	Y	3363	953	28
**Eastern Europe**	Poland	Romanik[79]	2014	37-40wks	Recto/perianal and vaginal	Y	80	23	29
**Eastern Europe**	Poland	Brzychczy-wloch [80]	2012	35-37	Recto/perianal and vaginal	Y	1176	353	30
**Eastern Europe**	Poland	Romanik[81]	2011	35-40	Recto/perianal and vaginal	Y	80	22	28
**Eastern Europe**	Poland	Kociszewska-Najman[82]	2010	>36 weeks	Recto/perianal and vaginal	Y	2212	252	11
**Eastern Europe**	Poland	lysakowska[83]	2011	35-37	Recto/perianal and vaginal	Y	105	31	30
**Eastern Europe**	Poland	Strus[84]	2009	as per CDC (35-37)	Recto/perianal and vaginal	Y	340	61	18
**Eastern Europe**	Poland	Krasnianin[85]	2009	delivery	Vaginal	Y	100	19	19
**Eastern Europe**	Poland	Brzychczy-wloch[86]	2009	35-37	Recto/perianal and vaginal	Y	1176	353	30
**Eastern Europe**	Poland	Brzychczy-wloch[87]	2008	3rd trimester	Recto/perianal and vaginal	Y	250	43	17
**Eastern Europe**	Poland	Brzychczy-wloch[87]	2008	3rd trimester	Vaginal	Y	223	30	13
**Eastern Europe**	Poland	Elzbieta[88]	2009	delivery	Recto/perianal and vaginal	Y	100	19	19
**Eastern Europe**	Poland	Kowalska[89]	2003	32-37	Recto/perianal and vaginal	Y	1678	331	20
**Eastern Europe**	Poland	Polish neonatal surveillance network	2016		Recto/perianal and vaginal	N	631	78	12
**Eastern Europe**	Poland	Pruss[90]	2015	35-37weeks	Recto/perianal and vaginal	Y	1111	250	23
**Eastern Europe**	Russia	Zatsiorskaya[91]	2014	12-18weeks	Recto/perianal and vaginal	N	491	30	6
**Melanesia**	Fiji	Gyaneshwar[92]	1987	<28 weeks	Vaginal	N	440	9	2
**Middle Africa**	Central	Brochet[93]	2009	third trimester	Vaginal	N	1000	175	18
**Middle Africa**	DRC	Mitima[94]	2014	3rd trim	Vaginal	Y	509	103	20
**Middle Africa**	Gabon	Capan-	2014	delivery	Recto/perianal and vaginal	Y	549	106	19

**Northern Africa**	Tunisia	Ferjani[96]	2005	3 groups 1st/2nd/3rd	Recto/perianal and vaginal	Y	300	39	13
**Northern Africa**	Tunisia	Ben Hamouda [97]	2008	labor >34wks	Vaginal	N	207	27	13
**Northern Africa**	Tunisia	Jerbi[98]	2007	term labor	Recto/perianal and vaginal	Y	294	38	13
**Northern Africa**	Egypt	Shabayek[99]	2009	35-40wks	Vaginal	Y	150	38	25
**Northern Africa**	Egypt	Abdelmoaty[100	2009	35-41wks	Recto/perianal and vaginal	Y	150	39	26
**Northern Africa**	Egypt	Sadaka[101]	2017		Recto/perianal and vaginal	Y	200	53	27
**Northern Africa**	Morocco	Mahmoud[102]	2010	Del	Recto/perianal and vaginal	Y	240	56	23
**Northern Africa**	Morocco	Benbachir[103]	1983	throughout	Recto/perianal and vaginal	Y	35	7	20
**Northern Africa**	Morocco	Bassat	2013	32 weeks – delivery	Recto/perianal and vaginal	Y	347	82	24
**Northern America**	USA	Towers[104]	2010	late3rd trim + delivery	Recto/perianal and vaginal	Y	1472	296	20
**Northern America**	USA	Panda[105]	2009	35-37	Recto/perianal and vaginal	Y	350	106	30
**Northern America**	USA	Turrentine[106]	2008	35-37	Recto/perianal and vaginal	Y	5198	1325	25
**Northern America**	USA	Chen[107]	2006	35-37	Recto/perianal and vaginal	Y	2963	743	25
**Northern America**	USA	Whitney[17]	2004	20-32	Vaginal	Y	69	15	22
**Northern America**	USA	Campbell[108]	2000	labor	Recto/perianal and vaginal	Y	3307	856	26
**Northern America**	USA	Bland[109]	2001	>35 weeks	Recto/perianal and vaginal	Y	2111	574	27
**Northern America**	USA	Lin[110]	2011	>32 weeks	Recto/perianal and vaginal	Y	5497	1031	19
**Northern America**	Canada	Delport[111]	2009	?	Recto/perianal and vaginal	Y	1460	322	22
**Northern America**	Canada	Spaetgens[112]	2002	35-37	Recto/perianal and vaginal	Y	1207	235	19
**Northern America**	Canada	Lavergne[113]	2006	35-37	Recto/perianal and vaginal	Y	949	201	21
**Northern America**	Canada	Wenman[114]	2001		Vaginal	N	1672	184	11
**Northern America**	Canada	Davies[115]	2001	36	Recto/perianal and vaginal	Y	1207	235	19
**Northern Europe**	Lithuania	Barcaite[116]	2012	labor 5.3% <34 wks	Recto/perianal and vaginal	N	970	148	15
**Northern Europe**	Iceland	Bjarnadottir[117]	2003	23-36	Recto/perianal and vaginal	Y	280	68	24
**Northern Europe**	Ireland	Whitney[17]	2004	20-32	Vaginal	Y	203	24	12
**Northern Europe**	Denmark	Stokholm[118]	2014	36	Vaginal	Y	442	44	10
**Northern Europe**	Denmark	Hansen[119]	2004	36-41	Recto/perianal and vaginal	Y	58	22	38
**Northern Europe**	UK	Hassan[120]	2011	34-40	Recto/perianal and vaginal	Y	100	19	19
**Northern Europe**	UK	Afshar[121]	2011	34-37	Recto/perianal and vaginal	Y	650	170	26
**Northern Europe**	UK	Jones[122]	2006	from 34 wks	Recto/perianal and vaginal	Y	748	159	21

**Northern Europe**	Norway	Brigtsen[123]	2015	35-37wks	Recto/perianal and vaginal	Y	1682	439	26
**Northern Europe**	Sweden	Hakansson[124]	2008	delivery	Recto/perianal and vaginal	Y	1569	356	23
**South America**	Brazil	Castellano-Filho[125]	2010	labor	Recto/perianal and vaginal	Y	221	21	10
**South America**	Brazil	Zusman[126]	2006	35-37	Vaginal	Y	598	107	18
**South America**	Brazil	Pogere[127]	2011	35 or more	Recto/perianal and vaginal	Y	273	59	22
**South America**	Brazil	Benchetrit[128]	1982	labor	Recto/perianal and vaginal	Y	86	22	26
**South America**	Brazil	Giraldo[129]	2012	in labor - (49 preterm)	Recto/perianal and vaginal	Y	45	7	16
**South America**	Brazil	Rocchetti[130]	2011	35-37	Recto/perianal and vaginal	Y	405	103	25
**South America**	Brazil	Linhares[131]	2011	20 weeks or more	Recto/perianal and vaginal	Y	213	21	10
**South America**	Brazil	Pires[132]	2010	>32wks	Recto/perianal and vaginal	Y	198	30	15
**South America**	Brazil	Costa[133]	2008	pregnancy at term , mean 39 weeks 4	Recto/perianal and vaginal	Y	201	41	20
**South America**	Brazil	Marconi[134]	2010	35-37 weeks	Recto/perianal and vaginal	Y	405	103	25
**South America**	Brazil	Borger[135]	2005	32-41 weeks	Recto/perianal and vaginal	Y	167	32	19
**South America**	Brazil	Kiss[136]	2013	not described	Recto/perianal and vaginal	Y	105	16	15
**South America**	Brazil	Simoes[137]	2007	delivery	Recto/perianal and vaginal	Y	316	46	15
**South America**	Brazil	Beitune[138]	2007	35-37	Recto/perianal and vaginal	Y	101	20	20
**South America**	Brazil	Beitune[139]	2006	35-37	Recto/perianal and vaginal	Y	106	15	14
**South America**	Brazil	Benchetrit[140]	1981	delivery	Recto/perianal and vaginal	Y	42	11	26
**South America**	Brazil	Chaves[141]	2008	35wks	Recto/perianal and vaginal	Y	102	25	25
**South America**	Brazil	Nunes[142]	2015	34-37 mainly	Recto/perianal and vaginal	N	144	58	40
**South America**	Brazil	Siqueira[143]	2015	32-37	Recto/perianal and vaginal	Y	411	58	14
**South America**	Argentin	Ronchi[144]	2011	35-37	Recto/perianal and vaginal	Y	324	21	6
**South America**	Argentin	Oviedo[145]	2013	35-37	Recto/perianal and vaginal	Y	3125	293	9
**South America**	Argentin	Quiroga[146]	2008	35-37	Recto/perianal and vaginal	Y	1105	84	8
**South America**	Argentin	Larcher[147]	2005	35-37	Recto/perianal and vaginal	Y	1228	17	1
**South America**	Argentin a	Di Bartolomeo [148]	2005	>35wks	Recto/perianal and vaginal	Y	1203	113	9
**South America**	Argentin	Toresani[149]	2001	26-40	Vaginal	Y	531	17	3
**South America**	Chile	Abarzua[150]	2014	35-37	Recto/perianal and vaginal	Y	1181	167	14

**South America**	Chile	Valdes[151]	2003	35-37	Recto/perianal and vaginal	N	1658	102	6
**South America**	Peru	Tamariz Ortiz[152]	2004	26-40 weeks	Recto/perianal and vaginal	Y	238	26	11
**South America**	Uruguay	Laufer[153]	2009	32-41	Recto/perianal and vaginal	Y	300	52	17
**South America**	Venezuel	Amesty[154]	2007	not described	Recto/perianal and vaginal	Y	100	18	18
**South America**	Venezuel	Riera[154]	1993	Term	Vaginal	Y	171	56	33
**South America**	Venezuel a	Pina-Carruyo [155]	1979	2nd/3rd trimester	Vaginal	Y	122	34	28
**South America**	Colombi	Garcia[156]	2011	35-37.6	Recto/perianal and vaginal	N	130	1	1
**South America**	Colombi	Ceballos[157]	2014	mean 35.4	Recto/perianal and vaginal	Y	182	32	18
**South America**	Paragua	Ortiz[158]	2013	35 -37 weeks	Recto/perianal and vaginal	Y	203	48	24
**South-eastern Asia**	Thailand	kovavisarach[15	2007	28-42	Recto/perianal and vaginal	Y	320	58	18
**South-eastern Asia**	Thailand	Werawatakul[16	2001	labor	Vaginal	Y	901	56	6
**South-eastern Asia**	Thailand	Tor-Udom[161]	2006	35-37wks	Recto/perianal and vaginal	Y	406	65	16
**South-eastern Asia**	Thailand	Whitney[17]	2004	20-32	Vaginal	Y	200	24	12
**South-eastern Asia**	Thailand	Whitney[17]	2004	20-32	Vaginal	Y	200	29	15
**South-eastern Asia**	Myanmar	Turner[162]	2012	during labor	Recto/perianal and vaginal	Y	549	47	9
**South-eastern Asia**	Thailand	Kovavisarach[1	2008	35-37	Recto/perianal and vaginal	Y	302	47	16
**South-eastern Asia**	Myanmar	Whitney[17]	2004	20-32	Vaginal	Y	226	16	7
**South-eastern Asia**	Vietnam	Goto[163]	2005	throughout pregnancy	Vaginal	N	505	22	4
**South-eastern Asia**	Malaysia	Raj[164]	2009	35-37	Recto/perianal and vaginal	N	56	18	32
**South-eastern Asia**	Malaysia	Lim[165]	1997	delivery	Vaginal	Y	196	19	10
**South-eastern Asia**	Singapor	Chua[166]	1995	throughout	Vaginal	N	326	46	14
**South-eastern Asia**	Singapor	Chow[167]	1981	delivery	Vaginal	Y	204	36	18
**South-eastern Asia**	Philippin	Whitney[17]	2004	20-32wks	Vaginal	Y	200	15	8
**Southern Africa**	South	Cutland[168]	2009	Labor	Vaginal	Y	3964	830	21
**Southern Africa**	South	Kwatra[169]	2014	20-25 / 26-30 / 31-35	Recto/perianal and vaginal	Y	521	148	28
**Southern Africa**	South	Cutland	2016	delivery	Vaginal	Y	4649	811	17
**Southern Africa**	South	Bolukaoto[170]	2015	16-38wks	Recto/perianal and vaginal	Y	413	128	31
**Southern Africa**	South	Madzivhandila[1	2011	Labor	Vaginal	Y	2561	551	22
**Southern Africa**	South	Monyama[172]	2016	>16 weeks	Recto/perianal and vaginal	Y	413	128	31

**Southern Africa**	South	Chukwu[173]	2015	16-38	Recto/perianal and vaginal	Y	413	128	31
**Southern Africa**	South	Dangor[174]	2016	26-37	Recto/perianal and vaginal	Y	284	72	25
**Southern Asia**	India	Sharmila[175]	2011	35-37	Recto/perianal and vaginal	Y	300	7	2
**Southern Asia**	India	Kulkarni[176]	2001	delivery	Recto/perianal and vaginal	Y	317	8	3
**Southern Asia**	India	Hajare[177]	2012	29-40wks	Vaginal	N	200	15	8
**Southern Asia**	India	Madhavi[178]	2011	3rd trim	Vaginal	N	200	15	8
**Southern Asia**	India	Goyal[179]	2004	35-37	Vaginal	Y	304	4	1
**Southern Asia**	India	Dalal[180]	1999	throughout pregnancy	Vaginal	Y	507	49	10
**Southern Asia**	India	Mani[181]	1984	delivery	Vaginal	Y	325	19	6
**Southern Asia**	India	Kishore[182]	1986	delivery	Vaginal	N	212	1	0
**Southern Asia**	India	Nagar[183]	2007	35-77 or delivery	Vaginal	Y	150	19	13
**Southern Asia**	India	Rajaratnam[184	2013	35-37wks	Vaginal	N	349	29	8
**Southern Asia**	India	Konikkara[185]	2014	35-37weeks	Recto/perianal and vaginal	Y	50	8	16
**Southern Asia**	India	Konikarra	2013	35-37 weeks	Vaginal	Y	150	19	13
**Southern Asia**	India	Muthusami[186]	2007	3rd trimester	Vaginal	N	77	4	5
**Southern Asia**	India	Gaind	2016	>37weeks	Recto/perianal and vaginal	Y	400	38	10
**Southern Asia**	India	Anthony	2016	35-37 weeks	Recto/perianal and vaginal	N	72	18	25
**Southern Asia**	India	Kumar	2016	>34 weeks	Recto/perianal and vaginal	Y	657	42	6
**Southern Asia**	India	Chaudhary[187]	1981	delivery	Vaginal	Y	100	16	16
**Southern Asia**	India	Dechen[188]	2010		Vaginal	N	524	25	5
**Southern Asia**	India	Das[189]	2003	delivery	Vaginal	Y	200	15	8
**Southern Asia**	India	Patil[190]	2013	delivery >35weeks	Recto/perianal and vaginal	Y	905	110	12
**Southern Asia**	India	Khatoon[191]	2016	delivery	Recto/perianal and vaginal	Y	300	6	2
**Southern Asia**	India	Chaudhary[191]	2016	3rd trimester	Recto/perianal and vaginal	Y	300	45	15
**Southern Asia**	Pakistan	Chaudhry[192]	2010	delivery >37wks	Vaginal	Y	200	17	9
**Southern Asia**	Pakistan	Kirmani[193]	1994	delivery	Vaginal	Y	60	7	12
**Southern Asia**	Pakistan	Ahktar[194]	1987	delivery	Vaginal	Y	202	47	23
**Southern Asia**	Pakistan	Hafeez[195]	1997	3rd trimester	Vaginal	N	200	9	5
**Southern Asia**	Pakistan	Munir[196]	2016	3rd trimester	Vaginal	N	200	28	14
**Southern Asia**	Banglad	Chan[197]	2013	>30	Recto/perianal and vaginal	Y	1219	94	8
**Southern Asia**	Banglad	Saha	2016	>30 weeks, delivery	Recto/perianal and vaginal	Y	1166	172	15

**Southern Asia**	Iran	Hadavand[198]	2015	35-37	Recto/perianal and vaginal	N	210	7	3
**Southern Asia**	Iran	Tajbakhsh[199]	2013	>35wks	Vaginal	Y	285	27	9
**Southern Asia**	Iran	Shirazi[200]	2014	35-37	Vaginal	N	980	48	5
**Southern Asia**	Iran	Absalan[201]	2013	?	Recto/perianal and vaginal	N	250	49	20
**Southern Asia**	Iran	Jahromi[202]	2008	>24wks in labor	Recto/perianal and vaginal	Y	1197	110	9
**Southern Asia**	Iran	Hamedi[203]	2012	in labor (8% <34wks)	Recto/perianal and vaginal	N	200	12	6
**Southern Asia**	Iran	Seyyed[204]	2013	>37 weeks	Recto/perianal and vaginal	Y	178	36	20
**Southern Asia**	Iran	Hassanzadeh[2	2011	labor- mean 38.2	Recto/perianal and vaginal	Y	310	43	14
**Southern Asia**	Iran	Moghaddam[20	2010	3rd trim	Recto/perianal and vaginal	Y	201	25	12
**Southern Asia**	Iran	Fatemi[207]	2010	labor	Vaginal	Y	330	68	21
**Southern Asia**	Iran	Mansouri[208]	2008	35-37	Vaginal	Y	602	55	9
**Southern Asia**	Iran	Aali[209]	2007	term, delivery	Vaginal	N	105	7	7
**Southern Asia**	Iran	Rabiee[210]	2006	>20 weeks	Vaginal	N	544	145	27
**Southern Asia**	Iran	Bornasi[211]	2016	35-37	Vaginal	N	500	60	12
**Southern Asia**	Iran	Goudarzi[212]	2015	35-37	Recto/perianal and vaginal	Y	100	17	17
**Southern Europe**	Bosnia	Numanovic[213]	2017		Vaginal	N	100	7	7
**Southern Europe**	Croatia	Muller-Vranjes [214]	2011	35-37	Recto/perianal and vaginal	Y	59	12	20
**Southern Europe**	Croatia	Trischler-Ceke[215]	2010	35-37	Recto/perianal and vaginal	Y	404	59	15
**Southern Europe**	Spain	Leibana-Martos[216]	2015	35-37	Recto/perianal and vaginal	Y	1180	188	16
**Southern Europe**	Spain	Dadvand[217]	2011	36	Recto/perianal and vaginal	Y	7976	1359	17
**Southern Europe**	Spain	Marimon[218]	2005	35-37	Recto/perianal and vaginal	Y	7084	1276	18
**Southern Europe**	Spain	Ramos[219]	2009	35-37	Recto/perianal and vaginal	Y	1416	204	14
**Southern Europe**	Spain	Bayo[220]	2002	35-40	Vaginal	Y	623	44	7
**Southern Europe**	Spain	Mestres[221]	2008	from 24 weeks	Recto/perianal and vaginal	Y	5670	1028	18
**Southern Europe**	Spain	RojoBezares[22	2016	35-37	Recto/perianal and vaginal	Y	2730	375	14
**Southern Europe**	Italy	Berardi[223]	2014	35-37	Vaginal	Y	3630	874	24
**Southern Europe**	Italy	Savoia[224]	2008	35-37	Recto/perianal and vaginal	Y	400	73	18
**Southern Europe**	Italy	Roccasalva[225	2008	35-37	Recto/perianal and vaginal	Y	60	7	12

**Southern Europe**	Italy	Busetti[226]	2007	35-37	Recto/perianal and vaginal	Y	5020	901	18
**Southern Europe**	Italy	Lijoi[227]	2007	35-37	Recto/perianal and vaginal	Y	1273	209	16
**Southern Europe**	Italy	De Luca[228]	2016	delivery	Recto/perianal and vaginal	Y	241	96	40
**Southern Europe**	Greece	Prifti[229]	2012	?	Vaginal	N	2793	93	3
**Southern Europe**	Greece	Daskalakis[230]	2006	22-25wks	Vaginal	N	1197	150	13
**Southern Europe**	Greece	Tsolia[231]	2003	>35wks or delivery	Recto/perianal and vaginal	Y	1014	67	7
**Western Africa**	Gambia	Suara[232]	1994	Del	Recto/perianal and vaginal	Y	136	30	22
**Western Africa**	Gambia	Le Doare[233]	2016	del	Recto/perianal and vaginal	Y	750	253	34
**Western Africa**	Nigeria	Uhiara[234]	1993	Del	Recto/perianal and vaginal	N	100	14	14
**Western Africa**	Nigeria	Dawodu[235]	1983	Del	Vaginal	Y	225	44	20
**Western Africa**	Nigeria	Onipede[236]	2012	35-40wks	Vaginal	Y	150	20	13
**Western Africa**	Nigeria	Olanisebe[237]	1986	28-36wks	Vaginal	Y	500	8	2
**Western Africa**	Nigeria	Onile[238]	1980	delivery	Vaginal	Y	388	71	18
**Western Africa**	Nigeria	Onwuezobe[239	2016	35-37	Recto/perianal and vaginal	Y	150	2	1
**Western Africa**	Nigeria	Nwachukwu[240	2006	3rd trimester	Recto/perianal and vaginal	N	200	18	9
**Western Africa**	Senegal	Denis[241]	1979	delivery	Vaginal	N	100	6	6
**Western Africa**	Senegal	Brochet[93]	2009	third trimester	Vaginal	Y	797	159	20
**Western Africa**	Ghana	Vinnemeier[242]	2015	>35	Recto/perianal and vaginal	Y	103	24	23
**Western Africa**	Ghana	Vinnemeier[242]	2015	>35	Recto/perianal and vaginal	Y	399	73	18
**Western Africa**	Togo	Balaka[243]	2005	29-40	Vaginal	N	306	13	4
**Western Africa**	Togo	Mounerou[244]	2015	34-38	Vaginal	N	200	5	3
**Western Africa**	Togo	David-	1991	delivery	Recto/perianal and vaginal	Y	106	4	4
**Western Africa**	Ghana	Enweronu-Laryea[246]	2011	>28wks	Recto/perianal and vaginal	Y	100	19	19
**Western Africa**	Ivory	Faye-Kette[247]	1991	throughout	Vaginal	N	150	29	19
**Western Asia**	Turkey	Karadag[248]	2013	24-44	Recto/perianal and vaginal	Y	300	9	3
**Western Asia**	Turkey	Yenisehirli[249]	2006	35-37	Vaginal	Y	671	98	15
**Western Asia**	Turkey	Kadanali[250]	2005	22-40	Recto/perianal and vaginal	Y	150	48	32
**Western Asia**	Turkey	Eren[251]	2005	delivery	Recto/perianal and vaginal	Y	500	46	9
**Western Asia**	Turkey	Barbaros[252]	2005	delivery	Recto/perianal and vaginal	Y	300	24	8
**Western Asia**	Turkey	Yucesoy[253]	2004	35-37 - or preterm	Recto/perianal and vaginal	Y	200	13	7

**Western Asia**	Turkey	Arisoy[254]	2003	35-37	Recto/perianal and vaginal	Y	310	33	11
**Western Asia**	Turkey	Altoparlak[255]	2004	22-40 (mean31.4)	Vaginal	N	150	41	27
**Western Asia**	Turkey	Akman[256]	2001	35-37	Recto/perianal and vaginal	Y	100	10	10
**Western Asia**	Turkey	Celebi[257]	1992	delivery	Vaginal	Y	76	4	5
**Western Asia**	Turkey	gokalp[258]	1986	delivery	Recto/perianal and vaginal	Y	100	7	7
**Western Asia**	Turkey	gokalp[259]	1985	delivery	Recto/perianal and vaginal	Y	40	4	10
**Western Asia**	Turkey	gokalp[260]	1988	delivery >37 wks	Recto/perianal and vaginal	Y	110	9	8
**Western Asia**	Turkey	Ayata[261]	1994	labor	Recto/perianal and vaginal	Y	114	10	9
**Western Asia**	Turkey	Alp[262]	2016	?	Recto/perianal and vaginal	Y	215	21	10
**Western Asia**	Kuwait	Al-Sweih[263]	2005	delivery	Recto/perianal and vaginal	Y	847	124	15
**Western Asia**	Kuwait	Al-Sweih[264]	2004	35-37	Recto/perianal and vaginal	Y	110	18	16
**Western Asia**	Kuwait	Ghaddar[265]	2014	35-37	Recto/perianal and vaginal	N	1391	288	21
**Western Asia**	United	Sidky[266]	2002	delivery	Recto/perianal and vaginal	Y	891	192	22
**Western Asia**	United	Amin[267]	2002	delivery	Recto/perianal and vaginal	Y	563	57	10
**Western Asia**	Saudi	Zamzami[268]	2011	delivery	Recto/perianal and vaginal	Y	326	103	32
**Western Asia**	Saudi	El-Kersh[269]	2002	>28 wks	Recto/perianal and vaginal	Y	217	66	30
**Western Asia**	Saudi Arabia	Gosling and Morgos[270]	1983	delivery	Recto/perianal and vaginal	Y	115	16	14
**Western Asia**	Saudi	uduman[271]	1985	delivery	Vaginal	N	260	24	9
**Western Asia**	Saudi	Al-	1991	33wks	Recto/perianal and vaginal	Y	1939	334	17
**Western Asia**	Saudi	Khan[273]	2015	>35 weeks	Vaginal	Y	1328	178	13
**Western Asia**	Lebanon	Seoud[274]	2010	del - mean 38+4	Recto/perianal and vaginal	N	775	137	18
**Western Asia**	Lebanon	Chaaya[275]	1996	delivery 32-42wks	Vaginal	N	166	13	8
**Western Asia**	Lebanon	Ghaddar[265]	2014	35-37	Vaginal	Y	168	31	18
**Western Asia**	Jordan	Sunna[276]	1991	3rd trimester	Recto/perianal and vaginal	Y	500	152	30
**Western Asia**	Israel	Eisenberg[277]	2006	labor	Recto/perianal and vaginal	Y	629	86	14
**Western Asia**	Israel	Marchaim[278]	2003	>35 wks delivery	Recto/perianal and vaginal	Y	681	84	12
**Western Asia**	Israel	Eidelman[279]	1990	delivery	Vaginal	Y	446	17	4
**Western Asia**	Israel	Drai-Hasid[280]	2015	delivery	Recto/perianal and vaginal	Y	436	77	18
**Western Europe**	France	Chhin[281]	2013	34-38	Vaginal	N	3046	471	15

**Western Europe**	France	Van Der Mee-Marquet[282]	2009	35-38	Vaginal	Y	500	39	8
**Western Europe**	France	Honderlick[283]	2010	3rd trim	Vaginal	Y	11718	1663	14
**Western Europe**	France	Mereghetti[284]	2007	34-38wks	Vaginal	N	1460	87	6
**Western Europe**	France	Chhuy[285]	2005	>34wks	Vaginal	N	1674	116	7
**Western Europe**	France	Jaureguy[286]	2003	35-37wks	Recto/perianal and vaginal	Y	370	57	15
**Western Europe**	France	Volumenie[287]	2001	35-37	Vaginal	N	3906	559	14
**Western Europe**	Belgium	El Aila[288]	2009	35-37	Recto/perianal and vaginal	Y	150	36	24
**Western Europe**	Germany	Kunze[289]	2015	delivery	Recto/perianal and vaginal	Y	784	133	17
**Western Europe**	Germany	Kunze[290]	2011	35-37	Recto/perianal and vaginal	Y	869	183	21
**Western Europe**	Germany	Brimil[291]	2006	?	Recto/perianal and vaginal	Y	210	34	16
**Western Europe**	Netherlands	Valkenberg-van den Berg[292]	2006	35-37	Recto/perianal and vaginal	Y	1702	365	21
**Western Europe**	Switzerla	Capanna[293]	2013	35-37weeks	Recto/perianal and vaginal	Y	760	124	16
**Western Europe**	Switzerla	Rausch[294]	2009	?	Recto/perianal and vaginal	Y	1316	276	21
**Western Europe**	Austria	Hafner[295]	1998	34 weeks	Recto/perianal and vaginal	Y	3569	520	15
**Western Europe**	Reunion	Dahan-	2011	>24wks	Vaginal	N	17430	2911	17

Supplementary Table S5: Study Characteristics: Maternal GBS Colonization in pregnancy with serotype data.

**Region**	**Country**	**Author**	**Year**	**No. of isolates**	**Serotypes**								
					**Ia/Ib**	**Ia**	**Ib**	**II**	**III**	**IV**	**V**	**VI-IX**	**NT**
**Australia and New Zealand**	Australia	Taylor[297]	2006	19	7	5	2	0	9	0	0	1	2
**Australia and New Zealand**	Australia	Ko[298]	2015	408	218	187	31	40	142	5	113	6	9
**Australia and New Zealand**	New Zealand	Grimwood[299]	2002	52	21	11	10	3	15	0	10	2	0
**Central America**	Mexico	Gonzales[11]	2004	31	24	0	0	5	2	0	0	0	0
**Central America**	Mexico	Solorzano-Santos[12]	1989	33	22	19	3	4	1	0	0	0	6
**Central America**	Mexico	Gonzalez - Pedraza[10]	2002	101	62	62	0	26	13	0	0	0	0
**Central America**	Mexico	Ocampo-Torres[14]	2000	78	53	0	0	15	10	0	0	0	0
**Eastern Africa**	Zimbabwe	Moyo[18]	2000	92	13	10	3	1	38	3	34	0	2
**Eastern Africa**	Zimbabwe	Moyo[300]	2002	117	23	17	6	5	53	6	28	0	2
**Eastern Africa**	Malawi	Gray[21]	2011	390	95	71	24	40	152	1	93	3	6
**Eastern Africa**	Kenya	Seale[23]	2016	915	308	194	114	80	350	18	156	3	0
**Eastern Africa**	Mozambique	Madrid	2016	64	15	9	6	3	6	3	20	0	17
**Eastern Asia**	China	Wang[34]	2015	56	19	10	9	3	18	0	8	0	8
**Eastern Asia**	China	Lu[35]	2014	201	67	43	24	14	84	1	30	4	1
**Eastern Asia**	China	Shen[301]	2000	155	43	39	4	55	40	0	6	0	11
**Eastern Asia**	China	Shen[302]	1998	22	8	5	3	7	4	1	0	0	2
**Eastern Asia**	China	tjj[303]	2015	56	19	10	9	3	18	0	8	0	8
**Eastern Asia**	China	Yan[304]	2016	231	76	52	24	12	83	0	49	5	6

**Eastern Asia**	China	Lu[305]	2015	160	51	33	18	10	72	0	23	1	3
**Eastern Asia**	China	Van Elzakker[303]	2009	58	18	13	5	2	19	0	15	2	0
**Eastern Asia**	S.Korea	Seo[306]	2010	145	42	26	16	8	51	0	35	9	0
**Eastern Asia**	S.Korea	Lee[307]	2010	315	68	38	30	14	138	0	64	26	5
**Eastern Asia**	S.Korea	Lee[63]	2010	318	72	35	37	19	111	0	87	29	0
**Eastern Asia**	S.Korea	Hong[64]	2010	177	35	23	12	10	63	4	43	0	22
**Eastern Asia**	S.Korea	Oh[308]	2009	42	13	11	2	2	12	0	11	4	0
**Eastern Asia**	S.Korea	Uh[65]	1997	29	21	7	14	0	6	0	0	0	2
**Eastern Asia**	S.Korea	Lee[307]	2010	318	72	35	37	19	111	0	87	29	0
**Eastern Asia**	Japan	Wakimoto[309]	2011	198	59	26	33	13	15	1	17	91	2
**Eastern Asia**	Japan	kimura[310]	2013	139	36	10	26	18	17	2	29	38	1
**Eastern Asia**	Japan	terakubo[68]	2002	187	28	16	12	3	19	0	16	105	0
**Eastern Asia**	Japan	Matsubara[69]	2002	48	10	4	6	4	5	0	3	22	0
**Eastern Asia**	Japan	Morozumi[71]	2015	154	56	24	32	7	26	5	27	32	1
**Eastern Europe**	Romania	Cristea[311]	2011	257	63	51	12	29	84	7	60	0	14
**Eastern Europe**	Romania	Usein[312]	2009	13	3	1	2	5	3	0	2	0	0
**Eastern Europe**	Romania	Usein[312]	2009	39	12	6	6	7	11	6	3	0	0
**Eastern Europe**	Czech Republic	Motlova[76]	2004	172	38	38	0	0	57	0	24	0	0
**Eastern Europe**	Poland	Romanik[313]	2014	23	10	10	0	0	5	0	7	0	0
**Eastern Europe**	Poland	Brzychczy-wloch[80]	2012	353	99	71	28	53	123	17	61	0	0
**Eastern Europe**	Poland	Brzychczy-wloch[314]	2010	100	32	25	7	14	29	7	18	0	0
**Eastern Europe**	Poland	Wolski[315]	2009	100	30	0	0	7	32	0	0	0	9
**Eastern Europe**	Poland	Brzychczy-wloch[86]	2009	353	99	71	28	53	124	18	60	0	0

**Middle Africa**	Central African Republic	Brochet[93]	2009	88	31	24	7	20	15	0	22	0	0
**Middle Africa**	Gabon	Belard[316]	2015	109	39	14	25	7	30	0	33	0	0
**Northern Africa**	Algeria	Bergal[317]	2015	44	2	2	0	11	10	0	21	0	0
**Northern Africa**	Morocco	Benbachir[103]	1983	15	7	6	1	3	4	0	0	0	1
**Northern America**	USA	Campbell[318]	2000	856	289	225	64	155	183	0	179	2	11
**Northern America**	USA	Croak[318]	2003	145	36	25	11	17	24	5	46	1	16
**Northern America**	Canada	Davies[115]	2001	118	34	24	10	10	18	0	33	0	23
**Northern America**	Canada	Davies[115]	2001	233	78	53	25	30	48	5	45	1	26
**Northern America**	Canada	Teatero[116]	2017	102	36	24	12	13	26	6	20	1	0
**Northern Europe**	Lithuania	Barcaite[116]	2012	148	51	44	7	16	51	13	11	3	3
**Northern Europe**	Ireland	Meehan[319]	2014	18	5	4	1	3	7	0	2	1	0
**Northern Europe**	Ireland	Whitney[17]	2004	20	8	7	1	1	6	0	4	0	1
**Northern Europe**	Ireland	Dore[320]	2003	87	23	15	8	13	30	2	14	0	5
**Northern Europe**	UK	Jones[321]	2006	159	66	41	25	15	42	0	30	0	0
**Northern Europe**	Norway	Brigsten[322]	2015	426	109	67	42	59	106	0	72	17	3
**Northern Europe**	Sweden	Hakansson[124]	2008	356	85	39	46	57	85	53	68	5	3
**Northern Europe**	Sweden	Berg[323]	2000	114	30	15	15	13	36	3	25	0	7
**South America**	Brazil	Benchetrit[324]	1982	31	20	10	10	5	3	0	0	0	3
**South America**	Brazil	Soares[325]	2013	39	6	4	2	3	15	0	10	0	0
**South America**	Brazil	Palmeiro[326]	2010	30	17	12	5	6	1	1	2	0	3
**South America**	Brazil	Simoes[137]	2007	47	20	9	11	9	3	3	4	0	8
**South America**	Argentina	Oviedo[145]	2013	112	55	45	10	11	24	0	13	5	4
**South-eastern Asia**	Thailand	Whitney[17]	2004	24	5	5	0	3	3	0	12	0	1
**South-eastern Asia**	Thailand	Whitney[17]	2004	28	5	4	1	4	6	0	2	9	2
**South-eastern Asia**	Myanmar	Turner[162]	2012	66	12	11	1	16	8	4	8	19	3
**South-eastern Asia**	Myanmar	Whitney[17]	2004	14	2	2	0	5	0	0	5	0	2
**South-eastern Asia**	Malaysia	Dhanoa[327]	2010	200	26	23	3	11	24	20	38	47	34
**South-eastern Asia**	Malaysia	Eskandarian[17]	2015	49	6	6	0	5	2	0	9	27	0
**South-eastern Asia**	Malaysia	Suhaimi[328]	2017	12	7	7	0	0	2	0	2	1	0
**South-eastern Asia**	Philippines	Whitney[17]	2004	15	2	1	1	4	5	0	2	1	1

**Southern Africa**	South Africa	Kwatra[169]	2014	507	134	120	14	22	93	0	36	0	0
**Southern Africa**	South Africa	Cutland	2016	811	365	305	60	77	210	20	113	0	26
**Southern Africa**	South Africa	Madzivhandila[171]	2011	541	199	163	36	61	202	20	55	0	4
**Southern Africa**	South Africa	Chukwu[173]	2015	128	44	33	11	20	38	11	14	0	0
**Southern Africa**	South Africa	Dangor[174]	2016	72	39	39	0	0	14	0	12	0	0
**Southern Asia**	India	Mani[181]	1984	19	10	4	6	8	1	0	0	0	0
**Southern Asia**	India	Kumar	2016	54	41	29	12	0	3	0	3	0	2
**Southern Asia**	India	Chaudhary	2016	45	8	6	2	9	10	0	9	3	6
**Southern Asia**	Bangladesh	Chan[197]	2013	94	26	23	3	7	9	0	28	20	4
**Southern Asia**	Bangladesh	Saha	2016	172	71	69	2	24	20	1	40	16	0
**Southern Asia**	Iran	Beigverdi[329]	2014	41	3	0	3	6	27	0	2	0	2
**Southern Asia**	Iran	jannati[330]	2012	56	9	4	5	7	6	7	11	12	4
**Southern Asia**	Iran	Bornasi[331]	2016	60	15	11	4	10	27	0	8	0	0
**Southern Asia**	Iran	Sadeh[332]	2016	30	7	5	2	6	15	0	2	0	0
**Southern Europe**	Portugal	Martins[331]	2000	269	56	42	14	46	59	6	59	5	38
**Southern Europe**	Spain	Leibana-Martos[216]	2015	188	48	42	6	31	52	8	35	9	5
**Southern Europe**	Spain	Puertas[333]	2010	150	38	38	0	15	50	11	21	3	10
**Southern Europe**	Spain	Liebana [334]	2010	188	46	38	8	23	53	11	31	12	12
**Southern Europe**	Spain	Perez-Ruiz[335]	2003	31	13	9	4	4	12	0	2	0	0
**Southern Europe**	Spain	Rojo-Bezares[222]	2016	65	16	8	8	9	22	2	16	0	0
**Southern Europe**	Italy	Savoia[336]	2008	73	21	16	5	4	23	6	19	0	0
**Southern Europe**	Greece	Daskalakis[230]	2006	67	21	13	8	18	15	2	6	5	0
**Western Africa**	Gambia	Suara[232]	1994	32	6	0	0	9	2	1	12	0	2
**Western Africa**	Gambia	Le Doare[233]	2016	237	36	20	16	39	24	0	130	0	0
**Western Africa**	Senegal	Brochet[93]	2009	75	17	13	4	9	16	0	33	0	0
**Western Africa**	Ghana	Vinnemeier[242]	2015	24	7	2	5	3	7	0	5	0	2
**Western Africa**	Ghana	Vinnemeier[242]	2015	72	28	25	3	6	14	0	21	0	3
**Western Asia**	Turkey	Ekin[337]	2015	56	9	9	0	10	7	2	0	7	21

**Western Asia**	Turkey	Yenisehirli[249]	2006	98	31	7	24	2	33	3	18	0	11
**Western Asia**	Turkey	Ekin[338]	2006	72	24	24	0	0	0	6	4	0	23
**Western Asia**	Turkey	Eren[251]	2005	54	16	14	2	16	10	1	0	0	11
**Western Asia**	Kuwait	Udo[339]	2013	154	21	16	5	16	30	5	59	6	17
**Western Asia**	Kuwait	boswihi[340]	2012	143	16	11	5	16	30	5	55	5	16
**Western Asia**	Kuwait	Al-Sweih[263]	2005	124	14	11	3	10	33	1	27	17	22
**Western Asia**	United Arab Emirates	Amin[267]	2002	57	14	12	2	2	10	15	7	0	9
**Western Asia**	Lebanon	Seoud[274]	2010	137	29	20	9	15	22	1	31	0	39
**Western Asia**	Lebanon	Hannoun[341]	2009	76	14	0	0	10	15	6	9	22	0
**Western Asia**	Israel	Marchaim[342]	2006	72	18	10	8	22	15	2	10	1	4
**Western Asia**	Israel	Bisharat[343]	2005	104	21	12	9	23	26	0	18	0	16
**Western Europe**	France	Van Der Mee-Marquet[344]	2009	39	13	10	3	0	16	0	7	0	0
**Western Europe**	France	Lamy[345]	2006	42	12	5	7	3	22	0	2	0	3
**Western Europe**	Belgium	El Aila[288]	2009	122	26	13	13	13	25	15	25	0	18
**Western Europe**	Germany	Kunze[289]	2015	165	52	40	12	25	46	8	32	2	0
**Western Europe**	Germany	Kunze[290]	2011	156	54	25	29	19	43	9	23	0	6
**Western Europe**	Germany	Von Both[346]	2003	146	37	26	11	22	43	4	19	0	21
**Western Europe**	Netherlands	Muller[347]	2008	39	8	0	0	3	2	2	2	0	0
**Western Europe**	Netherlands	Van Elzakker[348]	2009	92	30	24	6	12	20	7	14	7	0
**Western Europe**	Switzerland	Frohlicher[349]	2014	364	95	70	25	38	107	14	93	17	0

Supplementary Table S6: Maternal GBS colonization prevalence, by country

Country	Pregnant women tested (n)	Crude Prevalence (%)	95% CI	Selective methods only (%)	95% CI2	Adjusted (%)	95% CI3
**Argentina**	7516	6.2	2.7-9.7	6.8	2.7-11.0	6.4	2.9-10.0
**Australia**	2129	23.8	18.1-29.6	23.8	18.1-29.6	23.8	18.1-29.6
**Austria**	3569	14.6	13.4-15.7	14.6	13.4-15.7	14.6	13.4-15.7
**Bangladesh**	2385	11.2	4.3-18.1	11.2	4.3-18.1	11.2	4.3-18.1
**Belgium**	150	24	16.9-31.1	24	16.9-31.1	24	16.9-31.1
**Bosnia and Herzegovina**	100	7	1.5-12.5				
**Brazil**	4340	19.2	16.3-22.1	18.2	15.5-21.0	20.8	16.9-24.7
**Bulgaria**	110	16.4	9.1-23.7				
**Canada**	6495	18.6	13.9-23.3	20.5	19.2-21.9	20.5	19.2-21.9
**Central African Republic**	1000	17.5	15.1-19.9				
**Chile**	2839	10.1	2.3-17.9	14.1	12.1-16.2	14.1	12.1-16.2
**China**	44716	9.2	7.0-11.3	9.8	8.4-11.2	11.3	9.7-12.9
**Colombia**	312	9	0.0-25.4	17.6	11.8-23.4	17.6	11.8-23.4
**Croatia**	463	15.2	11.8-18.5	15.2	11.8-18.5	15.2	11.8-18.5
**Cuba**	120	27.5	19.2-35.8	27.5	19.2-35.8		
**Czech Republic**	586	29.4	25.6-33.1	29.4	25.6-33.1	29.4	25.6-33.1
**DRC**	509	20.2	16.7-23.8			28.7	24.7-32.7
**Denmark**	500	23.2	0.0-50.6	37.9	24.9-51.0	25.1	1.7-48.5
**Dominican Republic**	207	43.5	36.5-50.4	43.5	36.5-50.4	43.5	36.5-50.4
**Egypt**	500	26	22.0-30.0	26.3	21.5-31.1	29.2	23.2-35.2

**Ethiopia**	1154	12.4	8.3-16.6	12.3	7.5-17.0	13.5	8.7-18.2
**Fiji**	440	2	0.6-3.5				
**France**	22674	11.4	8.3-14.5	15.4	11.6-19.2	15.6	9.3-21.9
**inc Reunion Island**	40102	12.1	9.2-14.9			20.7	9.6-31.8
**Gabon**	549	19.3	15.9-22.7	19.3	15.9-22.7	19.3	15.9-22.7
**Gambia**	886	28.4	16.9-39.8	28.4	16.9-39.8	28.4	16.9-39.8
**Germany**	1863	18.4	15.2-21.5	18.4	15.2-21.5	18.4	15.2-21.5
**Ghana**	602	19.1	15.9-22.4	19.1	15.9-22.4	19.1	15.9-22.4
**Greece**	5004	7.4	2.3-12.5	6.6	5.0-8.2	13.4	4.1-22.8
**Guatemala**	990	15.7	13.3-18.0	15.7	13.3-18.0	15.7	13.3-18.0
**Hungary**	345	27.2	22.4-32.1			38	28.1-47.9
**Iceland**	280	24.3	19.1-29.5	24.3	19.1-29.5	24.3	19.1-29.5
**India**	6599	7.6	5.7-9.5	7.4	4.1-10.7	9.6	6.8-12.3
**Iran**	5992	12.4	9.3-15.5	13.8	9.8-17.8	15.7	11.6-19.7
**Ireland**	203	11.8	7.1-16.5			16.7	11.4-22.1
**Israel**	2192	11.8	5.6-17.9	14.3	11.5-17.1	12.1	7.1-17.2
**Italy**	10624	21.3	17.1-25.4	20.6	15.9-25.2	23.2	15.3-31.0
**Ivory Coast**	150	19.3	12.7-25.9				
**Japan**	7238	11.1	9.1-13.1			16.2	12.1-20.2
**Jordan**	500	30.4	26.3-34.5	30.4	26.3-34.5	30.4	26.3-34.5
**Kenya**	7967	11.5	9.0-14.0	11.5	9.0-14.0	11.5	9.0-14.0
**Kuwait**	2348	17.4	12.6-22.2	14.8	12.5-17.1	14.8	12.5-17.1
**Lebanon**	1109	14.6	7.8-21.4			26.2	19.3-33.1
**Lithuania**	970	15.3	12.9-17.6				
**Malawi**	1954	20.4	17.5-23.4	21	19.1-22.9	21.2	19.3-23.0
**Malaysia**	252	20.1	0.0-42			13.8	8.7-18.8
**Mexico**	2239	9.1	5.7-12.5			17.9	11.3-24.5
**Morocco**	622	23.3	19.9-26.7	23.3	19.9-26.7	23.3	19.9-26.7
**Mozambique**	433	11.4	0.0-30.5	11.4	0.0-30.5	11.4	0.0-30.5

**Myanmar**	775	8.1	6.1-10.1	8.6	6.1-11.0	8.9	6.8-11.0
**Netherlands**	1702	21.4	19.5-23.4	21.4	19.5-23.4	21.4	19.5-23.4
**New Zealand**	240	21.7	16.3-27.1	21.7	16.3-27.1	21.7	16.3-27.1
**Nigeria**	1713	10.7	5.1-16.3	1.3	0.0-3.6	14.5	6.3-22.7
**Norway**	1682	26.1	24.0-28.2	26.1	24.0-28.2	26.1	24.0-28.2
**Pakistan**	862	12.1	5.8-18.5			19.9	6.1-33.8
**Paraguay**	203	23.6	17.6-29.7	23.6	17.6-29.7	23.6	17.6-29.7
**Peru**	238	10.9	6.8-15.1	10.9	6.8-15.1	10.9	6.8-15.1
**Philippines**	200	7.5	3.6-11.4			10.5	6.0-15.0
**Poland**	12625	21.5	17.2-25.8	23.2	18.4-28.1	23.1	18.7-27.6
**Reunion Island**	17430	16.7	16.1-17.3			35.8	35.1-36.5
**Russia**	491	6.1	3.9-8.3			9.2	6.5-11.8
**S.Korea**	11335	8.3	7.4-9.2	8.3	7.4-9.2	8.3	7.4-9.2
**Saudi Arabia**	4185	18.9	13.8-24.0	23.2	14.7-31.7	22.1	17.2-27.0
**Senegal**	897	13.2	0.0-27.0			28.4	25.2-31.5
**Singapore**	530	15.4	12.0-18.7			25	18.8-31.2
**Slovenia**	1528	16.8	14.9-18.7	15.7	12.3-19.1	20.2	11.6-28.8
**South Africa**	13218	25.3	22.1-28.5	29.5	27.4-31.5	28.9	26.6-31.2
**Spain**	26679	15	12.9-17.2	16.3	14.9-17.7	15.5	13.8-17.2
**Sweden**	1569	22.7	20.6-24.8	22.7	20.6-24.8	22.7	20.6-24.8
**Switzerland**	2076	18.7	14.1-23.3	18.7	14.1-23.3	18.7	14.1-23.3
**Tanzania**	595	16.1	2.9-29.4	23	18.1-27.9	23	18.1-27.9
**Thailand**	2329	13.6	8.7-18.4	16.5	14.1-18.8	15.7	11.4-20.0
**Togo**	612	3.4	1.8-5.0	3.8	0.0-7.9	6.5	1.5-11.4
**Trinidad**	810	32.1	28.8-35.4	32.1	28.8-35.4	32.1	28.8-35.4
**Tunisia**	801	13	10.6-15.4	13	10.1-15.8	17.4	9.6-25.2
**Turkey**	3336	10.8	7.8-13.8	9.6	6.7-12.5	10.5	7.1-13.8
**UK**	1498	22.8	18.8-26.9	22.8	18.8-26.9	22.8	18.8-26.9

**USA**	20967	24.3	21.5-27.1	24.4	21.6-27.3	24.7	21.9-27.5
**Ukraine**	52	19.2	7.8-30.7	19.2	7.8-30.7		
**United Arab Emirates**	1454	15.8	4.6-27.0	15.8	4.6-27.0	15.8	4.6-27.0
**Uruguay**	300	17.3	12.9-21.8	17.3	12.9-21.8	17.3	12.9-21.8
**Venezuela**	393	26.3	17.6-34.9	18	10.0-26.0	34.5	17.3-51.7
**Vietnam**	505	4.4	2.5-6.2				
**Zimbabwe**	1968	29.9	20.1-39.6	39.3	23.2-55.3	34.6	21.6-47.7
**Overall**	300,176	15.2	14.4-16.0	17.4	16.4-18.5	18	16.9-19.1

Supplementary Table S7: Maternal GBS colonization prevalence: comparison of sample site.

country	Author	Year	Total tested	Positive with Vaginal swabs alone	Positive with vaginal and rectal swabs included
**Australia**	Gilbert[1]	2002	1096	238	296
**Australia**	Law[350]	2013	278	52	64
**Austria**	Hafner[295]	1998	3569	326	520
**Belgium**	El Aila[288]	2009	150	28	36
**Belgium**	El Aila[351]	2010	100	9	17
**Czech Republic**	Motlova[76]	2004	586	127	172
**France**	Jaureguy[286]	2003	370	41	90
**Greece**	Tsolia[231]	2003	1014	60	67
**India**	Sharmila[175]	2011	300	2	7
**India**	Kulkarni[176]	2001	317	3	8
**India**	Patil[190]	2013	905	68	110
**India**	Chaudhary[191]	2016	300	32	45
**Iran**	Moghaddam[206]	2010	201	22	25
**Iran**	Goudarzi[352]	2015	100	14	17
**Ireland**	Kieran[353]	1998	501	100	126
**Malawi**	Dzowela[22]	2005	97	14	16
**Netherlands**	Hoogkamp-Korstanje[354]	1982	762	60	102
**Poland**	Brzychczy-wloch[80]	2012	1176	313	353
**Saudi Arabia**	Zamzami[268]	2011	326	95	103

**South Africa**	Kwatra[169]	2014	521	99	148
**Thailand**	Kovavisarach[355]	2007	320	43	58
**USA**	Dillon[356]	1982	2540	434	895
**USA**	Philipson[357]	1995	94	17	29
**USA**	Jamie[358]	2004	200	55	67
**USA**	Quinlan[359]	2000	222	44	54
**USA**	Badri[360]	1977	789	81	162
**USA**	Platt[361]	1995	870	105	146
**Zimbabwe**	Moyo[18]	2000	206	52	65

Supplementary Table S8: Maternal GBS Colonization prevalence: studies included in comparison of culture methods

(Selective enrichment with conventional selective agar alone*)

Country	Author	Year	Comparison*	Total tested	Positive with Selective agar alone	Positive with addition of selective enrichment
**Belgium**	El Aila[351]	2010	SE vs SA	100	13	17
**Belgium**	El Aila[288]	2009	SE vs SA	150	22	45
**Canada**	Elsayed[362]	2003	SE vs SA	639	98	125
**China**	Xie[57]	2016	SE vs SA	200	16	20
**South Africa**	Monyama[172]	2016	SE vs SA	413	58	128
**Spain**	Bosch-Mestres[363]	2003	SE vs SA	388	32	54
**Spain**	Bosch-Mestres[363]	2003	SE vs SA	473	57	75
**USA**	Silver[364]	1996	SE vs SA	1222	209	293
**USA**	Orsello[365]	2003	SE vs SA	145	21	35
**Venezuela**	Diaz[366]	2008	SE vs SA	60	19	21

*SA=Conventional selective agar of low sensitivity (e.g. Colombia CNA or NNA)

Supplementary Table S9: Maternal GBS colonization prevalence: studies included in comparison of culture methods

Selective enrichment with unselective agar alone

Country	Author	Year	Total tested	Positive with Non selective agar alone	Positive with Addition of Selective Enrichment
**Canada**	Elsayed[362]	2003	639	79	125
**China**	Xie[57]	2016	200	12	20
**Ireland**	Thinkhamrop[367]	2003	203	7	24
**Myanmar**	Thinkhamrop[367]	2003	226	1	16
**Thailand**	Thinkhamrop[367]	2003	200	6	24
**Thailand**	Thinkhamrop[367]	2003	200	16	29
**USA**	Altaie[368]	1994	952	86	166
**USA**	Philipson[357]	1995	383	50	76
**USA**	Nguyen[369]	1998	524	55	87
**USA**	Baker[370]	1976	460	43	82
**USA**	Mason[371]	1976	54	4	12
**USA**	Platt[361]	1995	651	35	78
**USA**	Thinkhamrop[367]	2003	68	11	13

*A = Non-selective agar

Supplementary Table S10: Maternal GBS serotype distribution by UN sub-region

Region	No of Samples	Serotype prevalence (%)							
		**Ia/Ib****	**Ia**	**Ib**	**II**	III	IV	V	VI/VII/VIII/IX*
Australia and New Zealand	468	48	33	11	7	35	1	16	2
Central America	237	70	63	4	20	10	0	0	0
Eastern Africa	1551	25	18	8	6	36	2	27	0
Eastern Asia	2937	30	16	12	7	29	0	16	11
Eastern Europe	1387	28	21	6	11	34	3	15	0
Middle Africa	197	36	20	15	14	22	0	28	0
Northern Africa	58	25	22	0	24	24	0	24	0
Northern America	1378	34	25	9	15	22	2	26	0
Northern Europe	1306	30	20	10	13	29	4	17	1
South America	241	50	30	17	13	16	0	10	1
South-eastern Asia	365	18	16	1	14	12	3	20	20
Southern Africa	2029	39	34	5	8	27	2	11	0
Southern Asia	553	32	22	7	14	25	0	14	6
Southern Europe	966	27	21	6	14	29	4	18	2
Western Africa	433	25	17	6	14	16	0	40	0
Western Asia	958	25	18	6	14	23	4	19	3
Western Europe	1117	29	19	10	11	28	4	16	1
Overall	16181	32	21	8	11	25	1	18	2

*Also includes NT6, II-IV-X, R, and JM9

** 7 studies did not differentiate between Ia and Ib

Supplementary Table S11: Maternal GBS serotype distribution by country

Country	No of Samples	Serotype prevalence (%)							
		**Ia/Ib****	**Ia**	**Ib**	**II**	III	IV	V	VI/VII/VIII/IX*
Algeria	**44**	5	5	0	25	23	0	48	0
Argentina	**108**	51	42	9	10	22	0	12	5
Australia	**416**	53	42	8	6	40	1	15	2
Bangladesh	**262**	36	33	2	11	11	0	26	15
Belgium	**104**	25	13	13	13	24	14	24	0
Brazil	**133**	50	27	21	16	14	1	10	0
Canada	**404**	37	25	12	13	23	2	25	0
Central African Republic	**88**	35	27	8	23	17	0	25	0
China	**900**	33	23	11	11	36	0	14	1
Czech Republic	**172**	22	22	0	0	33	0	14	0
France	**78**	32	18	12	3	49	0	11	0
Gabon	**109**	36	13	23	6	28	0	30	0
Gambia	**267**	16	8	7	21	10	0	50	0
Germany	**440**	32	20	11	15	30	5	17	0
Ghana	**91**	38	23	11	10	23	0	28	0
Greece	**67**	31	19	12	27	22	3	9	8
India	**110**	51	31	18	20	11	0	8	1
Iran	**181**	17	10	8	16	44	2	11	3
Ireland	**119**	30	22	8	13	36	2	17	0
Israel	**156**	25	14	11	29	26	1	18	0
Italy	**73**	29	22	7	6	32	8	26	0
Japan	**722**	26	11	15	6	11	1	12	39
Kenya	**915**	34	21	13	9	38	2	17	0
Kuwait	**366**	14	10	4	11	25	3	38	7
Lebanon	**174**	24	20	9	14	21	4	22	14
Lithuania	**145**	35	30	5	11	35	9	8	2

Malawi	**384**	25	19	6	10	40	0	24	1
Malaysia	**227**	21	21	1	7	10	5	22	31
Mexico	**237**	70	63	4	20	10	0	0	0
Morocco	**14**	50	43	7	21	29	0	0	0
Mozambique	**47**	32	19	13	6	13	6	43	0
Myanmar	**75**	19	17	1	28	7	5	24	15
Netherlands	**131**	28	26	7	11	13	7	10	4
New Zealand	**52**	40	21	19	6	29	0	19	4
Norway	**423**	26	16	10	14	25	0	17	4
Philippines	**14**	14	7	7	29	36	0	14	7
Poland	**920**	29	22	7	11	34	4	15	0
Portugal	**231**	24	18	6	20	26	3	26	2
Romania	**295**	26	19	9	17	33	5	17	0
South Korea	**1315**	29	14	11	5	37	0	22	6
Senegal	**75**	23	17	5	12	21	0	44	0
South Africa	**2029**	39	34	5	8	27	2	11	0
Spain	**595**	27	22	4	14	32	5	17	3
Sweden	**460**	25	12	13	15	28	9	20	1
Switzerland	**364**	26	19	7	10	29	4	26	5
Thailand	**49**	20	18	1	14	18	0	29	16
Turkey	**214**	37	28	7	13	20	4	6	1
UK	**159**	42	26	16	9	26	0	19	0
USA	**974**	32	24	8	17	21	2	28	0
United Arab Emirates	**48**	29	25	4	4	21	31	15	0
Zimbabwe	**205**	17	13	4	2	44	4	31	0
Overall	**16181**	32	21	8	11	25	1	18	2

*Also includes NT6, II-IV-X, R, and JM9
** 7 Studies did not differentiate between Ia and Ib
Non-typeable isolates excluded

Supplementary Figure S1: Global distribution of maternal GBS colonization serotype data

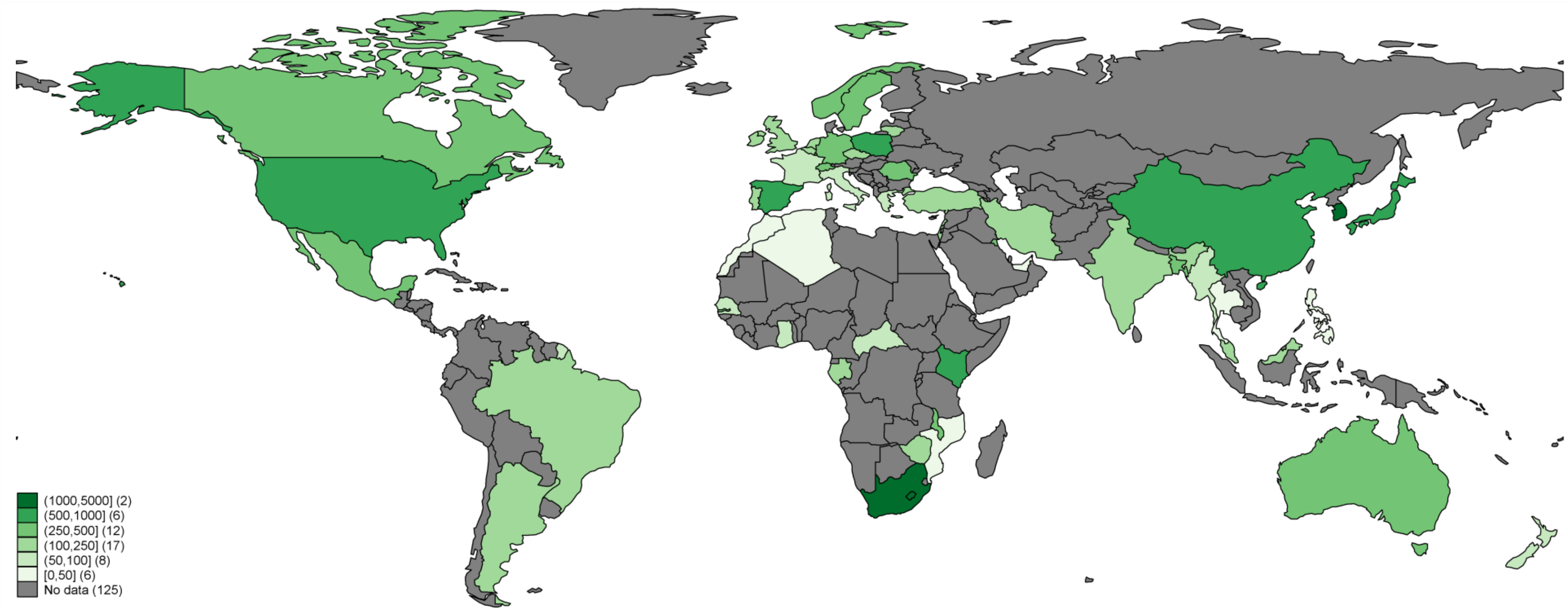

Supplementary Figure S2: GBS Colonization prevalence: Setting described as rural

Country	Year	Author	Number of women tested	GBS positive	ES (95% CI)	% Weight
Africa						
Zimbabwe	2006	Mavenyengwa	100	60	0.60 (0.50, 0.70)	11.40
Kenya	2016	Seale	526	47	0.09 (0.07, 0.12)	15.47
Mozambique	2016	Madrid	320	68	0.21 (0.17, 0.26)	14.63
Ghana	2015	Vinnemeier	399	73	0.18 (0.15, 0.22)	14.96
Subtotal (I-squared = 97.3%, p = 0.000)					0.26 (0.13, 0.39)	56.45
.						
Latin America and the Caribbean						
Cuba	2014	Cruz	120	33	0.28 (0.20, 0.36)	12.46
Subtotal (I-squared = .%, p = .)					0.28 (0.19, 0.36)	12.46
.						
Asia						
Bangladesh	2016	Saha	1166	172	0.15 (0.13, 0.17)	15.59
Myanmar	2012	Turner	549	47	0.09 (0.06, 0.11)	15.50
Subtotal (I-squared = 93.1%, p = 0.000)					0.12 (0.06, 0.18)	31.09
.						
Overall (I-squared = 95.7%, p = 0.000)					0.21 (0.15, 0.27)	100.00

NOTE: Weights are from random effects analysis

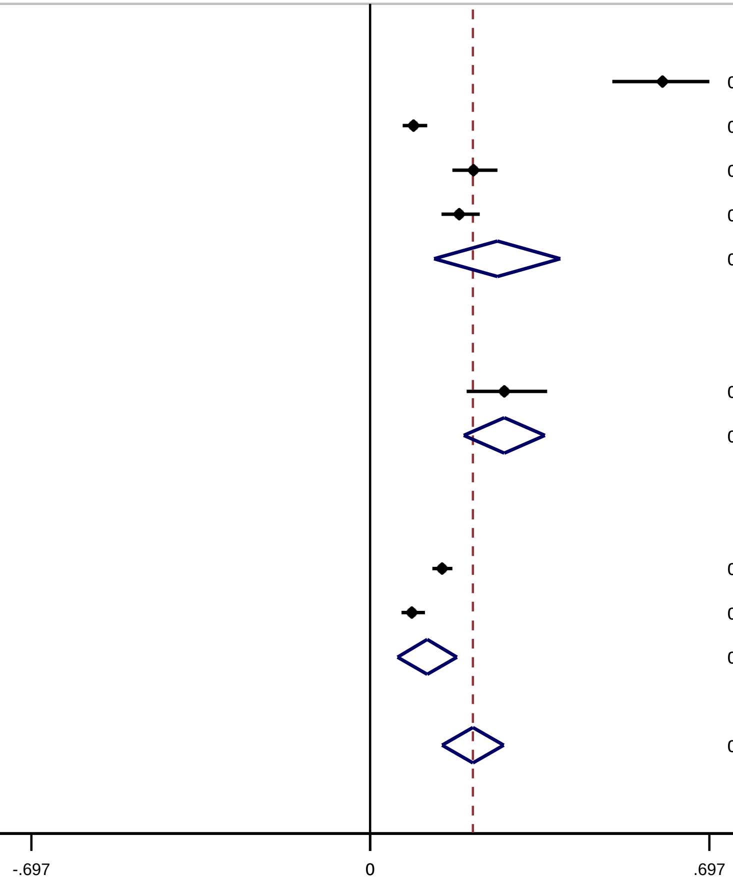

Supplementary Figure S3: GBS Colonization prevalence: setting described as mixed rural/urban

Country	Year	Author	Number of women tested	GBS positive	ES (95% CI)	% Weight
Africa						
South Africa	2016	Monyama	413	128	0.31 (0.27, 0.36)	4.08
South Africa	2015	Chukwu	413	128	0.31 (0.27, 0.36)	4.08
Zimbabwe	2010	Mavenyengwa	672	142	0.21 (0.18, 0.24)	4.28
Zimbabwe	2006	Mavenyengwa	300	138	0.46 (0.40, 0.52)	3.87
Zimbabwe	2000	Moyo	206	65	0.32 (0.25, 0.38)	3.73
Kenya		Seale	5470	608	0.11 (0.10, 0.12)	4.46
Subtotal (I-squared = 98.4%, p = 0.000)					0.28 (0.18, 0.39)	24.50
.						
Latin America and the Caribbean						
Brazil	2011	Linhares	213	21	0.10 (0.06, 0.15)	4.13
Brazil	2008	Lima Dos Reis	201	41	0.20 (0.15, 0.27)	3.87
Brazil	2010	Marconi	405	103	0.25 (0.21, 0.30)	4.11
Brazil	2007	Simoes	316	46	0.15 (0.11, 0.19)	4.16
Mexico	2000	Ocampo-Torres	910	78	0.09 (0.07, 0.11)	4.41
Trinidad	2003	Orrett	201	66	0.33 (0.26, 0.40)	3.70
Subtotal (I-squared = 94.8%, p = 0.000)					0.18 (0.11, 0.25)	24.38
.						
Asia						
Thailand	2001	Werawatakul	901	56	0.06 (0.05, 0.08)	4.42
Turkey	1994	Ayata	114	10	0.09 (0.04, 0.16)	3.90
Subtotal (I-squared = 0.0%, p = 0.392)					0.06 (0.05, 0.08)	8.32
.						
Developed Regions						
Japan	2015	Morozumi	1226	154	0.13 (0.11, 0.15)	4.40
Australia	2002	Gilbert	1096	296	0.27 (0.24, 0.30)	4.33
Lithuania	2012	Barcaite	970	148	0.15 (0.13, 0.18)	4.37
Netherlands	2006	Valkenberg-van den Berg	1702	365	0.21 (0.20, 0.23)	4.40
Poland	2009	Elzbieta	100	19	0.19 (0.12, 0.28)	3.42
UK	2006	Jones	748	159	0.21 (0.18, 0.24)	4.30
Sweden	2008	Hakansson	1569	356	0.23 (0.21, 0.25)	4.39
Greece	2003	Tsolia	1014	67	0.07 (0.05, 0.08)	4.43
USA	2010	Towers	1472	296	0.20 (0.18, 0.22)	4.39
Canada	2001	Davies	1207	235	0.19 (0.17, 0.22)	4.37
Subtotal (I-squared = 97.1%, p = 0.000)					0.18 (0.14, 0.23)	42.80
.						
Overall (I-squared = 97.4%, p = 0.000)					0.20 (0.17, 0.23)	100.00

NOTE: Weights are from random effects analysis

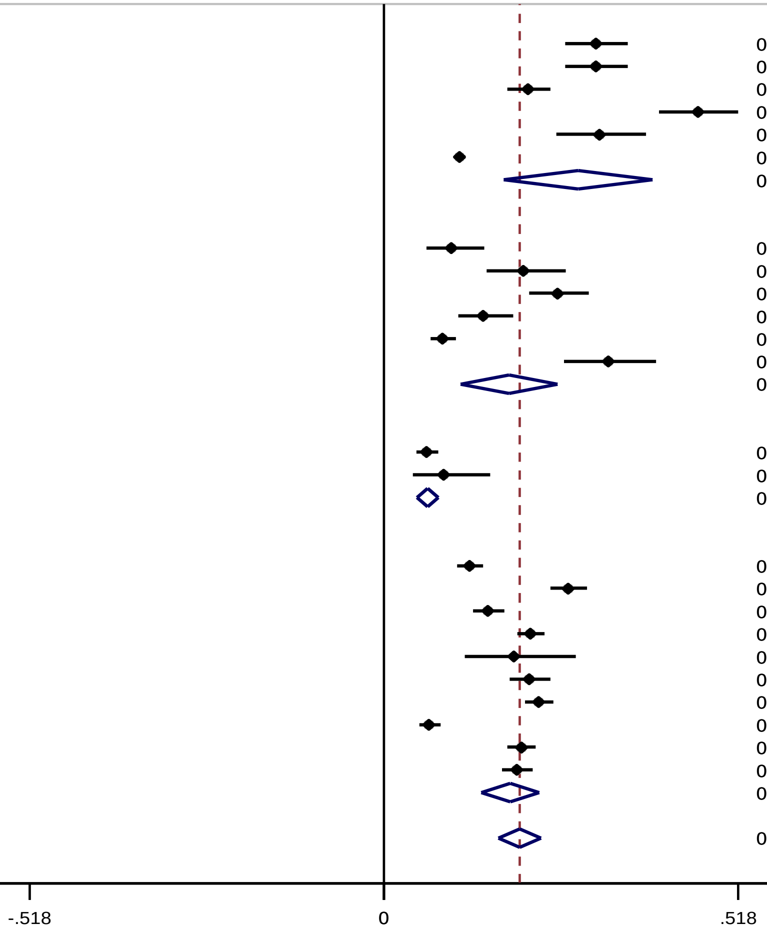

Supplementary Figure S4: Risk ratio for comparison between sampling site (inclusion of rectal swab sample).

Country	Year	Author	Total samples	Positive Vaginal samples	Positive Vagino-rectal samples	RR (95% CI)	% Weight
Africa							
South Africa	2014	Kwatra	521	99	148	1.49 (1.20, 1.87)	4.71
Zimbabwe	2000	Moyo	206	52	65	1.25 (0.92, 1.70)	3.88
Malawi	2005	Dzowela	97	14	16	1.14 (0.59, 2.21)	1.68
Subtotal (I-squared = 0.0%, p = 0.550)						1.39 (1.16, 1.65)	10.27
.							
Asia							
India	2011	Sharmila	300	2	7	3.50 (0.73, 16.71)	0.39
India	2001	Kulkarni	317	3	8	2.67 (0.71, 9.96)	0.53
India	2013	Patil	905	68	110	1.62 (1.21, 2.16)	4.08
India	2016	Chaudhary	300	32	45	1.41 (0.92, 2.15)	2.93
Iran	2010	Moghaddam	201	22	25	1.14 (0.66, 1.95)	2.22
Iran	2015	Goudarzi	100	14	17	1.21 (0.63, 2.33)	1.71
Saudi Arabia	2011	Zamzami	326	95	103	1.08 (0.86, 1.37)	4.62
Thailand	2007	Kovavisarach	320	43	58	1.35 (0.94, 1.94)	3.41
Subtotal (I-squared = 10.2%, p = 0.351)						1.32 (1.13, 1.54)	19.88
.							
Developed Regions							
Australia	2002	Gilbert	1096	238	296	1.24 (1.07, 1.44)	5.41
Czech Republic	2004	Motlova	586	127	172	1.35 (1.11, 1.65)	4.95
France	2003	Jaureguy	370	41	90	2.20 (1.56, 3.08)	3.60
Belgium	2009	El Aila	150	28	36	1.29 (0.83, 1.99)	2.82
Ireland	1998	Kieran	501	100	126	1.26 (1.00, 1.59)	4.63
Austria	1998	Hafner	3569	326	520	1.60 (1.40, 1.82)	5.56
Poland	2012	Brzychczy-wloch	1176	313	353	1.13 (0.99, 1.28)	5.58
Greece	2003	Tsolia	1014	60	67	1.12 (0.80, 1.56)	3.63
USA	1982	Dillon	2540	434	895	2.06 (1.86, 2.28)	5.78
Australia	2013	Law	278	52	64	1.23 (0.89, 1.71)	3.73
USA	1995	Philipson	94	17	29	1.71 (1.01, 2.89)	2.29
USA	2004	Jamie	200	55	67	1.22 (0.90, 1.64)	3.98
Belgium	2010	El Aila	100	9	17	1.89 (0.88, 4.03)	1.36
USA	2000	Quinlan	222	44	54	1.23 (0.86, 1.74)	3.50
Netherlands	1982	Hoogkamp-Korstanje	762	60	102	1.70 (1.26, 2.30)	3.94
USA	1977	Badri	789	81	162	2.00 (1.56, 2.56)	4.47
USA	1995	Platt	870	105	146	1.39 (1.10, 1.75)	4.62
Subtotal (I-squared = 81.6%, p = 0.000)						1.46 (1.28, 1.66)	69.85
.							
Overall (I-squared = 73.1%, p = 0.000)						1.42 (1.29, 1.57)	100.00

NOTE: Weights are from random effects analysis

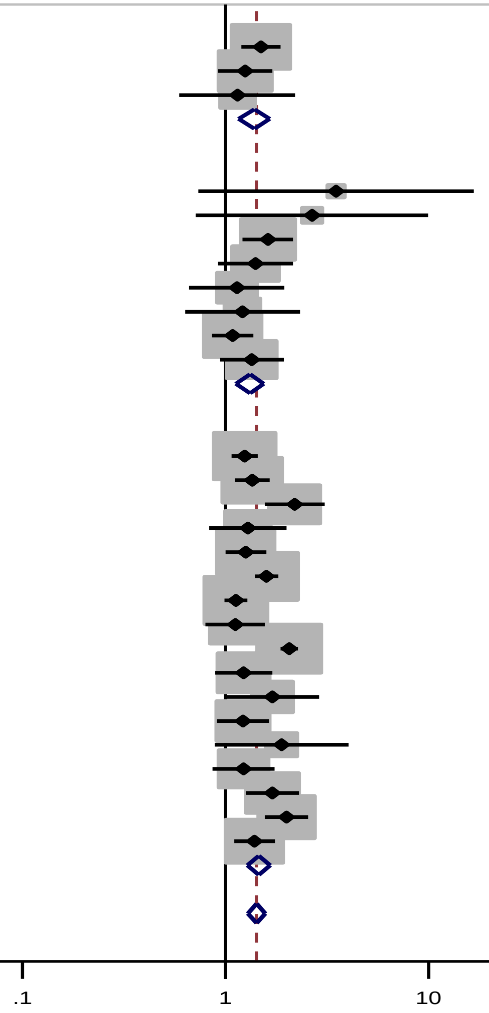

Supplementary Figure S5: Risk ratio for selective enrichment compared to selective conventional agar* alone without enrichment.

Country	Year	Author	Total samples	Positive with selective agar alone	Positive with selective enrichment	RR (95% CI)	% Weight
Africa							
South Africa	2016	Monyama	413	58	128	2.21 (1.67, 2.92)	13.83
Subtotal (I-squared = .%, p = .)						2.21 (1.67, 2.92)	13.83
.							
Developed Regions							
USA	1996	Silver	1222	209	293	1.40 (1.20, 1.64)	21.58
USA	2003	Orsello	145	21	35	1.67 (1.02, 2.72)	6.55
Belgium	2010	El Aila	100	13	17	1.31 (0.67, 2.55)	3.94
Canada	2003	Elsayed	639	98	125	1.28 (1.00, 1.62)	15.99
Spain	2003	Bosch-Mestres	388	32	54	1.69 (1.12, 2.55)	8.42
Spain	2003	Bosch-Mestres	473	57	75	1.32 (0.96, 1.81)	11.83
Belgium	2009	El Aila	150	22	45	2.05 (1.30, 3.23)	7.29
Subtotal (I-squared = 0.0%, p = 0.597)						1.42 (1.28, 1.59)	75.60
.							
Latin America and the Caribbean							
Venezuela	2008	Diaz	60	19	21	1.11 (0.67, 1.84)	6.21
Subtotal (I-squared = .%, p = .)						1.11 (0.67, 1.84)	6.21
.							
Asia							
China	2016	Xie	200	16	20	1.25 (0.67, 2.34)	4.37
Subtotal (I-squared = .%, p = .)						1.25 (0.67, 2.34)	4.37
.							
Overall (I-squared = 38.2%, p = 0.104)						1.51 (1.31, 1.74)	100.00

NOTE: Weights are from random effects analysis

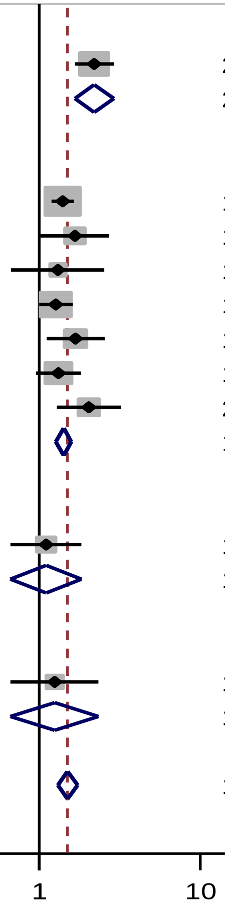

*Includes conventional agar with blood agar including antibiotics (Colombia CNA Agar (Colistin-Nalidixic Acid)), not including selective agars of proven high sensitivity (chromogenic agars, Strepto B etc)

Supplementary Figure S6: Risk ratio for detection with selective enrichment compared to unselective agar alone

Country	Year	Author	Total samples	Positive with non-selective agar alone	Positive with selective enrichment	RR (95% CI)	% Weight
Asia							
China	2016	Xie	200	12	20	1.67 (0.84, 3.32)	4.02
Thailand	2003	Thinkamrop	200	6	24	4.00 (1.67, 9.58)	2.63
Thailand	2003	Thinkamrop	200	16	29	1.81 (1.02, 3.23)	5.40
Myanmar	2003	Thinkamrop	226	1	16	16.00 (2.14, 119.63)	0.53
Subtotal (I-squared = 57.2%, p = 0.072)						2.62 (1.36, 5.04)	12.59
.							
Developed Regions							
USA	1994	Altaie	952	86	166	1.93 (1.51, 2.46)	16.68
USA	1995	Philipson	383	50	76	1.52 (1.10, 2.11)	12.25
USA	1998	Nguyen	524	55	87	1.58 (1.15, 2.17)	12.82
Canada	2003	Elsayed	639	79	125	1.58 (1.22, 2.05)	15.77
USA	1976	Baker	460	43	82	1.91 (1.35, 2.69)	11.47
USA	1976	Mason	54	4	12	3.00 (1.03, 8.72)	1.81
USA	1995	Platt	651	35	78	2.23 (1.52, 3.27)	10.02
Ireland	2003	Thinkamrop	203	7	24	3.43 (1.51, 7.78)	2.95
USA	2003	Thinkamrop	68	11	13	1.18 (0.57, 2.45)	3.63
Subtotal (I-squared = 8.4%, p = 0.365)						1.78 (1.56, 2.02)	87.41
.							
Overall (I-squared = 28.4%, p = 0.159)						1.85 (1.59, 2.14)	100.00

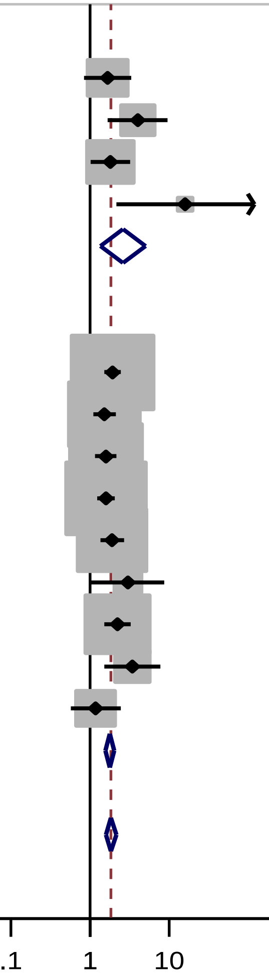

NOTE: Weights are from random effects analysis

Supplementary Figure S7: Maternal GBS prevalence by sub-region

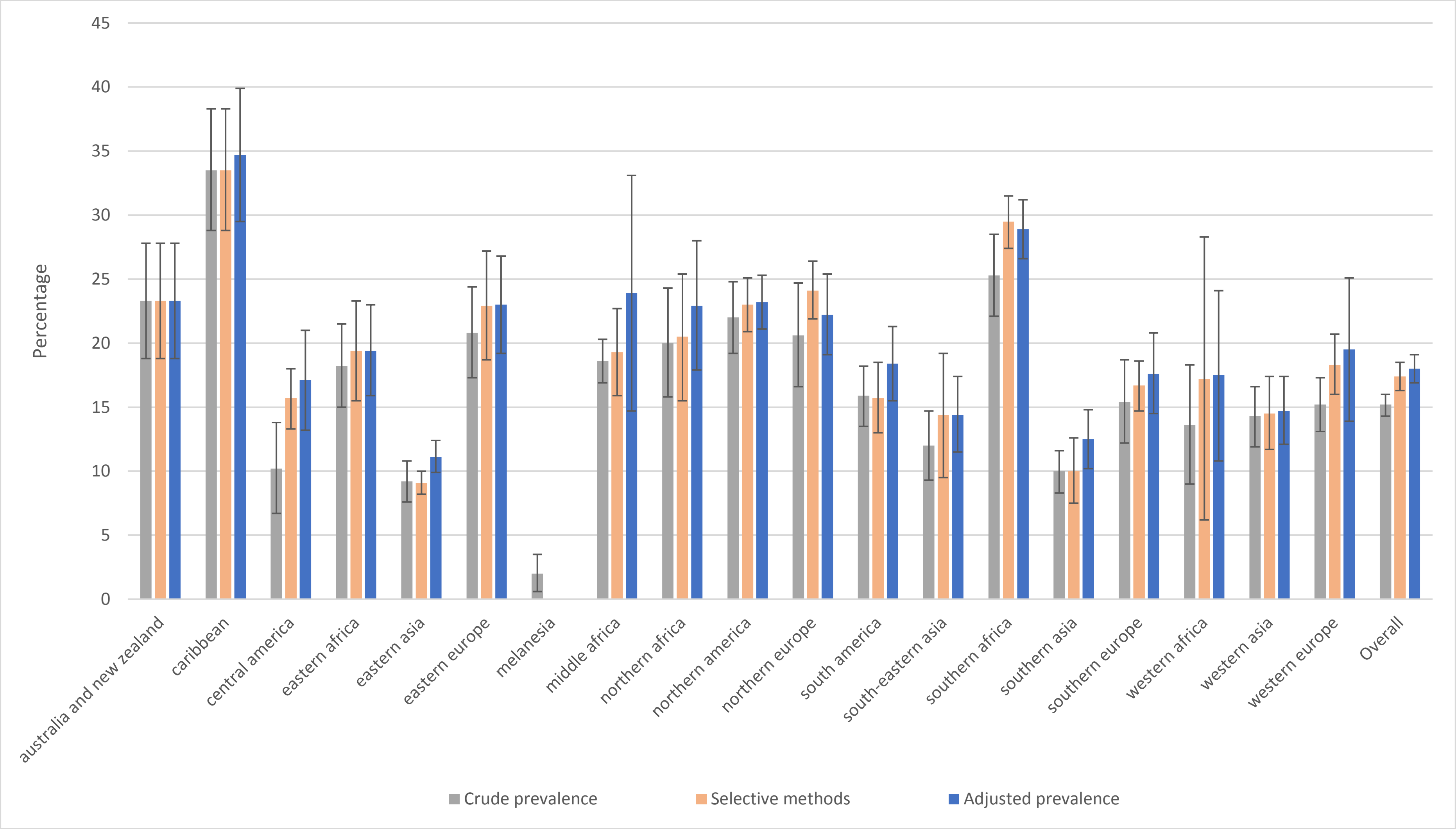

Supplementary Figure S8: Maternal GBS prevalence in developed region: adjusted

Country	Year	Author	No of women tested	Number of women positive (real or adjusted)	ES (95% CI)	% Weight
Eastern Europe						
Poland		Strus	340	61	0.18 (0.14, 0.22)	1.30
Poland	2003	Kowalska	1678	331	0.20 (0.18, 0.22)	1.39
Czech Republic	2004	Motlova	586	172	0.29 (0.26, 0.33)	1.32
Poland	2008	Brzychczy-wloch	250	43	0.17 (0.13, 0.22)	1.27
Poland	2008	Brzychczy-wloch	223	42	0.19 (0.14, 0.25)	1.25
Poland	2009	Krasnianin	100	27	0.27 (0.19, 0.37)	1.02
Poland	2009	Brzychczy-wloch	1176	353	0.30 (0.27, 0.33)	1.37
Poland	2009	Elzbieta	100	19	0.19 (0.12, 0.28)	1.08
Poland	2010	Kociszewska-Najman	2212	252	0.11 (0.10, 0.13)	1.40
Poland	2011	romanik	80	22	0.28 (0.18, 0.39)	0.95
Poland	2011	lysakowska	105	31	0.30 (0.21, 0.39)	1.02
Poland	2012	Brzychczy-wloch	1176	353	0.30 (0.27, 0.33)	1.37
Poland	2013	Brzychczy-Wloch	3363	953	0.28 (0.27, 0.30)	1.40
Poland	2014	Romanik	80	23	0.29 (0.19, 0.40)	0.94
Russia	2014	Zatsiorskaya	491	45	0.09 (0.07, 0.12)	1.37
Poland	2015	Pruss	1111	250	0.23 (0.20, 0.25)	1.37
Hungary	2015	Abrok	100	38	0.38 (0.28, 0.48)	0.97
Slovenia	2016	Lucovnik	1064	261	0.25 (0.22, 0.27)	1.37
Slovenia	2016	Lucovnik	464	73	0.16 (0.13, 0.19)	1.34
Subtotal (I-squared = 96.5%, p = 0.000)					0.23 (0.19, 0.27)	23.49
.						
Western Europe						
Austria	1998	Hafner	3569	520	0.15 (0.13, 0.16)	1.40
France	2003	Jaureguy	370	57	0.15 (0.12, 0.19)	1.32
Germany	2006	Brimil	210	34	0.16 (0.11, 0.22)	1.25
Netherlands	2006	Valkenberg-van den Berg	1702	365	0.21 (0.20, 0.23)	1.39
France	2009	Van Der Mee-Marquet	500	55	0.11 (0.08, 0.14)	1.36
Switzerland	2009	Rausch	1316	276	0.21 (0.19, 0.23)	1.38
Belgium	2009	El Aila	150	36	0.24 (0.17, 0.32)	1.14
France	2010	Honderlick	11718	2364	0.20 (0.19, 0.21)	1.41
France	2011	Dahan-Saal	17430	6238	0.36 (0.35, 0.37)	1.41
Germany	2011	Kunze	869	183	0.21 (0.18, 0.24)	1.36
Switzerland	2013	Capanna	760	124	0.16 (0.14, 0.19)	1.37
Germany	2015	Kunze	784	133	0.17 (0.14, 0.20)	1.37
Subtotal (I-squared = 99.3%, p = 0.000)					0.19 (0.14, 0.25)	16.16
.						
North America						
USA	2000	Campbell	3307	856	0.26 (0.24, 0.27)	1.40
Canada	2001	Davies	1207	235	0.19 (0.17, 0.22)	1.38
USA	2001	Bland	2111	574	0.27 (0.25, 0.29)	1.39
Canada	2002	Spaetgens	1207	235	0.19 (0.17, 0.22)	1.38
USA	2004	Whitney	69	21	0.30 (0.20, 0.43)	0.88
USA	2006	Chen	2963	743	0.25 (0.24, 0.27)	1.40
Canada	2006	Lavergne	949	201	0.21 (0.19, 0.24)	1.37
USA	2008	Turrentine	5198	1325	0.25 (0.24, 0.27)	1.40
USA	2009	Panda	350	106	0.30 (0.26, 0.35)	1.27
Canada	2009	Delport	1460	322	0.22 (0.20, 0.24)	1.38
USA	2010	Towers	1472	296	0.20 (0.18, 0.22)	1.38
USA	2011	Lin	5497	1031	0.19 (0.18, 0.20)	1.40
Subtotal (I-squared = 93.0%, p = 0.000)					0.23 (0.21, 0.25)	16.02
.						
Southern Europe						
Spain	2002	Bayo	623	62	0.10 (0.08, 0.13)	1.37
Greece	2003	Tsolia	1014	67	0.07 (0.05, 0.08)	1.39
Spain	2005	Marimon	7084	1276	0.18 (0.17, 0.19)	1.41
Greece	2006	Daskalakis	1197	321	0.27 (0.24, 0.29)	1.37
Italy	2007	Lijoi	1273	209	0.16 (0.14, 0.19)	1.38
Italy	2007	Busetti	5020	901	0.18 (0.17, 0.19)	1.40
Italy	2008	Roccasalva	60	7	0.12 (0.05, 0.23)	1.03
Spain	2008	Mestres	5670	1028	0.18 (0.17, 0.19)	1.40
Italy	2008	Savoia	400	73	0.18 (0.15, 0.22)	1.32
Spain	2009	Ramos	1416	204	0.14 (0.13, 0.16)	1.39
Croatia	2010	Trischler-Ceke	404	59	0.15 (0.11, 0.18)	1.33
Croatia	2011	Muller-Vranjes	59	12	0.20 (0.11, 0.33)	0.91
Spain	2011	Dadvand	7976	1359	0.17 (0.16, 0.18)	1.41
Greece	2012	Prifti	2793	199	0.07 (0.06, 0.08)	1.40
Italy	2014	Berardi	3630	1242	0.34 (0.33, 0.36)	1.40
Italy	2015	De Luca	241	96	0.40 (0.34, 0.46)	1.19
Spain	2015	Leibana-Martos	1180	188	0.16 (0.14, 0.18)	1.38
Spain	2016	Rojo-Bezares	2730	375	0.14 (0.12, 0.15)	1.40
Subtotal (I-squared = 98.6%, p = 0.000)					0.18 (0.15, 0.21)	23.90
.						
Japan						
Japan	2002	terakubo	1404	265	0.19 (0.17, 0.21)	1.38
Japan	2002	Matsubara	583	68	0.12 (0.09, 0.15)	1.37
Japan	2015	Morozumi	1226	218	0.18 (0.16, 0.20)	1.38
Subtotal (I-squared = 89.2%, p = 0.000)					0.16 (0.12, 0.20)	4.13
.						
Australia and New Zealand						
Australia	2002	Gilbert	1096	296	0.27 (0.24, 0.30)	1.37
New Zealand	2002	Grimwood	240	52	0.22 (0.17, 0.27)	1.24
Australia	2005	Hiller	865	168	0.19 (0.17, 0.22)	1.37
Australia	2006	Taylor	168	43	0.26 (0.19, 0.33)	1.16
Subtotal (I-squared = 81.4%, p = 0.001)					0.23 (0.19, 0.28)	5.13
.						
Northern Europe						
Iceland	2003	Bjarnadottir	280	68	0.24 (0.19, 0.30)	1.26
Denmark	2004	Hansen	58	22	0.38 (0.26, 0.52)	0.79
Ireland	2004	Whitney	203	34	0.17 (0.12, 0.23)	1.25
UK	2006	Jones	748	159	0.21 (0.18, 0.24)	1.35
Sweden	2008	Hakansson	1569	356	0.23 (0.21, 0.25)	1.38
UK	2011	Afshar	650	170	0.26 (0.23, 0.30)	1.34
UK	2011	Hassan	100	19	0.19 (0.12, 0.28)	1.08
Denmark	2014	Stokholm	442	62	0.14 (0.11, 0.18)	1.34
Norway	2015	Brigtsen	1682	439	0.26 (0.24, 0.28)	1.38
Subtotal (I-squared = 84.5%, p = 0.000)					0.22 (0.19, 0.25)	11.17
.						
Overall (I-squared = 98.3%, p = 0.000)					0.21 (0.19, 0.23)	100.00

NOTE: Weights are from random effects analysis

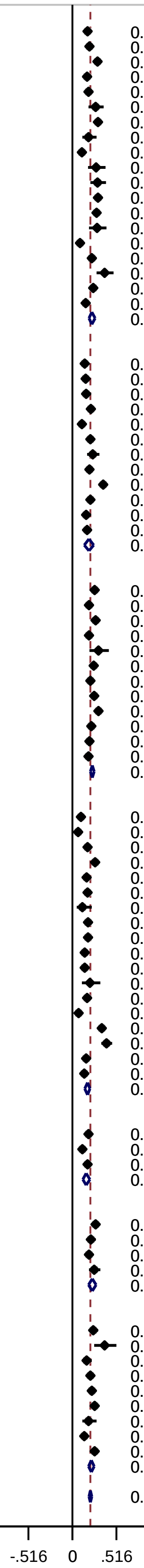

Supplementary Figure S9: Maternal GBS prevalence in Africa: adjusted

Country	Year	Author	No of women tested	Number of women positive (real or adjusted)	ES (95% CI)	% Weight
Southern Africa						
South Africa	2009	Cutland	3964	1180	0.30 (0.28, 0.31)	2.05
South Africa	2014	Kwatra	521	148	0.28 (0.25, 0.32)	1.98
South Africa		Cutland	4649	1153	0.25 (0.24, 0.26)	2.05
South Africa	2015	Bolukaoto	413	128	0.31 (0.27, 0.36)	1.96
South Africa	2011	Madzivhandila	2561	783	0.31 (0.29, 0.32)	2.04
South Africa	2016	Monyama	413	128	0.31 (0.27, 0.36)	1.96
South Africa	2015	Chukwu	413	128	0.31 (0.27, 0.36)	1.96
South Africa	2016	Dangor	284	72	0.25 (0.20, 0.31)	1.93
Subtotal (I-squared = 84.3%, p = 0.000)					0.29 (0.27, 0.31)	15.91
.						
Eastern Africa						
Zimbabwe	2010	Mavenyengwa	672	142	0.21 (0.18, 0.24)	2.01
Zimbabwe	2006	Mavenyengwa	300	138	0.46 (0.40, 0.52)	1.90
Zimbabwe	2006	Mavenyengwa	100	60	0.60 (0.50, 0.70)	1.65
Zimbabwe	2004	Whitney	210	35	0.17 (0.12, 0.22)	1.93
Zimbabwe	2000	Moyo	206	65	0.32 (0.25, 0.38)	1.86
Malawi	2011	Gray	1857	390	0.21 (0.19, 0.23)	2.04
Malawi	2005	Dzowela	97	24	0.25 (0.17, 0.35)	1.71
Kenya	2016	Seale	526	47	0.09 (0.07, 0.12)	2.03
Kenya		Seale	5470	608	0.11 (0.10, 0.12)	2.05
Kenya		Seale	1971	279	0.14 (0.13, 0.16)	2.04
Tanzania	2009	Joachim	300	69	0.23 (0.18, 0.28)	1.94
Ethiopia	2014	Woldu	300	22	0.07 (0.05, 0.11)	2.01
Ethiopia	2015	Alemseged	139	27	0.19 (0.13, 0.27)	1.84
Ethiopia	2012	Musa	139	29	0.21 (0.14, 0.29)	1.83
Ethiopia	2015	Gebremeskel	150	17	0.11 (0.07, 0.18)	1.92
Ethiopia	2016	Mengist	126	24	0.19 (0.13, 0.27)	1.82
Mozambique	2008	De Steenwinkel	113	2	0.02 (0.00, 0.06)	2.01
Mozambique	2016	Madrid	320	68	0.21 (0.17, 0.26)	1.95
Ethiopia	2014	Woldu	300	22	0.07 (0.05, 0.11)	2.01
Subtotal (I-squared = 96.3%, p = 0.000)					0.19 (0.16, 0.23)	36.56
.						
Western Africa						
Gambia	1994	Suara	136	30	0.22 (0.15, 0.30)	1.82
Gambia	2016	Le Doare	750	253	0.34 (0.30, 0.37)	2.00
Nigeria	1983	Dawodu	225	62	0.28 (0.22, 0.34)	1.89
Nigeria	2012	Onipede	150	28	0.19 (0.13, 0.26)	1.86
Nigeria	1986	Olanisebe	500	11	0.02 (0.01, 0.04)	2.05
Nigeria	1980	Onile	388	100	0.26 (0.21, 0.30)	1.96
Nigeria	2016	Onwuezobe	150	2	0.01 (0.00, 0.05)	2.03
Nigeria	2006	Nwachukwu	200	27	0.14 (0.09, 0.19)	1.94
Senegal	2009	Brochet	797	226	0.28 (0.25, 0.32)	2.01
Ghana	2015	Vinnemeier	103	24	0.23 (0.16, 0.33)	1.74
Ghana	2015	Vinnemeier	399	73	0.18 (0.15, 0.22)	1.98
Togo	2005	Balaka	306	27	0.09 (0.06, 0.13)	2.00
Togo	1991	David-Prince	106	4	0.04 (0.01, 0.09)	1.97
Ghana	2011	Enweronu-Laryea	100	19	0.19 (0.12, 0.28)	1.77
Subtotal (I-squared = 97.9%, p = 0.000)					0.17 (0.11, 0.24)	27.02
.						
Middle Africa						
DRC	2014	Mitima	509	146	0.29 (0.25, 0.33)	1.98
Gabon	2014	Capan-Melser	549	106	0.19 (0.16, 0.23)	2.00
Subtotal (I-squared = 91.8%, p = 0.000)					0.24 (0.15, 0.33)	3.98
.						
Northern Africa						
Tunisia	2005	Ferjani	300	39	0.13 (0.09, 0.17)	1.98
Tunisia	2008	Ben Hamouda	207	57	0.28 (0.22, 0.34)	1.87
Tunisia	2007	Jerbi	294	38	0.13 (0.09, 0.17)	1.98
Egypt	2009	Shabayek	150	54	0.36 (0.28, 0.44)	1.78
Egypt	2009	Abdelmoaty	150	39	0.26 (0.19, 0.34)	1.82
Egypt	2017	Sadaka	200	53	0.26 (0.21, 0.33)	1.87
Morocco	2010	Mahmoud	240	56	0.23 (0.18, 0.29)	1.91
Morocco	1983	Benbachir	35	7	0.20 (0.08, 0.37)	1.37
Morocco		Bassat	347	82	0.24 (0.19, 0.28)	1.95
Subtotal (I-squared = 85.5%, p = 0.000)					0.23 (0.18, 0.28)	16.54
.						
Overall (I-squared = 97.9%, p = 0.000)					0.21 (0.18, 0.24)	100.00

NOTE: Weights are from random effects analysis

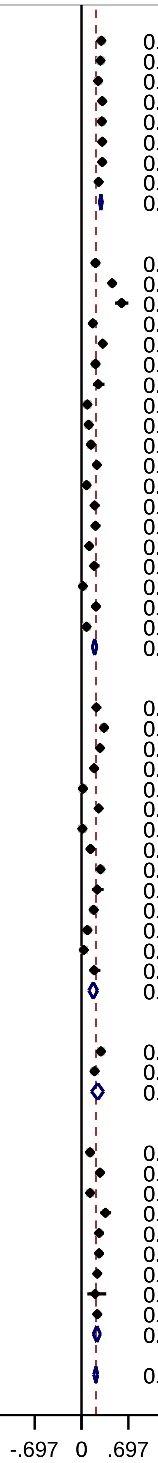

Supplementary Figure S10: Maternal GBS prevalence in Latin America and the Caribbean: adjusted `

Country	Year	Author	No of women tested	Number of women positive (real or adjusted)	ES (95% CI)	% Weight
South America						
Brazil	2010	Castellano-Filho	221	21	0.10 (0.06, 0.14)	2.64
Brazil	2006	Zusman	598	152	0.25 (0.22, 0.29)	2.67
Brazil	2011	Pogere	273	59	0.22 (0.17, 0.27)	2.57
Brazil	1982	Benchetrit	86	22	0.26 (0.17, 0.36)	2.13
Brazil	2012	Giraldo	45	7	0.16 (0.06, 0.29)	1.95
Brazil	2011	Ronchetti	405	103	0.25 (0.21, 0.30)	2.62
Brazil	2011	Linhares	213	21	0.10 (0.06, 0.15)	2.63
Brazil	2010	Pires	198	30	0.15 (0.10, 0.21)	2.55
Brazil	2008	Lima Dos Reis	201	41	0.20 (0.15, 0.27)	2.51
Brazil	2010	Marconi	405	103	0.25 (0.21, 0.30)	2.62
Brazil	2005	Lermontov	167	32	0.19 (0.13, 0.26)	2.47
Brazil	2013	Kiss	105	16	0.15 (0.09, 0.24)	2.37
Brazil	2008	Costa	201	41	0.20 (0.15, 0.27)	2.51
Brazil	2007	Simoes	316	46	0.15 (0.11, 0.19)	2.64
Brazil	2007	Beitune	101	20	0.20 (0.13, 0.29)	2.28
Brazil	2006	Beitune	106	15	0.14 (0.08, 0.22)	2.39
Brazil	1981	Benchetrit	42	11	0.26 (0.14, 0.42)	1.69
Brazil	2008	Chaves	102	25	0.25 (0.17, 0.34)	2.23
Brazil	2015	Nunes	144	87	0.60 (0.52, 0.68)	2.28
Brazil	2015	Siqueira	411	58	0.14 (0.11, 0.18)	2.67
Argentina	2011	Ronchi	324	21	0.06 (0.04, 0.10)	2.71
Argentina	2013	Oviedo	3125	293	0.09 (0.08, 0.10)	2.77
Argentina	2008	Quiroga	1105	84	0.08 (0.06, 0.09)	2.76
Argentina	2005	Larcher	1228	17	0.01 (0.01, 0.02)	2.78
Argentina	2005	Di Bartolomeo	1203	113	0.09 (0.08, 0.11)	2.75
Argentina	2001	Toresani	531	24	0.05 (0.03, 0.07)	2.75
Chile	2014	Abarzua	1181	167	0.14 (0.12, 0.16)	2.74
Peru	2004	Tamariz Ortiz	238	26	0.11 (0.07, 0.16)	2.63
Uruguay	2009	Laufer	300	52	0.17 (0.13, 0.22)	2.61
Venezuela	2007	Amesty	100	18	0.18 (0.11, 0.27)	2.31
Venezuela	1993	Riera	171	79	0.46 (0.39, 0.54)	2.33
Venezuela	1979	Pina-Carruyo	122	48	0.39 (0.31, 0.49)	2.20
Colombia	2014	Ceballos	182	32	0.18 (0.12, 0.24)	2.51
Paraguay	2013	Ortiz	203	48	0.24 (0.18, 0.30)	2.48
Subtotal (I-squared = 97.1%, p = 0.000)					0.18 (0.15, 0.21)	84.75
Central America						
Mexico	1989	Solorzano-Santos	340	49	0.14 (0.11, 0.19)	2.65
Guatemala	2016	Asturius	990	155	0.16 (0.13, 0.18)	2.73
Subtotal (I-squared = 0.0%, p = 0.589)					0.15 (0.13, 0.17)	5.38
Caribbean						
Dominican Republic	2006	Fernandez	207	90	0.43 (0.37, 0.51)	2.40
Trinidad	2004	Orrett	405	130	0.32 (0.28, 0.37)	2.60
Trinidad	2003	Orrett	201	66	0.33 (0.26, 0.40)	2.43
Trinidad	1994	Orrett	204	64	0.31 (0.25, 0.38)	2.44
Subtotal (I-squared = 64.7%, p = 0.037)					0.35 (0.30, 0.40)	9.87
Overall (I-squared = 97.4%, p = 0.000)					0.20 (0.17, 0.23)	100.00

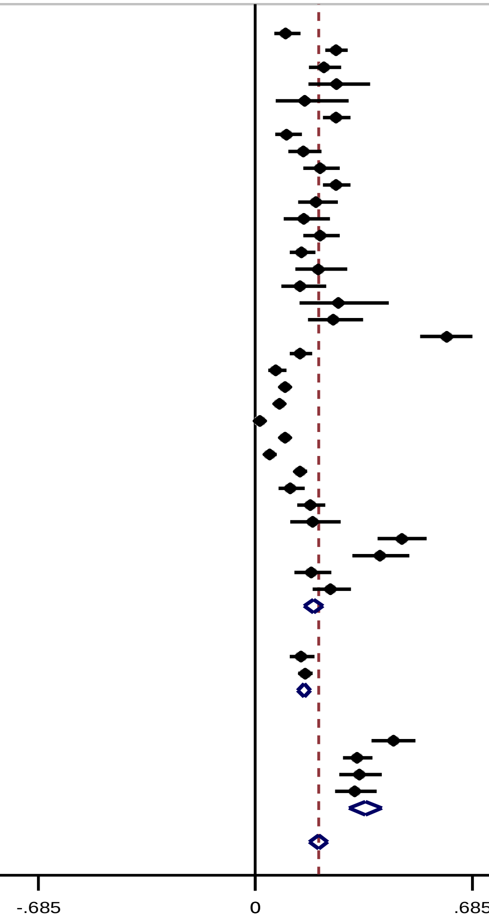

NOTE: Weights are from random effects analysis

Supplementary Figure S11: Maternal GBS prevalence in Asia: adjusted`

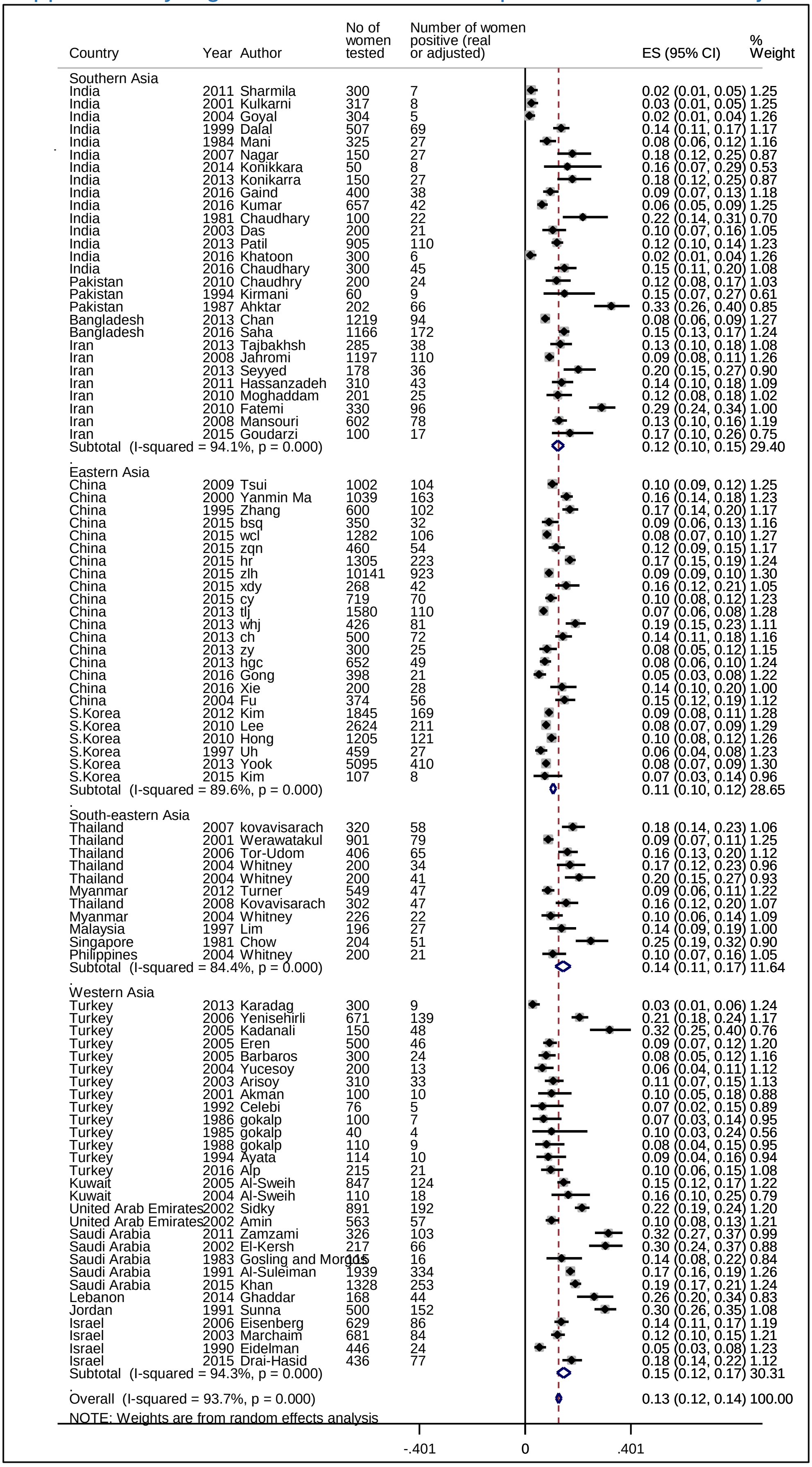

Supplementary Figure S12: Maternal GBS colonization serotypes distribution worldwide

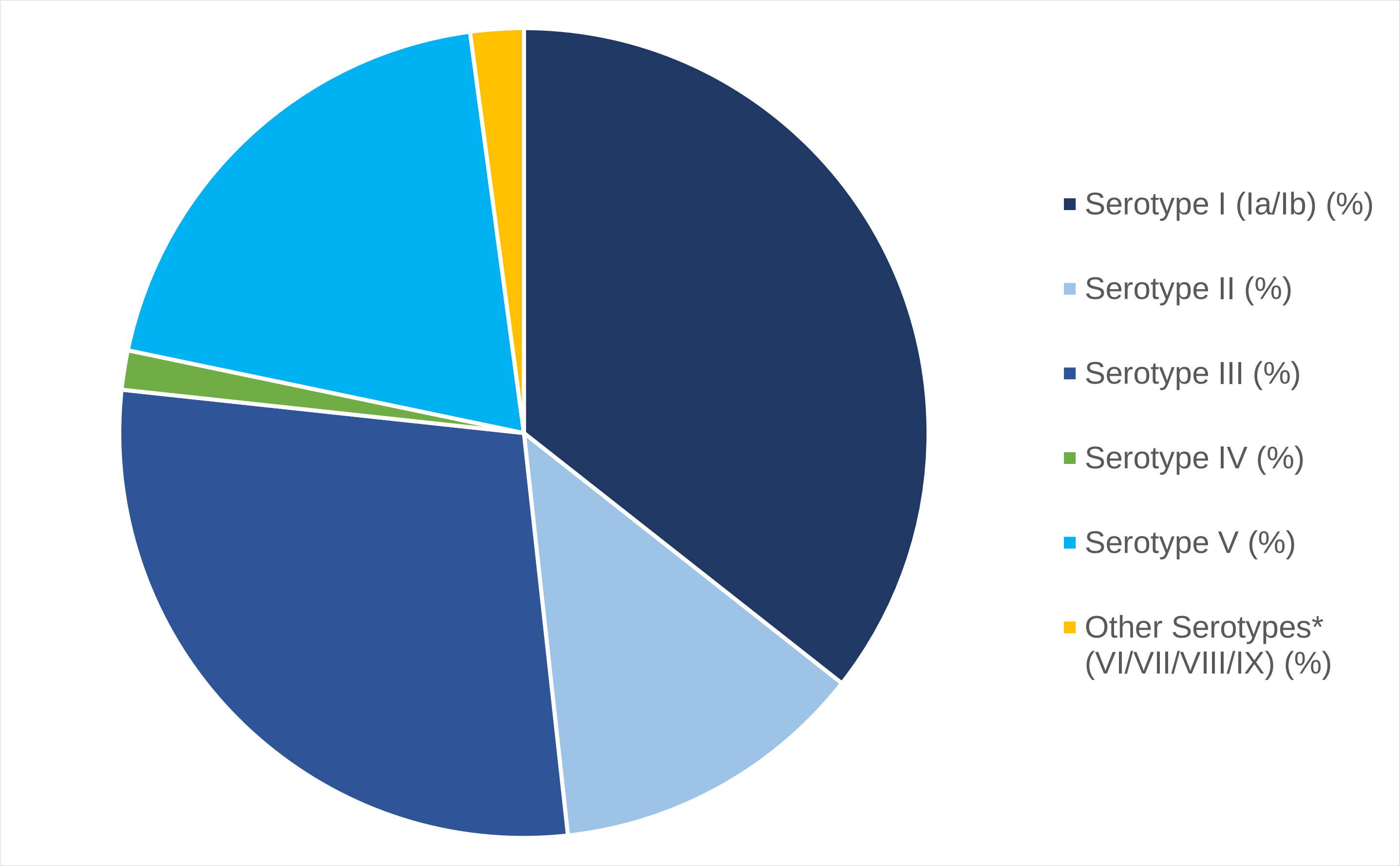

Supplementary Figure S13: GBS maternal colonization serotypes distribution in developed regions

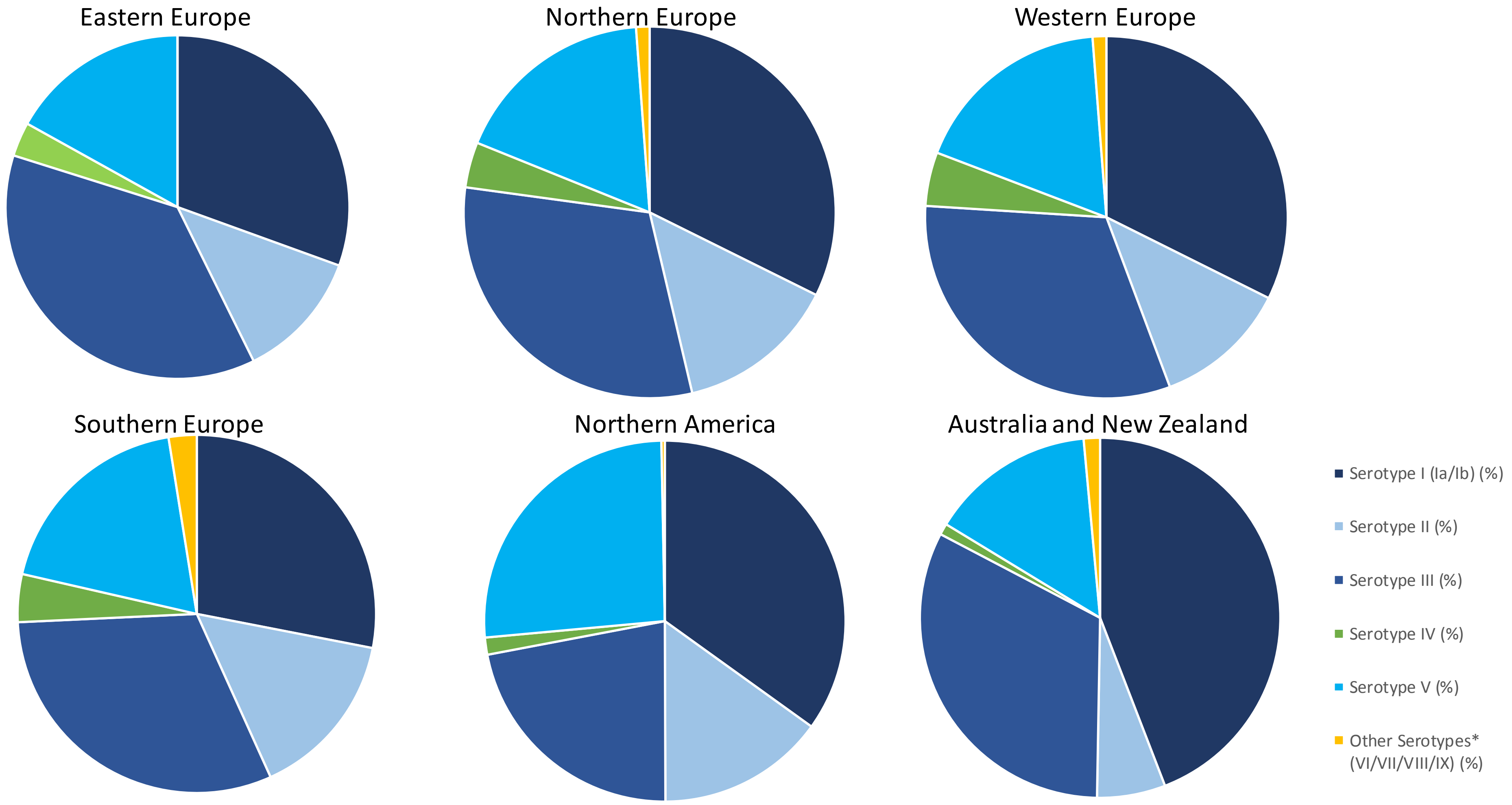

Supplementary Figure S14: GBS maternal colonization serotypes distribution in Africa

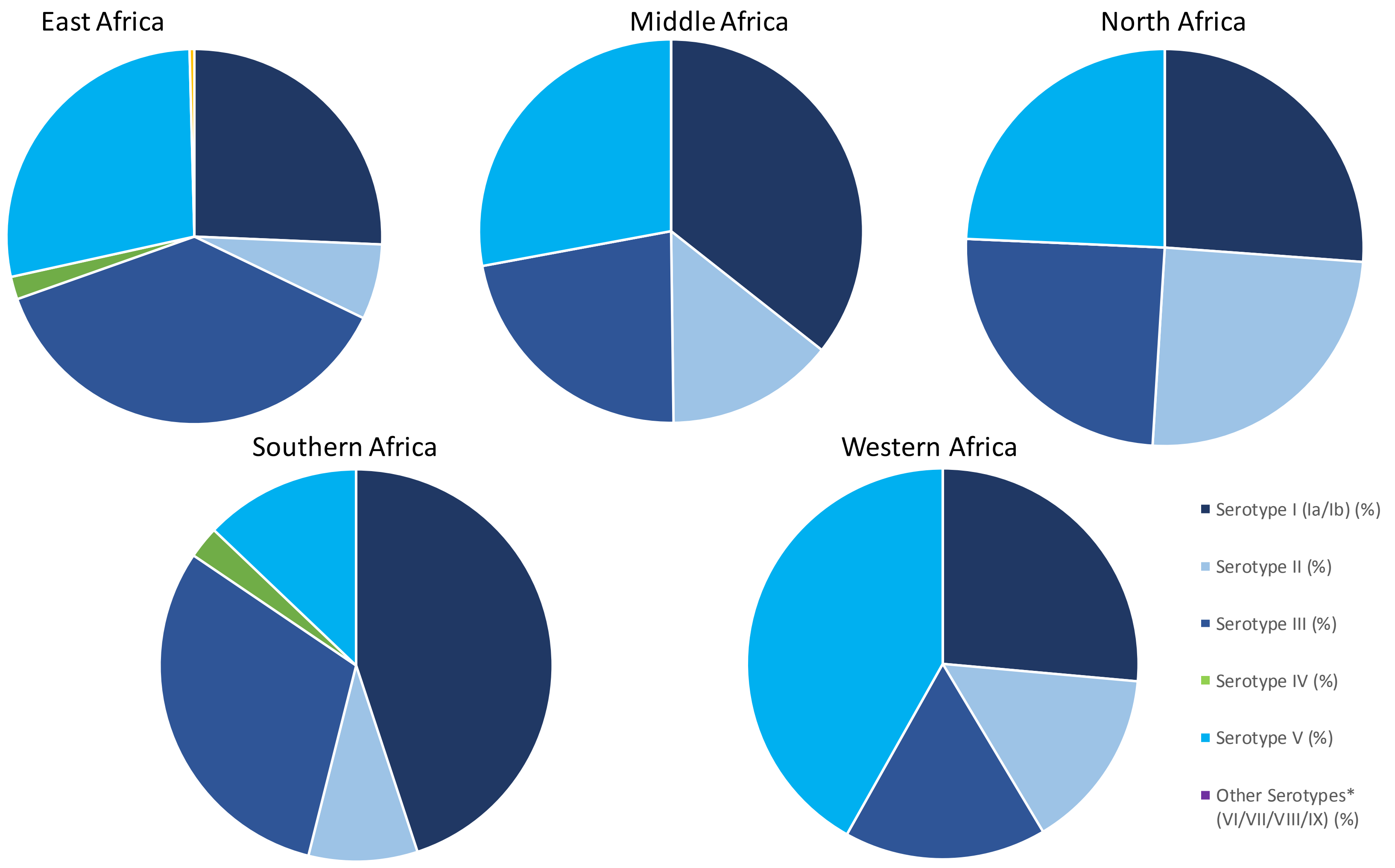

Supplementary Figure S15: GBS maternal colonization serotypes distribution in South and Central America

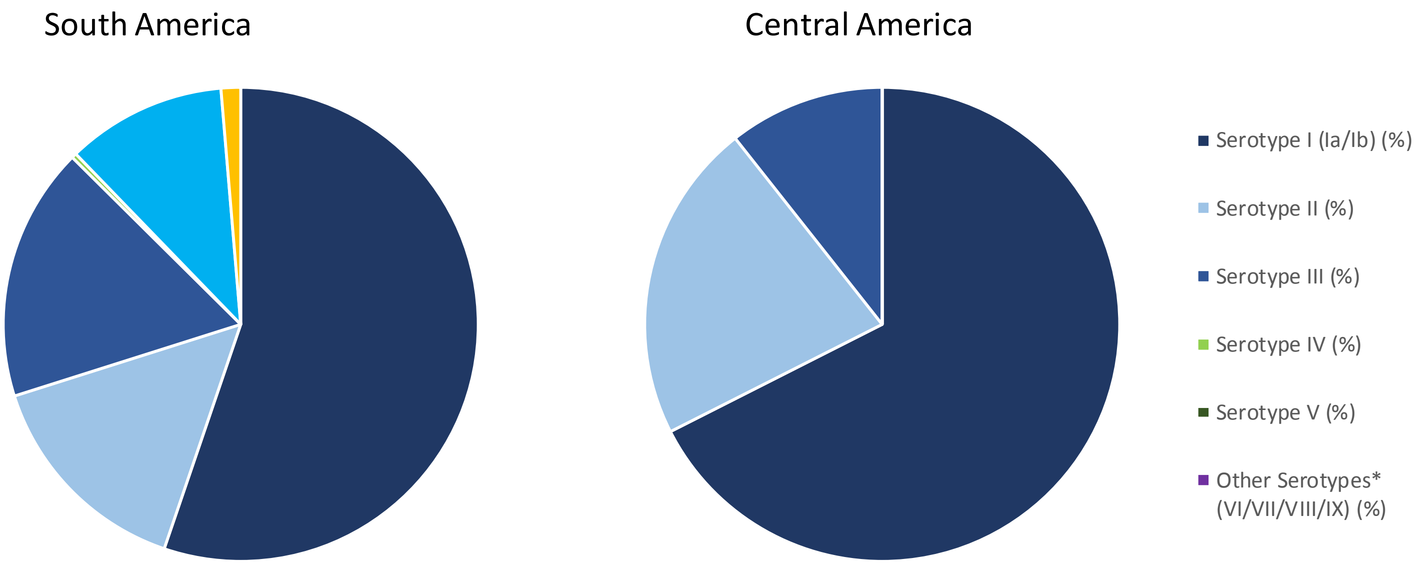

Supplementary Figure S16: GBS maternal colonization serotypes distribution in Asia

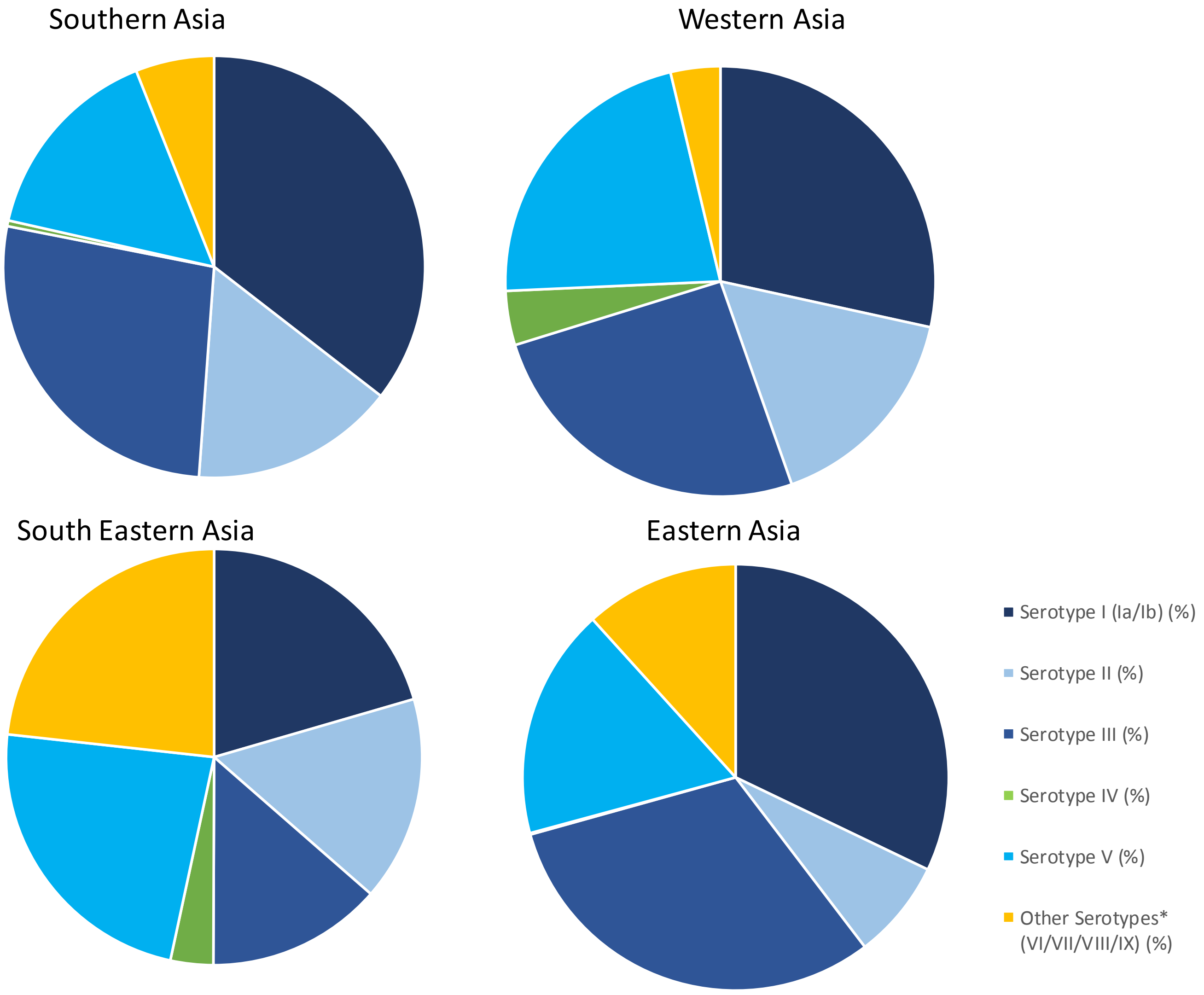

References

1. Gilbert GL, Hewitt MC, Turner CM, Leeder SR. Epidemiology and predictive values of risk factors for neonatal group B streptococcal sepsis. The Australian & New Zealand journal of obstetrics & gynaecology **2002**; 42(5): 497-503.
2. Hiller JE, McDonald HM, Darbyshire P, Crowther CA. Antenatal screening for group B Streptococcus: A diagnostic cohort study. Available at: http://www.biomedcentral.com/1471-2393/5/12

http://ovidsp.ovid.com/ovidweb.cgi?T=JS&PAGE=reference&D=emed7&NEWS=N&AN=2005466772. Accessed (Hiller) Department of Public Health, University of Adelaide, Adelaide, SA 5005, Australia.
3. Taylor JK, Hall RW, Dupre AR. The incidence of group B streptococcus in the vaginal tracts of pregnant women in central Alabama. Clinical laboratory science : journal of the American Society for Medical Technology **2002**; 15(1): 16-7.
4. Grimwood K, Stone PR, Gosling IA, et al. Late antenatal carriage of group B Streptococcus by New Zealand women. Australian and New Zealand Journal of Obstetrics and Gynaecology **2002**; 42(2): 182-6.
5. Fernandes J. SJ, Feris J.M., Gomez E., Serulle Y., Demorizi J., Rivera L., Rivera-Almodovar E., Mercedes H., Perez-Then E. Prevalence of Group B Streptococcus infections in dominican pregnant women. Rev Panam Infectol **2006**; 8(1): 26-32.
6. Orrett FA. Antimicrobial susceptibility patterns of group B Streptococci isolated from pregnant women in Trinidad, West Indies. Saudi Medical Journal **2004**; 25(11): 1764-5.
7. Orrett FA. Colonization with group B streptococci in pregnancy and outcome of infected neonates in Trinidad. Pediatrics International **2003**; 45(3): 319-23.
8. Orrett FA, Olagundoye V. Prevalence of Group B streptococcal colonization in pregnant third trimester women in Trinidad. Journal of Hospital Infection **1994**; 27(1): 43-8.
9. Cruz AA, Peraza GT, Caballero RL. Vaginal and rectal streptococcus agalactiae colonization in pregnant women from melena del sur municipality in cuba. Revista Cubana de Medicina Tropical **2014**; 66(3): 415-23.
10. Gonzalez Pedraza Aviles A, Ortiz Zaragoza MC, Mota Vazquez R. Serotypes and antimicrobial susceptibility of group B Streptococcus from pregnant women in Mexico. Rev Latinoam Microbiol **2002**; 44.
11. Gonzalez PA OZ, Madrigal de Leon HG, Corzo CMT, Flores HP. . Colonizacion por streptococcus grupo b en mujeres embarazadas de un centro de atencion primaria de la Ciudad de Mexico Mrch Med Fam **2004**; 6: 44-7.
12. Solorzano-Santos F E-AG, Conde-Gonzalez CJ, Calderon-Jaimes E, Arredondo-Garcia JL, Beltran Zuniga M. CervicoVaginal Infection with Group B streptococci among Pregnant Mexican Women The Journal of Infectious Diseases **1989**; 159(5): 1003-4.
13. de Lourdes Collado M, Kretschmer RR, Becker I, Guzman A, Gallardo L, Lepe CM. Colonization of Mexican pregnant women with group B streptococcus. The Journal of infectious diseases **1981**; 143(1): 134.
14. Ocampo-Torres M, Sánchez-Pérez HJ, Nazar-Beutelspacher A, Castro-Ramírez AE, Cordero-Ocampo B. Factors associated with group B Streptococcus colonization in pregnant women of Los Altos, Chiapas, Mexico. Salud Publica de Mexico **2000**; 42(5): 413-21.
15. Mavenyengwa RT, Afset JE, Schei B, et al. Group B Streptococcus colonization during pregnancy and maternal-fetal transmission in Zimbabwe. Acta Obstetricia et Gynecologica Scandinavica **2010**; 89(2): 250-5.

16. Mavenyengwa RT, Masunga P, Meque E, et al. Streptococcus agalactiae (group B streptococcus (GBS)) colonisation and persistence, in pregnancy; a comparison of two diverse communities (rural and urban). The Central African journal of medicine **2006**; 52(3-4): 38-43.
17. Whitney CG, Daly S, Limpongsanurak S, et al. The international infections in pregnancy study: Group B streptococcal colonization in pregnant women. Journal of Maternal-Fetal and Neonatal Medicine **2004**; 15(4): 267-74.
18. Moyo SR, Mudzori J, Tswana SA, Maeland JA. Prevalence, capsular type distribution, anthropometric and obstetric factors of group B Streptococcus (Streptococcus agalactiae) colonization in pregnancy. The Central African journal of medicine **2000**; 46(5): 115-20.
19. Mason PR, Gwanzura L, Latif AS, Ray S, Van De Wijgert J, Katzenstein DA. Antimicrobial susceptibility patterns amongst group B streptococci from women in Harare, Zimbabwe. International Journal of Antimicrobial Agents **1996**; 7(1): 29-32.
20. Mason PR, Katzenstein DA, Chimbira TH, Mtimavalye L. Microbial flora of the lower genital tract of women in labour at Harare Maternity Hospital. The Puerperal Sepsis Study Group. Central African Journal of Medicine **1989**; 35(3): 337-44.
21. Gray KJ, Kafulafula G, Matemba M, Kamdolozi M, Membe G, French N. Group B streptococcus and HIV infection in pregnant women, Malawi, 2008-2010. Available at: http://wwwnc.cdc.gov/eid/article/17/10/pdfs/10-2008.pdf

http://ovidsp.ovid.com/ovidweb.cgi?T=JS&PAGE=reference&D=emed13&NEWS=N&AN=2011540366. Accessed (Gray, Kafulafula, Kamdolozi, Membe) College of Medicine, Blantyre, Malawi.
22. Dzowela TK, O.O. and Igbigbi, A. . Prevalence of Group B Streptococcus colonization in antenatal women at the Queen Elizabeth Central Hospital, Blantyre - A preliminary study. Malawi Medical Journal **2005** 17(3): 97-9.
23. Seale AC, Koech AC, Sheppard AE, et al. Maternal colonisation with Streptococcus agalactiae, and associated stillbirth and neonatal disease in coastal Kenya. Nature microbiology **2016**; 1(7): 16067-.
24. Ernest AI, Ng'Walida N, Ndaboine E, Massinde A, Kihunrwa A, Mshana S. Maternal vaginorectal colonization by Group B Streptococcus and Listeria monocytogenes and its risk factors among pregnant women attending tertiary hospital in Mwanza, Tanzania. Tanzania Journal of Health Research **2015**; 17(2).
25. Joachim A, Matee MI, Massawe FA, Lyamuya EF. Maternal and neonatal colonisation of group B streptococcus at Muhimbili National Hospital in Dar es Salaam, Tanzania: Prevalence, risk factors and antimicrobial resistance. BMC Public Health **2009**; 9.
26. Woldu ZL, Teklehaimanot TG, Waji ST, Gebremariam MY. The prevalence of Group B Streptococus recto-vaginal colonization and antimicrobial susceptibility pattern in pregnant mothers at two hospitals of Addis Ababa, Ethiopia. Reproductive health **2014**; 11: 80.
27. Alemseged G, Niguse S, Hailekiros H, Abdulkadir M, Saravanan M, Asmelash T. Isolation and anti-microbial susceptibility pattern of group B Streptococcus among pregnant women attending antenatal clinics in Ayder Referral Hospital and Mekelle Health Center, Mekelle, Northern Ethiopia Health Services Research. BMC Research Notes **2015**; 8(1).
28. Mohammed M, Asrat D, Woldeamanuel Y, Demissie A. Prevalence of group B Streptococcus colonization among pregnant women attending antenatal clinic of Hawassa Health Center, Hawassa, Ethiopia. Ethiopian Journal of Health Development **2012**; 26(1): 36-42.
29. Gebremeskel T.K.; Zeleke TA, Mihret A., Tikue M.D. Prevalence and Antibiotic Susceptibility Pattern of Streptococcus agalactiae Among Pregnant Women at Adigrat Zonal Hospital and Adigrat Health Center, Tigray. Journal of Gynaecology and Obstetrics **2015**; 3(2): 29-35.
30. Mengist A, Kannan H, Abdissa A. Prevalence and antimicrobial susceptibility pattern of anorectal and vaginal group B Streptococci isolates among pregnant women in Jimma, Ethiopia. BMC Res Notes **2016**; 9: 351.

31. De Steenwinkel FDO, Tak HV, Muller AE, Nouwen JL, Oostvogel PM, Mocumbi SM. Low carriage rate of group B streptococcus in pregnant women in Maputo, Mozambique. Tropical Medicine and International Health **2008**; 13(3): 427-9.
32. Yim SF, Lyon DJ, Chung TK, Haines CJ. A prospective study of the microbiological environment of the genitourinary tract in Hong Kong Chinese women during pregnancy. The Australian & New Zealand journal of obstetrics & gynaecology **1995**; 35(2): 178-81.
33. Tsui MHY, Ip M, Ng PC, Sahota DS, Leung TN, Lau TK. Change in prevalence of group B Streptococcus maternal colonisation in Hong Kong. Hong Kong Medical Journal **2009**; 15(6): 414-9.
34. Wang P, Tong JJ, Ma XH, et al. Serotypes, antibiotic susceptibilities, and multi-locus sequence type profiles of Streptococcus agalactiae isolates circulating in Beijing, China. Available at: http://www.plosone.org/article/fetchObject.action?uri=info:doi/10.1371/journal.pone.0120035&representation=PDF

http://ovidsp.ovid.com/ovidweb.cgi?T=JS&PAGE=reference&D=emed13&NEWS=N&AN=2015838651. Accessed (Wang, Tong, Shi, Yu, Yao, Yang) Key Laboratory of Major Diseases in Children and National Key Discipline of Pediatrics, Beijing Children's Hospital, Capital Medical University, Beijing 100045, China.
35. Lu B, Li D, Cui Y, Sui W, Huang L, Lu X. Epidemiology of Group B streptococcus isolated from pregnant women in Beijing, China. Clinical Microbiology and Infection **2014**; 20(6): O370-O3.
36. Ma Y, Wu L, Huang X. Study on perinatal group B Streptococcus carriers and the maternal and neonatal outcome. Zhonghua fu chan ke za zhi **2000**; 35(1): 32-5.
37. Zhang JH, Yuan L, Yang YH. Perinatal colonization of group B streptococcus: a study in 600 cases in Beijing Tiantan Hospital. Zhonghua liu xing bing xue za zhi = Zhonghua liuxingbingxue zazhi **1995**; 16(1): 36-9.
38. Liang ST, Lau SP, Chan SH, Fok TF, Murai T, Kaneko Y. Perinatal colonization of group B streptococcus--an epidemiological study in a Chinese population. The Australian & New Zealand journal of obstetrics & gynaecology **1986**; 26(2): 138-41.
39. bsq. 孕妇生殖道B族链球菌感染对母婴预后影响的临床研究 Clinical study of impact of reproductive tract group B Streptococcus infections on prognosis of mothers and infants. Chin J Nosocomiol **2015**.
40. gxm. B族溶血性链球菌感染的孕妇经产时抗生素预防治疗后临床效果观察 Clinical study of impact of Intravenous Intrapartum Antibiotic Prophylaxis to group B Streptococcus infections. Chin J Med Drug Appl **2015**.
41. xax. 围产期孕妇B群链球菌感染的检测及耐药性分析 Group B Streptococcus Infection Detection and antimicrobial resistance analysis of Perinatal Pregnant Women Chinese Journal of Practical Pediatrics **2015**.
42. wcl. 不同孕妇人群B族链球菌携带率差异分析 Carrier Rate Difference Rate Analysis on Group B Streptococcus of Different Group of Pregnant Women. 中国初级卫生保健 **2015**.
43. zqn. 孕妇妊娠晚期B族链球菌感染对妊娠结局及新生儿的影响 Impact of GBS Infection on Pregnancy Outcome and infant in Perinatal Pregnant Women. Journal of Qiqihar University of Medicine **2015**.

44. hr. **孕35~37周**妇女无乳链球菌携带状况及耐药性分析 Carrier rate and antimicrobial resistance analysis of Group B Streptococcus of 35-37w Pregnant Women China J Clin Obstet Gynecol **2015**.
45. zlh. **广**东东莞地区2009-2014**年**围产期孕妇 B 群链球菌的 **分离与耐**药性分析 Prevalence and antibiotic resistance of Group B Streptococcus isolated from perinatal pregnant women during the period from 2009 to 2014 in Dongguan, Guangdong Province. Chin J Infect Chemother **2015**.
46. wj. **徐州地区妊娠晚期**妇女感染B**群**链球菌的筛检情况及药物敏感性分析 Screening for group B streptococcal infection in late pregnant women in Xuzhou area and its drug sensitivity analysis. **国**际检验医学杂志 **2015**.
47. xdy. **妊娠晚期型溶血性**链球菌感染对母婴结局的影响 Influcece of B type hemolytic streptococcus at later period of pregnancy for maternal-infant outcome. **世界最新医学信息文摘 2015**.
48. cy. **妊娠**35~37**周孕**妇B**族**链球菌带菌与耐药性分析 Study on perinatal group B Streptococcus carriers in late pregnancy and analysis of drug resistance. J Trop Med **2015**.
49. tlj. 现代诊断与治疗 Influence of group B streptococcus infection on pregnancy outcome and infants. Mod Diagn Treat **2013** 24(19).
50. Haijun WU, Lei L.V., Liuan S., Qingyu X O. **群**链球菌在孕妇及新生儿中的带菌调查及耐药性研究. Journal of Chongqing Medical University **2013**; 38(10).
51. ch. 围产期 B 群链球菌感染及相关研究Analysis of perinatal group B streptococcus infection and related conditions. Int J Lab Med **2013**.
52. sdh. **妊娠**35-37**周孕**妇 B 族链球菌带菌与妊娠结局 Study on perinatal group B Streptococcus carriers in late pregnancy and the pregnancy outcome. Chin J Clin Obstet Gynecol **2013**.
53. zy. **妊娠晚期孕**妇 B **族溶血性**链球菌感染对母儿的影响 Impact of group B streptococcus infection on late-pregnancy women and their neonates. J Med Postgra **2013**.
54. wx. **孕**妇无乳链球菌的感染及药物敏感性分析 Infection and antibiotic susceptibility analysis of Group B Streptococcus of Pregnant Women. 实用医学杂志 **2013**.
55. hyj. **孕晚期**妇女 B **族**链球菌 PCR 检测结果分析Analysis of group B streptococcus detection results by real-time polymerase chain reaction in 445 late pregnants. Jiangxi Medical Journal **2013**.
56. hgc. **桂林地区孕晚期孕**妇 B 族链球菌检测及药敏分析 Detection and antimicrobial resistance analysis of Group B Streptococcus of Pregnant Women in Guiling. Int J Lab Med **2013**.
57. Xie Y, Yang J, Zhao P, Jia H, Wang Q. Occurrence and detection method evaluation of group B streptococcus from prenatal vaginal specimen in Northwest China. Diagnostic pathology **2016**; 11: 8.

58. Yan JJ, Gong M, Zhang J, Zhu CB. [The relationship between group B streptococcus genital infection and premature rupture of membrane]. Zhonghua yi xue za zhi **2016**; 96(23): 1847-9.
59. Yang MJ, Sun PL, Wen KC, et al. Prevalence of maternal group B streptococcus colonization and vertical transmission in low-risk women in a single institute. Journal of the Chinese Medical Association **2012**; 75(1): 25-8.
60. Li YP, Kuok CM, Lin SY, Hsieh WS, Shyu MK. Group B streptococcus antimicrobial resistance in neonates born to group B streptococcus-colonized mothers: Single-center survey. Journal of Obstetrics and Gynaecology Research **2016**; 42(11): 1471-5.
61. Fu JC, Lin DP, Yang CF, Huang WJ, Huang CS. Antibiotic suceptibility pattern of anovaginal isolates of Streptococcus agalactiae from pregnant women in their late third trimester. Kaohsiung Journal of Medical Sciences **2004**; 20(7): 330-3.
62. Kim MY, Ki M, Yang JH, et al. Effectiveness evaluation of group B streptococcus screening in pregnant women for prevention of neonatal infection. Available at: http://ovidsp.ovid.com/ovidweb.cgi?T=JS&PAGE=reference&D=emed10&NEWS=N&AN=70906702. Accessed (Kim, Yang, An, Han, Yook) Department of Obstetrics, Cheil Hospital and Women's Healthcare Center, Kwandong University College of Medicine, Seoul, Korea, Seoul, South Korea.
63. Lee BK, Song YR, Kim MY, et al. Epidemiology of group B streptococcus in Korean pregnant women. Epidemiology and infection **2010**; 138(2): 292-8.
64. Hong JS, Choi CW, Park KU, et al. Genital group B streptococcus carrier rate and serotype distribution in Korean pregnant women: Implications for group B streptococcal disease in Korean neonates. Journal of Perinatal Medicine **2010**; 38(4): 373-7.
65. Uh Y, Jang IH, Yoon KJ, Lee CH, Kwon JY, Kim MC. Colonization rates and serotypes of group B streptococci isolated from pregnant women in a Korean tertiary hospital. European Journal of Clinical Microbiology and Infectious Diseases **1997**; 16(10): 753-6.
66. Yook JH, Kim MY, Kim EJ, et al. Risk factors associated with group B streptococcus resistant to clindamycin and erythromycin in pregnant Korean women. Infection and Chemotherapy **2013**; 45(3): 299-307.
67. Kim DH, Kim YN, Jeong EJ, et al. The prevalence of group b streptococcus (GBS) colonization in Korean pregnant women using selective culture media. International Journal of Gynecology and Obstetrics **2015**; 131: E473.
68. Terakubo S, Ichiman Y, Takemura H, Yamamoto H, Shimada J, Nakashima H. Serotypes and antibody levels of group B streptococci in pregnant women. Kansenshogaku zasshi The Journal of the Japanese Association for Infectious Diseases **2003**; 77(3): 121-6.
69. Matsubara K, Katayama K, Baba K, Nigami H, Harigaya H, Sugiyama M. Seroepidemiologic studies of serotype VIII group B Streptococcus in Japan. Journal of Infectious Diseases **2002**; 186(6): 855-8.
70. Kubota T, Nojima M, Itoh S. Vaginal bacterial flora of pregnant women colonized with group B streptococcus. Journal of Infection and Chemotherapy **2002**; 8(4): 326-30.
71. Morozumi M, Chiba N, Igarashi Y, et al. Direct identification of Streptococcus agalactiae and capsular type by real-time PCR in vaginal swabs from pregnant women. Journal of infection and chemotherapy : official journal of the Japan Society of Chemotherapy **2015**; 21(1): 34-8.
72. Losonczi L, Emodi S. Screening of group B streptococcus in pregnant women and antibiotic prophylaxis during labour. Magyar Noorvosok Lapja **2002**; 65(2): 87-91.
73. Abrok M, Arcson T, Lazar A, Urban E, Deak J. Combination of selective enrichment and MALDI-TOF MS for rapid detection of Streptococcus agalactiae colonisation of pregnant women. Available at: www.elsevier.com/locate/jmicmeth

http://ovidsp.ovid.com/ovidweb.cgi?T=JS&PAGE=reference&D=emed13&NEWS=N&AN=2015003942. Accessed (Abrok, Arcson, Lazar, Urban, Deak) Institute of Clinical Microbiology, University of Szeged, Szeged, Hungary.

74. Perebendyuk TV. Colonization by Group B Streptococcus of the Urogenital and Rectal Tracts of Pregnant Women with Cured Infertility by Applying In Vitro Fertilization. 2013 **2013**; (5.48): 5.
75. Kovachev E, Markova V, Bozhkova K, Popova A, Tsvetkova S, Cherneva S. [Is there a correlation between the vaginal colonization with group B streptococci and premature deliveries?]. Ima li zavisimost mezhdu vaginalnata kolonizatsiia sus streptokoki ot grupa B i prezhdevremennite razhdaniia? **2003**; 42 Suppl 2: 3-5.
76. Motlova J, Strakova L, Urbaskova P, Sak P, Sever T. Vaginal & rectal carriage of Streptococcus agalactiae in the Czech Republic: incidence, serotypes distribution & susceptibility to antibiotics. Indian J Med Res **2004**; 119.
77. Lucovnik M, Mandic NT, Lozar Krivec J, Kolenc U, Jeverica S. Prevalence of Streptococcus agalactiae colonisation among pregnant women in Slovenia, 2013-2014. Zdravniski Vestnik **2016**; 85(7-8): 393-400.
78. Brzychczy-Włoch M, Ochońska D, Bulanda M. Carriage of group B streptococci in pregnant women from the region of Krakow and their antibiotic resistance in the years 2008-2012. Polish Journal of Microbiology **2013**; 62(4): 427-33.
79. Romanik M, Nowosielski K, Poreba R, Sioma-Markowska U, Martiroisian G, Groborz J. Streptococcus group B serotype distribution in anovaginal isolates of women in term pregnancy. Neuro Endocrinol Lett **2014**; 35(4): 301-5.
80. Brzychczy-Włoch M, Gosiewski T, Bodaszewska-Lubas M, Adamski P, Heczko PB. Molecular characterization of capsular polysaccharides and surface protein genes in relation to genetic similarity of group B streptococci isolated from Polish pregnant women. Epidemiology and Infection **2012**; 140(2): 329-36.
81. Romanik M, Nowosielski K, Martirosian G, Poręba R, Sioma-Markowska U. Identification of pregnant women at risk of Streptococcus group B colonisation. Neuroendocrinology Letters **2011**; 32(3): 308-12.
82. Kociszewska-Najman B, Oslislo A, Szymusik I, Pietrzak B, Jabiry-Zieniewicz Z. [Intrapartum prophylaxis against group B Streptococcus infection--own experience]. Available at: http://ovidsp.ovid.com/ovidweb.cgi?T=JS&PAGE=reference&D=emed9&NEWS=N&AN=21395081. Accessed (Kociszewska-Najman) Oddzial Neonatologii, I Katedra i Klinika Poloznictwa i Ginekologii Warszawskiego Uniwersytetu Medycznego, Warszawa.
83. Łysakowska ME, Kalinka J, Bigos M, Prosniewska M, Wasiela M. Occurrence of virulence genes among S. agalactiae isolates from vagina and anus of pregnant women - A pilot study. Archives of Perinatal Medicine **2011**; 17(4): 229-34.
84. Strus M, Pawlik D, Brzychczy-Włoch M, et al. Group B streptococcus colonization of pregnant women and their children observed on obstetric and neonatal wards of the University hospital in krakow, Poland. Journal of Medical Microbiology **2009**; 58(2): 228-33.
85. Krasnianin E, Skret-Magierlo J, Witalis J, et al. The incidence of Streptococcus Group B in 100 parturient women and the transmission of pathogens to the newborn. Available at: http://ovidsp.ovid.com/ovidweb.cgi?T=JS&PAGE=reference&D=emed9&NEWS=N&AN=19507563. Accessed (Krasnianin, Skret-Magierlo, Witalis, Barnas, Kluz, Koziel, Skret) Institute of Nursing and Obstetrics, Faculty of Medicine, University of Rzeszow, Poland.
86. Brzychczy-Włoch M, Gosiewski T, Bodaszewska M, Pabian W, Bulanda M, Heczko PB. Analysis of serotypes distribution of group B streptococci origin from pregnant carriage using multiplex PCR. Medycyna doświadczalna i mikrobiologia **2009**; 61(4): 293-9.

87. Brzychczy-Włoch M, Strus M, Pawlik D, et al. [Increasing Streptococcus agalactiae colonization of pregnant women and newborns in south-eastern region of Poland]. Medycyna doświadczalna i mikrobiologia **2008**; 60(1): 5-12.
88. Elzbieta K, Joanna SM, Janusz W, et al. The incidence of Streptococcus group B in 100 parturient women and the transmission of pathogens to the newborn. Ginekologia Polska **2009**; 80(4): 285-9.
89. Kowalska B, Niemiec KT, Drejewicz H, et al. [Prevalence of group B streptococcal colonization in pregnant women and their newborns based on the results of examination of patients in the Obstetric and Gynecology Department of the National Research Institute of Mother and Child--a pilot study]. Czestosc wystepowania kolonizacji paciorkowcami beta hemolizujacymi grupy B kobiet ciezarnych i noworodkow--okreslona na podstawie badan przeprowadzonych u pacjentek Polikliniki i Kliniki Poloznictwa i Ginekologii Instytutu Matki i Dziecka--badanie pilotazowe **2003**; 74(10): 1223-7.
90. Pruss A, Galant K, Giedrys-Kalemba S. [Analysis of screening tests for Streptococcus agalactiae in pregnant women from the West Pomeranian region]. Analiza badan przesiewowych w kierunku Streptococcus agalactiae u kobiet w ciazy z regionu Pomorza Zachodniego **2015**; 86(8): 616-21.
91. Zatsiorskaia SL. Evaluation of various culture media for use in isolating group B streptococci from clinical specimens. Available at: http://ovidsp.ovid.com/ovidweb.cgi?T=JS&PAGE=reference&D=emed2&NEWS=N&AN=2469830.
92. Gyaneshwar R, Nsanze H, Singh KP, Pillay S, Seruvatu I. The prevalence of sexually transmitted disease agents in pregnant women in Suva. Australian and New Zealand Journal of Obstetrics and Gynaecology **1987**; 27(3): 213-5.
93. Brochet M, Couvé E, Bercion R, Sire J-M, Glaser P. Population Structure of Human Isolates of Streptococcus agalactiae from Dakar and Bangui. Journal of Clinical Microbiology **2009**; 47(3): 800-3.
94. Mitima KT, Ntamako S, Birindwa AM, et al. Prevalence of colonization by Streptococcus agalactiae among pregnant women in Bukavu, Democratic Republic of the Congo. Journal of infection in developing countries **2014**; 8(9): 1195-200.
95. Capan-Melser M, Ngoma GM, Akerey-Diop D, et al. Evaluation of intermittent preventive treatment of malaria against group B streptococcus colonization in pregnant women: A nested analysis of a randomized controlled clinical trial of sulfadoxine/pyrimethamine versus mefloquine. Journal of Antimicrobial Chemotherapy **2014**; 70(6): 1898-902.
96. Ferjani A, Ben Abdallah H, Ben Saida N, Gozzi C, Boukadida J. Vaginal colonization of the Streptococcus agalactiae in pregnant woman in Tunisia: Risk factors and susceptibility of isolates to antibiotics. Bulletin de la Societe de Pathologie Exotique **2006**; 99(2): 99-102.
97. Ben Hamouda S, Daaloul W, Chaouachi S, et al. Prospective epidemiologic study of group B streptococcus vaginal colonization in pregnant woman. Tunisie Medicale **2008**; 86(3): 249-54.
98. Jerbi M, Hidar S, Hannachi N, et al. Risk factors for group B streptococcal colonization in term pregnant women: prospective study about 294 cases. Gynecologie Obstetrique Fertilite **2007**; 35(4): 312-6.
99. Shabayek SAAEk, Abdalla SM, Abouzeid AMH. Vaginal carriage and antibiotic susceptibility profile of group B Streptococcus during late pregnancy in Ismailia, Egypt. Journal of Infection and Public Health **2009**; 2(2): 86-90.
100. Abdelmoaty H.; Zaki W. MK. Prevalence and antibiotic susceptibility of anogenital group b streptococci colonization in pregnant women: A hospital based study Egyptian Journal of Medical Laboratory Sciences **2009**; 18(2): 53-9.

101. Mohamed Sadaka S, Abdelsalam Aly H, Ahmed Meheissen M, Orief YI, Mohamed Arafa B. Group B streptococcal carriage, antimicrobial susceptibility, and virulence related genes among pregnant women in Alexandria, Egypt. Alexandria Journal of Medicine **2017**: no pagination.
102. Mahmoud M, Yahyaoui, G., Benseddik, M., Chaara, H., Melhouf, M.A. . Group b streptococcus screening during the third quarter of pregnancy in the Chu Hassan II of Fez Revue Tunisienne D'Infectiologie **2010**; 5(1): 12-5.
103. Benbachir M, El Mdaghri N, Lahlou D, Mesbahi M. Etude du portage de Streptococcus agalactiae et de Listeria monocyto genes chez la femme marocaine. Medecine et Maladies Infectieuses **1983**; 13(12): 793-7.
104. Towers CV, Rumney PJ, Asrat T, Preslicka C, Ghamsary MG, Nageotte MP. The accuracy of late third-trimester antenatal screening for group B streptococcus in predicting colonization at delivery. American Journal of Perinatology **2010**; 27(10): 785-9.
105. Panda B, Iruretagoyena I, Stiller R, Panda A. Antibiotic resistance and penicillin tolerance in ano-vaginal group B streptococci. Journal of Maternal-Fetal and Neonatal Medicine **2009**; 22(2): 111-4.
106. Turrentine MA, Ramirez MM. Recurrence of group B streptococci colonization in subsequent pregnancy. Available at: http://ovidsp.ovid.com/ovidweb.cgi?T=JS&PAGE=reference&D=emed8&NEWS=N&AN=2009310224. Accessed (Turrentine, Ramirez) Department of Obstetrics and Gynecology, Kelsey Research Foundation, University of Texas Health Science Center at Houston, Houston, TX.
107. Chen KT, Huard RC, Della-Latta P, Saiman L. Prevalence of methicillin-sensitive and methicillin-resistant Staphylococcus aureus in pregnant women. Obstetrics and gynecology **2006**; 108(3 Pt 1): 482-7.
108. Campbell JR, Hillier SL, Krohn MA, Ferrieri P, Zaleznik DF, Baker CJ. Group B streptococcal colonization and serotype-specific immunity in pregnant women at delivery. Obstet Gynecol **2000**; 96.
109. Bland ML, Vermillion ST, Soper DE, Austin M. Antibiotic resistance patterns of group B streptococci in late third-trimester rectovaginal cultures. American journal of obstetrics and gynecology **2001**; 184(6): 1125-6.
110. Lin FY, Weisman LE, Azimi P, et al. Assessment of intrapartum antibiotic prophylaxis for the prevention of early-onset group B Streptococcal disease. Pediatr Infect Dis J **2011**; 30(9): 759-63.
111. Delport J, Dawson L, Kramer J, Raedeke M. Prevalence of non-hemolytic and non-typeable group B Streptococcus isolates from screening specimens of pregnant women. Available at: http://ovidsp.ovid.com/ovidweb.cgi?T=JS&PAGE=reference&D=emed9&NEWS=N&AN=70141075. Accessed (Delport, Dawson, Kramer, Raedeke) Regina QuAppelle Health Region, Regina, Canada.
112. Spaetgens R, DeBella K, Ma D, Robertson S, Mucenski M, Davies HD. Perinatal antibiotic usage and changes in colonization and resistance rates of group B streptococcus and other pathogens. Obstetrics and gynecology **2002**; 100(3): 525-33.
113. Lavergne V, Laverdiere M, Duchesne A, et al. Prenatal culture-based screening of Streptococcus agalactiae colonisation: resistance against erythromycin and clindamycin. European journal of clinical microbiology & infectious diseases : official publication of the European Society of Clinical Microbiology **2006**; 25(8): 532-4.
114. Wenman WM, Tataryn IV, Joffres MR, et al. Demographic, clinical and microbiological characteristics of maternity patients: A Canadian clinical cohort study. Available at: http://ovidsp.ovid.com/ovidweb.cgi?T=JS&PAGE=reference&D=emed5&NEWS=N&AN=2002435363. Accessed (Wenman, Joffres, Pearson, Albritton) Department of Pediatrics, Edmonton, Alta., Canada.

115. Davies HD, Adair C, McGeer A, et al. Antibodies to capsular polysaccharides of group B Streptococcus in pregnant Canadian women: relationship to colonization status and infection in the neonate. J Infect Dis **2001**; 184.
116. Barcaite E, Bartusevicius A, Tameliene R, Maleckiene L, Vitkauskiene A, Nadisauskiene R. Group B streptococcus and Escherichia coli colonization in pregnant women and neonates in Lithuania. International Journal of Gynecology and Obstetrics **2012**; 117(1): 69-73.
117. Bjarnadóttir I1 KK, Hauksson A, Vilbergsson G, Pálsson G, Dagbjartsson A. Carriage of group B beta-haemolytic streptococci among pregnant women in Iceland and colonisation of their newborn infants. Laeknabladid **2003**; 89(2): 111-5.
118. Stokholm J, Schjorring S, Eskildsen CE, et al. Antibiotic use during pregnancy alters the commensal vaginal microbiota. Clinical microbiology and infection : the official publication of the European Society of Clinical Microbiology and Infectious Diseases **2014**; 20(7): 629-35.
119. Hansen SM, Uldbjerg N, Kilian M, Sørensen UBS. Dynamics of Streptococcus agalactiae Colonization in Women during and after Pregnancy and in Their Infants. Journal of Clinical Microbiology **2004**; 42(1): 83-9.
120. Hassan IA, Onon TS, Weston D, et al. A quantitative descriptive study of the prevalence of carriage (colonisation) of haemolytic streptococci groups A, B, C and G in pregnancy. Journal of Obstetrics and Gynaecology **2011**; 31(3): 207-9.
121. Afshar B, Vickers A, Morton K, et al. DEVANI UK clinical screening study for maternal carriage of Streptococcus agalactiae. Available at: http://ovidsp.ovid.com/ovidweb.cgi?T=JS&PAGE=reference&D=emed10&NEWS=N&AN=70599087. Accessed (Afshar, Vickers, Morton, Demertzi, Hassan, Stock, Telford, Orefici, Efstratiou) EdinburghUnited Kingdom.
122. Jones N, Oliver K, Jones Y, Haines A, Crook D. Carriage of group B streptococcus in pregnant women from Oxford, UK. J Clin Pathol **2006**; 59.
123. Brigtsen AK, Jacobsen AF, Dedi L, Melby KK, Fugelseth D, Whitelaw A. Maternal colonization with Group B streptococcus is associated with an increased rate of infants transferred to the neonatal intensive care unit. Neonatology **2015**; 108: 157-63.
124. Hakansson S, Axemo P, Bremme K, et al. Group B streptococcal carriage in Sweden: a national study on risk factors for mother and infant colonisation. Acta Obstet Gynecol Scand **2008**; 87.
125. Castellano-Filho DS, da Silva VL, Nascimento TC, Vieira MT, Diniz CG. Detection of group B Streptococcus in Brazilian pregnant women and antimicrobial susceptibility patterns. Brazilian Journal of Microbiology **2010**; 41(4): 1047-55.
126. Zusman AS, Baltimore RS, Fonseca SNS. Prevalence of maternal group B streptococcal colonization and related risk factors in a Brazilian population. Brazilian Journal of Infectious Diseases **2006**; 10(4): 242-6.
127. Pogere. Prevalence of group B Streptococcus in Pregnant Women from a prenatal care centre. Revista Brasileira de Ginecologia e Obstetricia **2005**; 27(4): 174-80.
128. Benchetrit LC, Francalanzza S.L., Peregrino H., Camelo A., Sanches A. Carriage of Streptococcus agalactiae in women and neonates and distribution of serological types: A Study in brazil Journal of Clinical Microbiology **1982**; 15(5): 787.
129. Giraldo PC, Araujo ED, Junior JE, do Amaral RLG, Passos MRL, Goncalves AK. The prevalence of urogenital infections in pregnant women experiencing preterm and full-term labor. Infectious diseases in obstetrics and gynecology **2012**; 2012: 878241.
130. Rocchetti TT, Marconi C, Rall VLM, Borges VTM, Corrente JE, Da Silva MG. Group B streptococci colonization in pregnant women: Risk factors and evaluation of the vaginal flora. Archives of Gynecology and Obstetrics **2011**; 283(4): 717-21.

131. Linhares JJ, Neto PGC, Vasconcelos JLM, et al. Prevalence of the colonization by streptococcus agalactiae in pregnant women from a maternity in ceará, Brazil, correlating with perinatal outcomes. Revista Brasileira de Ginecologia e Obstetricia **2011**; 33(12): 395-400.
132. Pires TS, Turchi M, Andre MCDPB, et al. Maternal group B Streptococcus colonization: Prevalence, risk factors, phenotypical and genotypical characteristics in a Brazilian population. Available at: http://ovidsp.ovid.com/ovidweb.cgi?T=JS&PAGE=reference&D=emed9&NEWS=N&AN=70125978. Accessed (Pires, Schmaltz, Peres) Hospital Materno Infantil, Goiania, Brazil.
133. Costa ALDR, Lamy Filho F, Chein MBDC, Brito LMO, Lamy ZC, Andrade KL. Prevalence of colonization by group B Streptococcus in pregnant women from a public maternity of Northwest region of Brazil. Revista Brasileira de Ginecologia e Obstetricia **2008**; 30(6): 274-80.
134. Marconi C, Rocchetti TT, Rall VLM, de Carvalho LR, Borges VTM, da Silva MG. Detection of streptococcus agalactiae colonization in pregnant women by using combined swab cultures: Cross-sectional prevalence study. Available at: http://www.scielo.br/pdf/spmj/v128n2/a03v1282.pdf

http://ovidsp.ovid.com/ovidweb.cgi?T=JS&PAGE=reference&D=emed9&NEWS=N&AN=2010452699. Accessed (Marconi, Rocchetti, Rall, de Carvalho, Borges, da Silva) Department of Pathology, Faculdade de Medicina de Botucatu, Universidade Estadual Paulista (Unesp), Botucatu, Sao Paulo, Brazil.
135. Borger IL, d'Oliveira REC, Castro ACDd, Mondino SSBd. Streptococcus agalactiae em gestantes: prevalência de colonização e avaliação da suscetibilidade aos antimicrobianos. Revista Brasileira de Ginecologia e Obstetrícia **2005**; 27: 575-9.
136. Kiss FS, Rossato JS, Graudenz MS, Gutierrez LLP. Prevalence of Streptococcus agalactiae colonization in a sample of pregnant and non pregnant women from Porto Alegre, Rio Grande do Sul state, Brazil. Scientia Medica **2013**; 23(3).
137. Simoes JA, Alves VM, Fracalanzza SE, et al. Phenotypical characteristics of group B streptococcus in parturients. Braz J Infect Dis **2007**; 11.
138. El Beitune P, Duarte G, Maffei CML, Da Fonseca CK. Streptococcus agalactiae colonization among HIV-1-infected pregnant women: antimicrobial susceptibility evaluation. Journal of acquired immune deficiency syndromes (1999) **2007**; 44(2): 246.
139. Beitune PE, Duarte G, Maffei CML. Group B Streptococcus carriers among HIV-1-infected pregnant women according to gestational age and regional site of colonization: Rate of recovery from various sites [1]. Journal of Acquired Immune Deficiency Syndromes **2006**; 43(2): 247.
140. Benchetrit LC, Fracalanzza SE, Peregrino H, Camelo AA, Sanches LA. Carriage of group-B streptococci in women and newborn infants in Brazil. Transactions of the Royal Society of Tropical Medicine and Hygiene **1981**; 75(3): 473-4.
141. Chaves Júnior MB, João; Cardoso, Rosilene Fressatti; Pádua, Rúbia A. F.; Campanerut, Paula A. Z.; Carvalho, Maria Dalva de Barros; Pelloso, Sandra Marisa. Prevalência de Streptococcus do grupo B em gestantes / Prevalence of Streptococcus agalactiae in pregnant women. Femina **2008**; 36(5): 319-24
142. Nunes PR, Oliveira, M.S. Prevalence of Streptococcus Agalactiae in pregnant women of Porto Alegre, RS: Case Report Revista Brasileira de analises clinicas **2015**; 47(4): 178-80.
143. Siqueira F, Magalhaes D, Calderon I, Dias A. Prevalence of streptococcus agalactiae in a sample of brazilian federal district pregnant women. International Journal of Gynecology and Obstetrics **2015**; 131: E262.

144. Ronchi G, Satorres S. MI7 - Prevalence of streptococcus agalactiae colonization in pregnant women in the city of San Luis according to age and underlying diseases. Available at: http://ovidsp.ovid.com/ovidweb.cgi?T=JS&PAGE=reference&D=emed10&NEWS=N&AN=70713048. Accessed (Ronchi, Satorres) UNSL, Clinica y Maternidad CERHU, San Luis, Argentina.
145. Oviedo P, Pegels E, Laczeski M, Quiroga M, Vergara M. Phenotypic and genotypic characterization of Streptococcus agalactiae in pregnant women. First study in a province of Argentina. Brazilian Journal of Microbiology **2013**; 44(1): 253-8.
146. Quiroga M, Pegels E, Oviedo P, Pereyra E, Vergara M. Antibiotic susceptibility patterns and prevalence of group B Streptococcus isolated from pregnant women in Misiones, Argentina. Brazilian Journal of Microbiology **2008**; 39(2): 245-50.
147. Larcher JS, Capellino F, De Giusto R, et al. Group B streptococcus colonization during pregnancy and prevention of early onset of disease. Medicina **2005**; 65(3): 201-6.
148. **!!! INVALID CITATION !!! []**.
149. Toresani I, Limansky A, Bogado I, Guardati MC, Viale A, Sutich EG. Phenotypic and genotypic study of Streptococcus agalactiae in vagina of pregnant women in Argentina. Medicina **2001**; 61(3): 295-300.
150. Abarzua F, Argomedo C, Meissner A, et al. [Prevalence of anal-vaginal colonization of Streptococcus agalactiae in third trimester of pregnancy and susceptibility to macrolides and lincosamides, in pregnant women controlled at Clinica Alemana Temuco, Southern Chile]. Prevalencia de portacion vaginal-anal de Streptococcus agalactiae en el tercer trimestre de gestacion y susceptibilidad a macrolidos y lincosamidas, en mujeres embarazadas de Clinica Alemana Temuco, Chile **2014**; 31(3): 305-8.
151. Valdes ER, Pastene C.S., Grau M.T., Catalan M., Candia P., Juarez G., Caballero R. Prevalencia de colonizacion por Streptococcus Agalactiae (grupo B) en el tercher trimestre del embarazo pesquisado en medio de cultivo no selectivo. Revista chilena de obstetrica y ginecologia **2003**; 68(4): 305-8.
152. Tamariz Ortiz JH, Obregon Calero M, Jara Aguirre JC, Diaz Herrera J, Jefferson Cortez L, Guerra Allison H. Colonización vaginal y anorectal por Streptococcus agalactiae en gestantes de los Hospitales Nacionales Cayetano Heredia y Arzobispo Loayza. Revista Medica Herediana **2004**; 15: 144-50.
153. Laufer J, Scasso S, Sosa CG, Rodríguez-Cuns G, Alonso J, Pons JE. Group B streptococcus colonization among pregnant women in Uruguay. International Journal of Gynecology and Obstetrics **2009**; 104(3): 242-3.
154. Riera L, Benavides G, Morillo N. Colonization by group B Streptococcus in full term pregnancy and newborns in a community in Venezuela. Enfermedades Infecciosas y Microbiologia Clinica **1993**; 11(6): 295-8.
155. Pina-Carruyo M, Fuenmayor-Corvaia I, Gallegos B. Isolation of Streptococcus group B in pregnant women and their offspring. Investigacion Clinica **1979**; 20(2): 70-85.
156. García DA, Mojica ME, Méndez IA, et al. The prevalence of Streptococcus agalactiae in pregnant women attending the Hospital Militar Central, Bogota, Colombia, 2010. Revista Colombiana de Obstetricia y Ginecologia **2011**; 62(4): 302-7.
157. Ceballos CA, Loaiza N, Romero J, Ospina M, Vasquez EM. Characterization of pregnant women screened for Streptococcus agalactiae and its relationship with early neonatal sepsis, at the Clinica del Prado de Medellin (Colombia), 2010. Available at: http://www.elsevier.com/journals/infectio/0123-9392

http://ovidsp.ovid.com/ovidweb.cgi?T=JS&PAGE=reference&D=emed12&NEWS=N&AN=2014391652. Accessed (Ceballos, Loaiza, Romero, Ospina) Grupo de Microbiologia y Epidemiologia, Laboratorio Clinico Prolab, Medellin, Colombia.
158. Ortiz M, Fariña N, Sanabria R, et al. Frecuencia de colonización por Estreptococo grupo B en embarazadas de 35 a 37 semanas en el Hospital Materno-Infantil San Pablo. Memorias del Instituto de Investigaciones en Ciencias de la Salud **2013**; 11: 32-40.
159. Kovavisarach E, Ying WS-a, Kanjanahareutai S. Risk factors related to group B streptococcal colonization in pregnant women in labor. Journal of the Medical Association of Thailand = Chotmaihet thangphaet **2007**; 90(7): 1287-92.
160. Werawatakul Y, Taksaphan S, Pragarasung M, et al. Prevalence and risk factors of Streptococcus agalactiae (group B) colonization in mothers and neonatal contamination at Srinagarind Hospital. Journal of the Medical Association of Thailand **2001**; 84(10): 1422-9.
161. Tor-Udom S, Tor-Udom P, Hiriote W. The prevalence of Streptococcus agalactiae (Group B) colonization in pregnant women at Thammasat Hospital. Journal of the Medical Association of Thailand **2006**; 89(4): 411-4.
162. Turner C, Turner P, Po L, et al. Group B streptococcal carriage, serotype distribution and antibiotic susceptibilities in pregnant women at the time of delivery in a refugee population on the Thai-Myanmar border. BMC Infectious Diseases **2012**; 12.
163. Goto A, Vinh NQ, Minh PN, et al. Prevalence of and factors associated wirh reproductive tract infections among pregnant women in ten communes in Nghe An Province, Vietnam. Journal of Epidemiology **2005**; 15(5): 163-72.
164. Raj M, Razali N, Sulaiman S. Screening of antenatal mothers and prevention of perinatal Group B streptococcal infection. Available at: http://jummec.um.edu.my/current

http://ovidsp.ovid.com/ovidweb.cgi?T=JS&PAGE=reference&D=emed12&NEWS=N&AN=2009494182. Accessed (Raj, Razali, Sulaiman) Department of Obstetrics and Gynaecology, Faculty of Medicine, University of Malaya, Kuala Lumpur 50603, Malaysia.
165. Lim CT, Thong MK, Parasakthi N, Ngeow YF. Group B Streptococcus: Maternal Carriage Rate and Early Neonatal Septicaemia. Annals of the Academy of Medicine Singapore **1997**; 26(4): 421-5.
166. Chua S, Arulkumaran S, Chow C, et al. Genital Group B Streptococcus carriage in the antenatal period: its role in prom and preterm labour. Singapore medical journal **1995**; 36(4): 383-5.
167. Chow KK, Tay L, Lam C. A prospective study of group B streptococcal colonization in parturient mothers and their infants. Annals of the Academy of Medicine Singapore **1981**; 10(1): 79-83.
168. Cutland CL, Madhi SA, Zell ER, et al. Chlorhexidine maternal-vaginal and neonate body wipes in sepsis and vertical transmission of pathogenic bacteria in South Africa: a randomised, controlled trial. Available at: http://ovidsp.ovid.com/ovidweb.cgi?T=JS&PAGE=reference&D=emed9&NEWS=N&AN=2009621825. Accessed (Cutland, Madhi, Kuwanda, Laque, Groome, Adrian, Klugman) Department of Science and Technology, National Research Foundation, Vaccine Preventable Diseases and Medical Research Council, Soweto, South Africa.
169. Kwatra G, Adrian PV, Shiri T, Buchmann EJ, Cutland CL, Madhi SA. Serotype-specific acquisition and loss of group B Streptococcus recto-vaginal colonization in late pregnancy. PLoS ONE **2014**; 9(6).
170. Bolukaoto JY, Monyama CM, Chukwu MO, et al. Antibiotic resistance of Streptococcus agalactiae isolated from pregnant women in Garankuwa, South Africa. BMC Research Notes **2015**; 8: 364.
171. Madzivhandila M, Adrian PV, Cutland CL, Kuwanda L, Schrag SJ, Madhi SA. Serotype distribution and invasive potential of group B streptococcus isolates causing disease in infants and colonizing maternal-newborn dyads. PLoS ONE **2011**; 6(3).

172. Monyama MC, Bolukaoto JY, Chukwu MO, et al. Group B streptococcus colonisation in pregnant women at Dr. George Mukhari Hospital, South Africa. Southern African Journal of Epidemiology and Infection **2016**; 31(3): 17-21.
173. Chukwu MO, Mavenyengwa RT, Monyama CM, et al. Antigenic distribution of streptococcus agalactiae isolates from pregnant women at garankuwa hospital - South Africa. GERMS **2015**; 5(4): 125-33.
174. Dangor Y, Said M, Kwatra G, Madhi S, Mbelle N, Ismail F. Prevalence and characterization of group B streptococcus among pregnant women at a tertiary hospital in South Africa. International Journal of Infectious Diseases **2016**; 45: 221-2.
175. Sharmila V, Joseph NM, Babu TA, Chaturvedula L, Sistla S. Genital tract group B streptococcal colonization in pregnant women: A south Indian perspective. Journal of Infection in Developing Countries **2011**; 5(8): 592-5.
176. Kulkarni AA. PSG, Dharmadhikari C.A., Kulkarni R.D. . Colonization of Pregnant Women and their Newborn infants with group-B Streptococci. Indian Journal of Medical Microbiology **2001**; 19: 1-4.
177. Hajare V, Madhavi L.H.,, Singh H.K.G. Antibiogram of group b streptococci isolated from the vagina of pregnant women in third trimester of pregnancy. People's Journal of Scientific Research **2012**; 5(2): 22-6.
178. Madhavi H, Hajare V, Singh HKG. Carriage of group - B streptococci in pregnant women attending antenatal clinic at teaching hospital at Gulbarga, Karnataka state. Pravara Medical Review **2011**; 6(2): 20-3.
179. Goyal R, Singh NP, Lal P, Gupta P. Group B Streptococcus colonisation in obstetric cases in a tertiary care hospital in Delhi, India. Annals of tropical paediatrics **2004**; 24(2): 189-90.
180. Dalal BS, Lahiri A, Parel CC. Carriage rate of group B streptococci in pregnant women and evaluation of different isolation media. Journal of the Indian Medical Association **1998**; 96(12): 360-6.
181. Mani V, Jadhav M, Sivadasan K, Thangavelu CP, Rachel M, Prabha J. Maternal and neonatal colonization with group B Streptococcus and neonatal outcome. Indian pediatrics **1984**; 21(5): 357-63.
182. Kishore K, Deorari AK, Paul VK, Singh M, Bhujwala RA. Group B streptococcus colonization & neonatal outcome in north India. The Indian journal of medical research **1986**; 84: 492-4.
183. Nagar. Unpublished Thesis, **2007**.
184. Rajaratnam A, Kuruvilla T.S., Antony B. Prevalence of group B streptococcal colonization among pregnant women in a tertiary care hospital in coastal karnataka. International journal of applied biology and pharmaceutical technology **2013**; 4(1): 308-10.
185. Konikkara KP, Baliga S, Shenoy S, Bharati B. Evaluation of culture, antigen detection and polymerase chain reaction for detection of vaginal colonization of group B streptococcus in pregnant women. Journal of Clinical and Diagnostic Research **2014**; 8(2): 47-9.
186. Muthusami A, Devi CS, Kanungo R, et al. Vaginal colonization as a risk factor for the development of neonatal sepsis. Available at: http://ovidsp.ovid.com/ovidweb.cgi?T=JS&PAGE=reference&D=emed8&NEWS=N&AN=2008198176. Accessed (Devi C., Kanungo, Shashikala, Srinivasan) Department of Clinical Microbiology, Puducherry - 605 014, India.
187. Chaudhary U, Sabherwal U, Chugh TD. Prevalence of group B streptococci in obstetrical cases. Indian Journal of Medical Research **1981**; 73(5): 710-4.
188. Dechen TC, Sumit K, Ranabir P. Correlates of Vaginal Colonization with Group B Streptococci among Pregnant Women. Journal of Global Infectious Diseases **2010**; 2(3): 236-41.

189. Das A, Ray P, Sharma M, Gopalan S. Rapid diagnosis of vaginal carriage of group B beta haemolytic streptococcus by an enrichment cum antigen detection test. Available at: http://ovidsp.ovid.com/ovidweb.cgi?T=JS&PAGE=reference&D=emed6&NEWS=N&AN=2003483498. Accessed (Das, Ray, Sharma) Department of Medical Microbiology, Postgrad. Inst. of Med. Educ./Res., Chandigarh 160012, India.
190. Patil K.P. SSS, Nagmoti M.B., Swamy M.K. Group B Streptococci Colonization in Pregnant Women: Is Screening Necessary? South Asian Feder Obst Gyne **2013**; 5(2): 64-7.
191. Chaudhary M, Rench MA, Baker CJ, Singh P, Hans C, Edwards MS. Group B Streptococcal Colonization Among Pregnant Women in Delhi, India. Pediatric Infectious Disease Journal **2016**: no pagination.
192. Chaudhry BY, Akhtar N, Balouch AH. Vaginal carriage rate of group B Streptococcus in pregnant women and its transmission to neonates. Journal of Ayub Medical College, Abbottabad : JAMC **2010**; 22(4): 167-70.
193. Kirmani N, hassan T.J., Jafarey S.N., Hafiz S. . Carriage of beta haemolytic streptococci (BHS) in pregnant women and acquisition by neonates Journal of the Pakistan Medical Association **1994**.
194. Akhtar T, Zai S, Khatoon J, Zohra A, Roghani MT, Ahmad A. A study of group B streptococcal colonization and infection in newborns in Pakistan. Journal of tropical pediatrics **1987**; 33(6): 302-4.
195. Hafeez AAS, Abbasi S.A. Normal vaginal flora in pregnant women. Journal of Pakistan Institute of Medical Sciences **1997**; 7(437-440).
196. Munir SI, Waheed K, Khanum A, Iqbal R, Eusaph AZ, Hanif A. Frequency of Group B Streptococci in Pregnant Women in a Tertiary Care Hospital. Journal of the College of Physicians and Surgeons--Pakistan : JCPSP **2016**; 26(1): 27-30.
197. Chan GJ, Modak JK, Mahmud AA, Baqui AH, Black RE, Saha SK. Maternal and neonatal colonization in Bangladesh: Prevalences, etiologies and risk factors. Journal of Perinatology **2013**; 33(12): 971-6.
198. Hadavand S, Ghafoorimehr F, Rajabi L, Davati A, Zafarghandi N. Frequency of group B Streptococcal colonization in pregnant women aged 35- 37 weeks in clinical centers of shahed university, Tehran, Iran. Iranian Journal of Pathology **2015**; 10(2): 120-6.
199. Tajbakhsh S, Norouzi Esfahani M, Emaneini M, Motamed N, Rahmani E, Gharibi S. Identification of Streptococcus agalactiae by fluorescent in situ hybridization compared to culturing and the determination of prevalence of Streptococcus agalactiae colonization among pregnant women in Bushehr, Iran. BMC Infectious Diseases **2013**; 13(1).
200. Shirazi M, Abbariki E, Hafizi A, Shahbazi F, Bandari M, Dastgerdy E. The prevalence of group B Streptococcus colonization in iranian pregnant women and its subsequent outcome. International Journal of Fertility and Sterility **2014**; 7(4): 267-70.
201. Absalan M, Eslami G, Zandi H, Mosaddegh A, Vakili M, Khalili MB. Prevalence of recto-vaginal colonization of group B streptococcus in pregnant women. Journal of Isfahan Medical School **2013**; 30(220): 2367-75.
202. Namavar Jahromi B, Poorarian S, Poorbarfehee S. The prevalence and adverse effects of group B streptococcal colonization during pregnancy. Arch Iran Med **2008**; 11.
203. Hamedi A, Akhlaghi F, Seyedi SJ, Kharazmi A. Evaluation of group B streptococci colonization rate in pregnant women and their newborn. Acta Medica Iranica **2012**; 50(12): 805-8.
204. Seyyed EZ, Toossi E, Jalalvand A, Sajadi M. Group B Streptococci investigation in pre-term labors. Medical archives (Sarajevo, Bosnia and Herzegovina) **2013**; 67(2): 124-5.

205. Hassanzadeh P, Motamedifar M, Gharaghani MN. Carriage rate of group B streptococci in pregnant women in three teaching hospitals in Shiraz, Iran. Medical Principles and Practice **2011**; 20(3): 277-82.
206. Moghaddam MN. Recto-vaginal colonization of group B streptococcus in pregnant women referred to a hospital in Iran and its effect on lactobacillus normal flora. Journal of Biological Sciences **2010**; 10(2): 166-9.
207. Fatemi F, Pakzad P, Zeraati H, et al. Comparative molecular and microbiologic diagnosis of vaginal colonization by group B streptococcus in pregnant women during labor. Available at: http://www.mums.ac.ir/shares/basic_medical/basicmedjou/89/fall/a4.pdf

http://ovidsp.ovid.com/ovidweb.cgi?T=JS&PAGE=reference&D=emed9&NEWS=N&AN=2010542048. Accessed (Fatemi, Akhondi, Chamani-Tabriz) Reproductive Biotechnology Research Center, Avicenna Research Institute, ACECR, Tehran, Iran, Islamic Republic of.
208. Mansouri S, Ghasami E, Najad NS. Vaginal colonization of Group B streptococci during late pregnancy in Southeast of Iran: Incidence, serotype distribution and susceptibility to antibiotics. Journal of Medical Sciences **2008**; 8(6): 574-8.
209. Aali BS, Abdollahi H., Nakhaee, N., Davazdahemami Z., Mehdizadeh, A. The association of preterm labor with vaginal colonization of group b streptococci. Iranian Journal of Medical Sciences **2007**; 5(4): 191-4.
210. Rabiee S, Arab M, Mashouf RY. Epidemiologic pattern of vaginal colonization by group B Streptococcus in pregnant women in Hamadan, Central west of Iran. Iranian Journal of Medical Sciences **2006**; 31(2): 106-8.
211. Bornasi H, Rad EG, Fard-Mousavi N, Zand S, Abtahi H. Antibiotic resistance profile and capsular serotyping of streptococcus agalactiae isolated from pregnant women between 35 to 37 weeks of pregnancy. Koomesh **2016**; 17(2): 352-7.
212. Goudarzi G, Ghafarzadeh M, Shakib P, Anbari K. Culture and Real-Time PCR Based Maternal Screening and Antibiotic Susceptibility for Group B Streptococcus: An Iranian Experience. Global journal of health science **2015**; 7(6): 233-9.
213. Numanovic F, Smajlovic J, Gegic M, et al. Presence and resistance of Streptococcus agalactiae in vaginal specimens of pregnant and adult non-pregnant women and association with other aerobic bacteria. Medicinski Glasnik **2017**; 14(1): 98-105.
214. Müller-Vranješ A, Puntarić D, Čuržik D, et al. Prevalence and significance of vaginal group B streptococcus colonization in pregnant women from Osijek, Croatia. Collegium Antropologicum **2011**; 35(1): 21-6.
215. Trischler-Čeke Z, Semenić-Rutko M, Biljan D, et al. Prevalence and resistance of streptococcus agalactiae isolated from vagina and anorectum of pregnant women during the first organised screening of pregnant women for the presence of streptococcus agalactiaea in Croatia. Paediatria Croatica **2010**; 54(1): 19-24.
216. Liébana-Martos MDC, Cabrera-Alavargonzalez J, Rodríguez-Granger J, et al. Serotypes and antibiotic resistance patterns in beta-hemolytic Streptococcus agalactiae isolates in colonized mothers and newborns with invasive disease. Enfermedades Infecciosas y Microbiologia Clinica **2015**; 33(2): 84-8.
217. Dadvand P, Basagana X, Figueras F, Sunyer J, Nieuwenhuijsen MJ. Climate and group B streptococci colonisation during pregnancy: Present implications and future concerns. BJOG: An International Journal of Obstetrics and Gynaecology **2011**; 118(11): 1396-400.
218. Marimon JM, Valiente A, Ercibengoa M, Garcia-Arenzana JM, Perez-Trallero E. Erythromycin resistance and genetic elements carrying macrolide efflux genes in Streptococcus agalactiae. Available at: http://ovidsp.ovid.com/ovidweb.cgi?T=JS&PAGE=reference&D=emed7&NEWS=N&AN=2005566940. Accessed (Marimon, Valiente, Ercibengoa, Garcia-Arenzana, Perez-Trallero) Servicio de Microbiologia, Hospital Donostia, Paseo Dr. Beguiristain s/n, 20014 San Sebastian, Spain.

219. Ramos JM, Milla A, Lopez-Garcia P, Gutierrez F. Colonization by Streptococcus agalactiae in foreign and spanish gestating women in the area of Elche (Spain). Available at: http://ovidsp.ovid.com/ovidweb.cgi?T=JS&PAGE=reference&D=emed9&NEWS=N&AN=2009222352. Accessed (Ramos, Gutierrez) Unidad de Enfermedades Infecciosas, Hospital General Universitario de Elche, Alicante, Spain.
220. Bayo M, Berlanga M, Agut M. Vaginal microbiota in healthy pregnant women and prenatal screening of group B streptococci (GBS). International microbiology : the official journal of the Spanish Society for Microbiology **2002**; 5(2): 87-90.
221. Mestres JB, De Mora MRC, Pazos AD. Detection of Streptococcus agalactiae in pregnant women. Impact on prophylaxis of early-neonatal sepsis. Available at: http://ovidsp.ovid.com/ovidweb.cgi?T=JS&PAGE=reference&D=emed8&NEWS=N&AN=2008262186. Accessed (Mestres) SIIC.
222. Rojo-Bezares B, Azcona-Gutierrez JM, Martin C, Jareno MS, Torres C, Saenz Y. Streptococcus agalactiae from pregnant women: Antibiotic and heavy-metal resistance mechanisms and molecular typing. Epidemiology and Infection **2016**; 144(15): 3205-14.
223. Berardi A, Rossi C, Guidotti I, et al. Factors associated with intrapartum transmission of group B Streptococcus. The Pediatric infectious disease journal **2014**; 33(12): 1211-5.
224. Savoia D, Gottimer C, Crocilla C, Zucca M. Streptococcus agalactiae in pregnant women: phenotypic and genotypic characters. J Infect **2008**; 56.
225. Roccasalva LS, Giummarra V, Valenti O, Scuderi MC, Furneri PM. Isolation and characterization of Streptococcus agalactiae in pregnant women. Available at: http://www.giog.it/materiale_cic/362_XXX_10/3139_isolamento/article.pdf

http://ovidsp.ovid.com/ovidweb.cgi?T=JS&PAGE=reference&D=emed8&NEWS=N&AN=2009118803. Accessed (Roccasalva, Giummarra, Valenti, Scuderi, Furneri) Universita degli Studi di Catania, Dipartimento di Scienze Microbiologiche e Scienze Ginecologiche.
226. Busetti M, D'Agaro P, Campello C. Group B streptococcus prevalence in pregnant women from North-Eastern Italy: advantages of a screening strategy based on direct plating plus broth enrichment. Journal of clinical pathology **2007**; 60(10): 1140-3.
227. Lijoi D, Capua ED, Ferrero S, et al. The efficacy of 2002 CDC guidelines in preventing perinatal group B Streptococcal vertical transmission: A prospective study. Available at: http://ovidsp.ovid.com/ovidweb.cgi?T=JS&PAGE=reference&D=emed8&NEWS=N&AN=2007150502. Accessed (Lijoi, Capua, Ferrero, Mistrangelo, Morano, Ragni) Department of Obstetrics and Gynaecology, San Martino Hospital, University of Genoa, Largo R. Benzi 10, 16132 Genoa, Italy.
228. De Luca C, Buono N, Santillo V, et al. Screening and management of maternal colonization with Streptococcus agalactiae: an Italian cohort study. The journal of maternal-fetal & neonatal medicine : the official journal of the European Association of Perinatal Medicine, the Federation of Asia and Oceania Perinatal Societies, the International Society of Perinatal Obstetricians **2016**; 29(6): 911-5.
229. Prifti E, Papanagiotou A, Kyriakopoulos TH, et al. Prevalence of Streptococcus agalactiae colonisation in pregnant women and antimicrobial resistance profiles. Available at: http://ovidsp.ovid.com/ovidweb.cgi?T=JS&PAGE=reference&D=emed10&NEWS=N&AN=70822732. Accessed (Prifti, Papanagiotou, Kyriakopoulos, Vlachos, Lianos, Metzidaki, Tsetsa, Apostolou, Tzanetou) AthensGreece.
230. Daskalakis G, Papapanagiotou A, Mesogitis S, Papantoniou N, Mavromatis K, Antsaklis A. Bacterial vaginosis and group B streptococcal colonization and preterm delivery in a low-risk population. Fetal Diagnosis and Therapy **2006**; 21(2): 172-6.

231. Tsolia M, Psoma M, Gavrili S, et al. Group B streptococcus colonization of Greek pregnant women and neonates: prevalence, risk factors and serotypes. Clin Microbiol Infect **2003**; 9.
232. Suara RO, Adegbola RA, Baker CJ, Secka O, Mulholland EK, Greenwood BM. Carriage of group B streptococci in pregnant Gambian mothers and their infants. Journal of Infectious Diseases **1994**; 170(5): 1316-9.
233. Le Doare K. JS, Darboe S., Warburton F., Gorringe A., Heath P.T., Kampmann, B. . Risk Factors for Group B Streptococcus colonisation and disease in Gambian Women and their infants Journal of infection **2016**; (S0163-4453(15)00406-5).
234. Uhiara JE. Group B streptococcal carriage among parturients and their neonates in Zaria, Nigeria. African journal of medicine and medical sciences **1993**; 22(3): 79-83.
235. Dawodu AH, Damole IO, Onile BA. Epidemiology of group B streptococcal carriage among pregnant women and their neonates: an African experience. Tropical and geographical medicine **1983**; 35(2): 145-50.
236. Onipede*1 A, Adefusi*, O., Adeyemi +1, A., Adejuyigbe#1, E., Oyelese*1, A., Ogunniyi* T. GROUP B STREPTOCOCCUS CARRIAGE DURING LATE PREGNANCY IN ILE-IFE,

NIGERIA. AFR J CLN EXPER MICROBIOL **2012**; 13(3): 135-43.
237. Olanisebe SB, Adetosoye AI. Determination of asymptomatic carrier rate of beta-haemolytic group B Streptococcus in vaginas of pregnant women in Ibadan, Nigeria. Zentralblatt fur Bakteriologie, Mikrobiologie, und Hygiene Series A, Medical microbiology, infectious diseases, virology, parasitology **1986**; 261(2): 248-53.
238. Onile BA. Group B streptococcal carriage in Nigeria. Transactions of the Royal Society of Tropical Medicine and Hygiene **1980**; 74(3): 367-70.
239. Onwuezobe I.A. ERA. Prevalence and associated risk factors of group b streptococcus in pregnant women attending antenatal care in a Nigerian Urban Hospital Ibom Medical Journal **2016**; 9(1): 1-7.
240. Nwachukwu N. U, Kanu I., Anyanwu E. Genital colonization of group b streptococcus at term pregnancy in Calabar, Nigeria The Internet Journal of Pediatrics and Neonatology **2006**; 7(2): 1-4.
241. Denis F, David P, David M, Chiron JP, Correa P. Les streptocoques du groupe B chez la femme enceinte et le nouveau-né en afrique de l'Ouest. Bilan d'une recherche systématique à Dakar. Medecine et Maladies Infectieuses **1979**; 9(5): 318-21.
242. Vinnemeier C.D.; Brust P. O-DE, Sarpong N., Sarfo E.Y., Bio Y., Rolling T., Dekker D., Adu-Sarkodie Y., Eberhardt K.A., May J., Cramer, J.P. Group B Streptococci Serotype distribution in pregnant women in Ghana: Assessement of potential coverage through future vaccines. Tropical Medicine and International Health **2015**; 20(11): 1516-24.
243. Balaka B, Agbere A, Dagnra A, Baeta S, Kessie K, Assimadi K. [Genital bacterial carriage during the last trimester of pregnancy and early-onset neonatal sepsis]. Portage genital bacterien au dernier trimestre de la grossesse et infection neonatale precoce **2005**; 12(5): 514-9.
244. Mounerou S, Y DA, Biova ACHgA, et al. Group B Streptococcal Carriage Rate in Vagina of Pregnant Women in Third Trimester in Lom¨¦, Togo. World Journal of Preventive Medicine **2015**; 3(1): 7-10.
245. David-Prince M, Ategbo S, De Souza AE, et al. Carriage of Streptococcus B in the mother and infant pair at birth. Apropos of 106 cases. Bulletin de la Societe de pathologie exotique (1990) **1991**; 84(5 Pt 5): 522-31.

246. Enweronu-Laryea CC, Damale NRK, Newman MJ. Prevalence of group B streptococcus in pregnant women attending a tertiary hospital in Ghana in 2001. Archives of Clinical Microbiology **2011**; 2(2).
247. Faye-Kette Achi H, Dosso M, Kacou A, et al. Genital carriage of Streptococcus group B in the pregnant woman in Abidjan (Ivory Coast). Bulletin de la Societe de pathologie exotique (1990) **1991**; 84(5 Pt 5): 532-9.
248. Karadağ FY, Hizel K, Gelişien O. Colonization of group B streptococci in pregnant women at delivery. Turk Jinekoloji ve Obstetrik Dernegi Dergisi **2013**; 10(1): 16-20.
249. Yenişehirli G, Bulut Y, Demirtürk F, Çalişkan AC. Antimicrobial susceptibilities and serotype distribution of Streptococcus agalactiae strains isolated from pregnant women. Mikrobiyoloji Bulteni **2006**; 40(3): 155-60.
250. Kadanali A, Altoparlak Ü, Kadanali S. Maternal carriage and neonatal colonisation of group B streptococcus in eastern Turkey: Prevalence, risk factors and antimicrobial resistance. International Journal of Clinical Practice **2005**; 59(4): 437-40.
251. Eren A, Küçükercan M, Oğuzoğlu N, Ünal N, Karateke A. The carriage of group B streptococci in Turkish pregnant women and its transmission rate in newborns and serotype distribution. Turkish Journal of Pediatrics **2005**; 47(1): 28-33.
252. Barbaros I, Murat C, Mehmet V, et al. The colonization incidence of group B streptococcus in pregnant women and their newborns in Istanbul. Pediatrics International **2005**; 47(1): 64-6.
253. Yucesoy G, Caliskan E, Karadenizli A, et al. Maternal colonisation with group B streptococcus and effectiveness of a culture-based protocol to prevent early-onset neonatal sepsis. International journal of clinical practice **2004**; 58(8): 735-9.
254. Arisoy AS, Altinisik B, Tunger O, Kurutepe S, Ispahi C. Maternal carriage and antimicrobial resistance profile of group B Streptococcus. Infection **2003**; 31(4): 244-6.
255. Altoparlak U, Kadanali A, Kadanali S. Genital flora in pregnancy and its association with group B streptococcal colonization. Available at: http://ovidsp.ovid.com/ovidweb.cgi?T=JS&PAGE=reference&D=emed6&NEWS=N&AN=2004499553. Accessed (Altoparlak) Dept. Microbiol. Clin. Microbiol., School of Medicine, Ataturk University, Erzurum, Turkey.
256. Akman I, Imir G, Dokmeci C, et al. Prevalence of group B streptococcus colonization and intrapartum antibiotic prophylaxis at Marmara University Hospital. Available at: http://ovidsp.ovid.com/ovidweb.cgi?T=JS&PAGE=reference&D=emed5&NEWS=N&AN=2001227910. Accessed (Akman, Imir, Dokmeci, Over, Ozek, Soyletir, Ceyhan) Department of Pediatrics, School of Medicine, Marmara University, Istanbul, Turkey.
257. Celebi S, Tuncel E, Babacan M. The prevalence of group B streptococcus in pregnant women and newborn infants in East Anatolia. Mikrobiyoloji Bulteni **1992**; 26(2): 149-54.
258. Gokalp A, Oguz A, Bakici Z, et al. [Neonatal group B colonization and maternal urogenital and anorectal system carriage]. Neonatal grup B streptokok kolonizasyonunun annelerdeki ve anorektal sistem tasiyiciligi ile iliskisi **1986**; 20(4): 248-55.
259. Gokalp AS, Bakici MZ. [Relationship between neonatal group B streptococcal colonization and the maternal urogenital and anorectal system carrier state]. Neonatal grup B streptokok kolonizasyonunun annelerdeki urogenital ve anorektal sistem tasiyiciligi ile iliskisi **1985**; 19(2): 65-72.
260. Gökalp A, Oğuz A, Bakici Z, et al. Neonatal group B streptococcal colonization and maternal urogenital or anorectal carriage. Turkish Journal of Pediatrics **1988**; 30(1): 17-23.

261. Ayata A, Guvenc H, Felek S, Aygun AD, Kocabay K, Bektas S. Maternal carriage and neonatal colonisation of group B streptococci in labour are uncommon in Turkey. Paediatric and Perinatal Epidemiology **1994**; 8(2): 188-92.
262. Alp F, Findik D, Dagi HT, Arslan U, Pekin AT, Yilmaz SA. Screening and genotyping of group B streptococcus in pregnant and non-pregnant women in Turkey. Journal of infection in developing countries **2016**; 10(3): 222-6.
263. Al-Sweih N, Hammoud M, Al-Shimmiri M, Jamal M, Neil L, Rotimi V. Serotype distribution and mother-to-baby transmission rate of Streptococcus agalactiae among expectant mothers in Kuwait. Archives of Gynecology and Obstetrics **2005**; 272(2): 131-5.
264. Al-Sweih N, Maiyegun S, Diejomaoh M, et al. Streptococcus agalactiae (group B streptococci) carriage in late pregnancy in Kuwait. Medical Principles and Practice **2004**; 13(1): 10-4.
265. Ghaddar N, Alfouzan W, Anastasiadis E, et al. Evaluation of chromogenic medium and direct latex agglutination test for detection of group B streptococcus in vaginal specimens from pregnant women in Lebanon and Kuwait. Journal of Medical Microbiology **2014**; 63: 1395-9.
266. Sidky I, Thomas M. Prevalence of group B streptococcal infection colonisation in pregnant women and their offspring in the middle east. Journal of Obstetrics and Gynaecology **2002**; 22(2): 179-80.
267. Amin A, Abdulrazzaq YM, Uduman S. Group B streptococcal serotype distribution of isolates from colonized pregnant women at the time of delivery in United Arab Emirates. J Infect **2002**; 45.
268. Zamzami TY, Marzouki AM, Nasrat HA. Prevalence rate of group B streptococcal colonization among women in labor at King Abdul-Aziz University Hospital. Archives of Gynecology and Obstetrics **2011**; 284(3): 677-9.
269. El-Kersh TA, Al-Nuaim LA, Kharfy TA, Al-Shammary FJ, Al-Saleh SS, Al-Zamel FA. Detection of genital colonization of group B streptococci during late pregnancy. Saudi Medical Journal **2002**; 23(1): 56-61.
270. Gosling PJ, Morgos FW. Group B streptococci: colonization of women in labour and neonatal acquisition in the western region of Saudi Arabia. Journal of Hospital Infection **1983**; 4(3): 324.
271. Uduman SA, Chatterjee TK, Al-mouzan MI, Al-Suleiman S. Group B streptococci colonization among Saudi women in labor and neonatal acquisition. International Journal of Gynecology and Obstetrics **1985**; 23(1): 21-4.
272. Al-Suleiman SA, Farrag I, Kingsley TD, Uduman SA, Al-Mouzan MI. Third trimester colonisation and treatment of group b βhaemolytic streptococcus among obstetric patients in the eastern province of Saudi arabia. Journal of Obstetrics and Gynaecology **1991**; 11(6): 409-13.
273. Khan MA, Faiz A, Ashshi AM. Maternal colonization of group B streptococcus: prevalence, associated factors and antimicrobial resistance. Annals of Saudi medicine **2015**; 35(6): 423-7.
274. Seoud MN, A.H., Zalloua P., Boghossian N., Ezeddine J., Fakhoury H., Abboud J., Melki I., Araj G., Nacouzi G., Sanyoura M., Yunis K. Prenatal and neonatal group b streptococcus screening and serotyping in Lebanon: incidence and implications. Acta Obstetrica et Gynecologica **2010**; 89: 399-403.
275. Chaaya A, Chacar HR, Daoud M, et al. Screening of Streptococcus agalactiae (group B) in the perinatal period. Le Journal médical libanais The Lebanese medical journal **1996**; 44(4): 203-8.
276. Sunna E, el-Daher N, Bustami K, Na'was T. A study of group B streptococcal carrier state during late pregnancy. Tropical and geographical medicine **1991**; 43(1-2): 161-4.

277. Eisenberg VH, Raveh D, Meislish Y, et al. Prevention of early-onset neonatal group B streptococcal infection: is universal screening by culture universally applicable? The Israel Medical Association journal : IMAJ **2006**; 8(10): 698-702.
278. Marchaim D, Hallak M, Gortzak-Uzan L, Peled N, Riesenberg K, Schlaeffer F. Risk factors for carriage of group B streptococcus in southern Israel. Isr Med Assoc J **2003**; 5.
279. Eidelman AI, Rudensky B, Turgeman D, Nubani N, Schimmel MS, Isacsohn M. Epidemiology of group B streptococci colonization and disease in mothers and infants: update of ongoing 10-year Jerusalem study. Israel journal of medical sciences **1990**; 26(2): 71-3.
280. Drai-Hasid R. C-mR, Lev-Sagie A., Avital G., Block C., Moses A.E., Hochner-Celnikier D. Ritual Immersion in a Mikveh is associated with increased risk of group b streptococcal carrier state in israeli parturient women. Open Journal of Obstetrics and Gynecology **2015**; 5: 769-74
281. Chhin D, Pozzetto B, Teyssier G, et al. Relationship between cord blood vitamin D level and group B Streptococcus vaginal carriage rate in pregnant women. e-SPEN Journal **2013**; 8(4): e150-e4.
282. van der Mee-Marquet N, Jouannet C, Domelier AS, Arnault L, Lartigue MF, Quentin R. Genetic diversity of Streptococcus agalactiae strains and density of vaginal carriage. J Med Microbiol **2009**; 58.
283. Honderlick P, Gravisse J, Cahen P, Vignon D. Evaluation of 6 years of group B streptococcus (GBS) screening in near-term pregnant women. Pathologie Biologie **2010**; 58(2): 144-6.
284. Mereghetti L, Lanotte P, Rochoux A, et al. Application of the French guidelines for preventing neonatal group B streptococcal disease in a university hospital. Clinical Microbiology and Infection **2007**; 13(3): 322-4.
285. Chhuy T, Mansour G, Zejli A, Bouquigny C, Bock S, Abboud P. Group B streptococcus screening: A retrospective study in 1,674 pregnancies. Journal de Gynecologie Obstetrique et Biologie de la Reproduction **2005**; 34(4): 328-33.
286. Jauréguy F, Carton M, Teboul J, et al. Risk factors and screening strategy for group B streptococcal colonization in pregnant women: Results of a prospective study. Journal de Gynecologie Obstetrique et Biologie de la Reproduction **2003**; 32(2): 132-8.
287. Volumenie JL, Fernandez H, Vial M, Lebrun L, Frydman R. Neonatal group B streptococcal infection. Results of 33 months of universal maternal screening and antibioprophylaxis. European journal of obstetrics, gynecology, and reproductive biology **2001**; 94(1): 79-85.
288. El Aila NA, Tency I, Claeys G, et al. Genotyping of Streptococcus agalactiae (group B streptococci) isolated from vaginal and rectal swabs of women at 35-37 weeks of pregnancy. BMC Infectious Diseases **2009**; 9: 153.
289. Kunze M, Zumstein K, Markfeld-Erol F, et al. Comparison of pre- and intrapartum screening of group B streptococci and adherence to screening guidelines: a cohort study. European Journal of Pediatrics **2015**; 174(6): 827-35.
290. Kunze M, Ziegler A, Fluegge K, Hentschel R, Proempeler H, Berner R. Colonization, serotypes and transmission rates of group B streptococci in pregnant women and their infants born at a single University Center in Germany. Journal of Perinatal Medicine **2011**; 39(4): 417-22.
291. Brimil N, Barthell E, Heindrichs U, Kuhn M, Lutticken R, Spellerberg B. Epidemiology of Streptococcus agalactiae colonization in Germany. Int J Med Microbiol **2006**; 296.
292. Valkenburg-Van Den Berg AW, Sprij AJ, Oostvogel PM, et al. Prevalence of colonisation with group B Streptococci in pregnant women of a multi-ethnic population in the Netherlands. European Journal of Obstetrics Gynecology and Reproductive Biology **2006**; 124(2): 178-83.

293. Capanna F, Emonet SP, Cherkaoui A, Irion O, Schrenzel J, Martinez de Tejada B. Antibiotic resistance patterns among group B Streptococcus isolates: implications for antibiotic prophylaxis for early-onset neonatal sepsis. Swiss medical weekly **2013**; 143: w13778.
294. Rausch AV, Gross A, Droz S, Bodmer T, Surbek DV. Group B Streptococcus colonization in pregnancy: Prevalence and prevention strategies of neonatal sepsis. Journal of Perinatal Medicine **2009**; 37(2): 124-9.
295. Hafner E, Sterniste W, Rosen A, et al. Group B streptococci during pregnancy: a comparison of two screening and treatment protocols. American journal of obstetrics and gynecology **1998**; 179(3 Pt 1): 677-81.
296. Dahan-Saal J, Gérardin P, Robillard PY, et al. Determinants of group B streptococcus maternal colonization and factors related to its vertical perinatal transmission: Case-control study. Gynecologie Obstetrique Fertilite **2011**; 39(5): 281-8.
297. K.L. T. A study of group b streptococcus in Brisbane: The epidemiology, detection by PCR assay & serovar prevalence, **2006**.
298. Ko DWH, Zurynski Y, Gilbert GL. Group B streptococcal disease and genotypes in Australian infants. Available at: www.blackwell-science.com/jpc

http://ovidsp.ovid.com/ovidweb.cgi?T=JS&PAGE=reference&D=emed13&NEWS=N&AN=2015724381. Accessed (Ko, Gilbert) Centre for Infectious Diseases and Microbiology, Westmead Hospital, Sydney, NSW, Australia.
299. Grimwood K, Stone PR, Gosling IA, et al. Late antenatal carriage of group B Streptococcus by New Zealand women. Aust N Z J Obstet Gynaecol **2002**; 42.
300. Moyo SR, Maeland JA, Bergh K. Typing of human isolates of Streptococcus agalactiae (group B streptococcus, GBS) strains from Zimbabwe. Journal of Medical Microbiology **2002**; 51(7): 595-600.
301. Shen AD, Yang YY, Schollin J. Serotype distribution and antimicrobial susceptibility profiles of group B streptococcus strains from pregnant women in Beijing, 1994-99. Prenatal and Neonatal Medicine **2000**; 5(4): 230-5.
302. Shen A, Zhu Y, Zhang G, Yang Y, Jiang Z. Experimental study on distribution of serotypes and antimicrobial patterns of group B streptococcus strains. Chinese medical journal **1998**; 111(7): 615-8.
303. Van Elzakker E, Yahiaoui R, Visser C, et al. Epidemiology of and prenatal molecular distinction between invasive and colonizing group B streptococci in the Netherlands and Taiwan. European Journal of Clinical Microbiology and Infectious Diseases **2009**; 28(8): 921-8.
304. Yan Y, Hu H, Lu T, et al. Investigation of serotype distribution and resistance genes profile in group B Streptococcus isolated from pregnant women: a Chinese multicenter cohort study. APMIS : acta pathologica, microbiologica, et immunologica Scandinavica **2016**; 124(9): 794-9.
305. Lu B, Wang D, Zhou H, et al. Distribution of pilus islands and alpha-like protein genes of group B Streptococcus colonized in pregnant women in Beijing, China. European journal of clinical microbiology & infectious diseases : official publication of the European Society of Clinical Microbiology **2015**; 34(6): 1173-9.
306. Seo YS, Srinivasan U, Oh KY, et al. Changing molecular epidemiology of group B streptococcus in Korea. Journal of Korean medical science **2010**; 25(6): 817-23.
307. Lee HR, Song SH, Kim HB, Park KU, Song J. A rapid genotyping test for the simultaneous detection and subtyping of group B streptococci: The frequency of molecular subtypes of group B streptococci in Korea. European Journal of Clinical Microbiology and Infectious Diseases **2010**; 29(10): 1287-90.

308. Oh CE, Jang HO, Kim NH, Lee J, Choi EH, Lee HJ. Molecular serotyping of Group B streptococcus isolated from the pregnant women by polymerase chain reaction and sequence analysis. Korean Journal of Pediatric Infectious Diseases **2009**; 16(1): 47-53.
309. Wakimoto H, Wakimoto Y, Yano H, et al. [Antimicrobial susceptibility and serotype distribution in perinatal group B Streptococcus isolates--a 1999-2009 multicenter study]. Available at: http://ovidsp.ovid.com/ovidweb.cgi?T=JS&PAGE=reference&D=emed11&NEWS=N&AN=21560418. Accessed (Wakimoto) Nagoya City University School of Nursing.
310. Kimura K, Matsubara K, Yamamoto G, Shibayama K, Arakawa Y. Active screening of group B Streptococci with reduced penicillin susceptibility and altered serotype distribution isolated from pregnant women in Kobe, Japan. Japanese Journal of Infectious Diseases **2013**; 66(2): 158-60.
311. Cristea V-C, Duta M, Neacsu G. Screening for group B streptococcus: a private laboratory experience. Roumanian archives of microbiology and immunology **2011**; 70(2): 65-8.
312. Usein CR, Petrini A, Georgescu R, Grigore L, Străuţ M, Ungureanu V. Group B streptococcus colonization of Romanian women: phenotypic traits of isolates from vaginal swabs. Roumanian archives of microbiology and immunology **2009**; 68(4): 235-9.
313. Romanik M, Nowosielski K, Poręba R, Sioma-Markowska U, Martiroisian G, Groborz J. Streptococcus group B serotype distribution in anovaginal isolates of women in term pregnancy. Neuroendocrinology Letters **2014**; 35(4): 301-5.
314. Brzychczy-Wloch M, Gosiewski T, Bodaszewska M, Pabian W, Ochonska D, Heczko PB. Distribution of PFGE types of group B streptococci originating from Polish pregnant women in relation to capsular polysaccharides and surface protein genes. Available at: http://ovidsp.ovid.com/ovidweb.cgi?T=JS&PAGE=reference&D=emed9&NEWS=N&AN=70196304. Accessed (Brzychczy-Wloch, Gosiewski, Bodaszewska, Pabian, Ochonska, Heczko) CracowPoland.
315. Wolski B, Zegarska J, Adamczak R, Szymanski W, Kaczmarek A, Dorota M. Serotype I, II, III distribution and antimicrobial susceptibility of Streptococcus agalactiae among Polish delivering women. Available at: http://ovidsp.ovid.com/ovidweb.cgi?T=JS&PAGE=reference&D=emed9&NEWS=N&AN=70231032. Accessed (Wolski, Zegarska, Adamczak, Szymanski) Women's Diseases and Gynecological Oncology Department, Collegium Medicum in Bydgoszcz, Nicolaus Copernicus University, Torun, Poland.
316. Belard S, Toepfner N, Capan-Melser M, et al. Streptococcus agalactiae Serotype Distribution and Antimicrobial Susceptibility in Pregnant Women in Gabon, Central Africa. Scientific reports **2015**; 5: 17281.
317. Bergal A, Loucif L, Benouareth DE, Bentorki AA, Abat C, Rolain JM. Molecular epidemiology and distribution of serotypes, genotypes, and antibiotic resistance genes of Streptococcus agalactiae clinical isolates from Guelma, Algeria and Marseille, France. Eur J Clin Microbiol Infect Dis **2015**; 34(12): 2339-48.
318. Croak A, Abate G, Goodrum K, Modrzakowski M. Predominance of serotype V and frequency of erythromycin resistance in Streptococcus agalactiae in Ohio. American Journal of Obstetrics and Gynecology **2003**; 188(5): 1148-50.
319. Meehan M, Cunney R, Cafferkey M. Molecular epidemiology of group B streptococci in Ireland reveals a diverse population with evidence of capsular switching. European Journal of Clinical Microbiology and Infectious Diseases **2014**; 33(7): 1155-62.
320. Dore N, Bennett D, Kaliszer M, Cafferkey M, Smyth CJ. Molecular epidemiology of group B streptococci in Ireland: associations between serotype, invasive status and presence of genes encoding putative virulence factors. Epidemiol Infect **2003**; 131.

321. Jones N, Oliver K, Jones Y, Haines A, Crook D. Carriage of group B streptococcus in pregnant women from Oxford, UK. Journal of clinical pathology **2006**; 59(4): 363-6.
322. Brigtsen AK, Dedi L, Melby KK, et al. Comparison of PCR and serotyping of Group B Streptococcus in pregnant women: the Oslo GBS-study. Journal of microbiological methods **2015**; 108: 31-5.
323. Berg S, Trollfors B, Lagergard T, Zackrisson G, Claesson BA. Serotypes and clinical manifestations of group B streptococcal infections in western Sweden. Clin Microbiol Infect **2000**; 6.
324. Benchetrit LC, Fracalanzza SEL, Peregrino H, Camelo AA, Sanches LA. Carriage of Streptococcus agalactiae in women and neonates and distribution of serological types: A study in Brazil. Journal of Clinical Microbiology **1982**; 15(5): 787-90.
325. Soares GCT, Alviano DS, Santos GS, Alviano CS, Mattos-Guaraldi AL, Nagao PE. Prevalence of group B Streptococcus serotypes III and V in pregnant women of Rio de Janeiro, Brazil. Brazilian Journal of Microbiology **2013**; 44(3): 869-72.
326. Palmeiro JK, Dalla-Costa LM, Fracalanzza SEL, et al. Phenotypic and genotypic characterization of group B streptococcal isolates in Southern Brazil. Journal of Clinical Microbiology **2010**; 48(12): 4397-403.
327. Dhanoa A, Karunakaran R, Puthucheary SD. Serotype distribution and antibiotic susceptibility of group B streptococci in pregnant women. Epidemiology and infection **2010**; 138(7): 979-81.
328. Suhaimi MES, Desa MNM, Eskandarian N, et al. Characterization of a Group B Streptococcus infection based on the demographics, serotypes, antimicrobial susceptibility and genotypes of selected isolates from sterile and non-sterile isolation sites in three major hospitals in Malaysia. Journal of Infection and Public Health **2017**; 10(1): 14-21.
329. Beigverdi R, Jabalameli F, Mirsalehian A, et al. Virulence factors, antimicrobial susceptibility and molecular characterization of streptococcus agalactiae isolated from pregnant women. Available at: http://www.akademiai.com

http://ovidsp.ovid.com/ovidweb.cgi?T=JS&PAGE=reference&D=emed13&NEWS=N&AN=2015986252. Accessed (Beigverdi, Jabalameli, Mirsalehian, Emaneini) Department of Microbiology, School of Medicine, Tehran University of Medical Sciences, Tehran, Iran, Islamic Republic of.
330. Jannati E, Roshani M, Shahram H, Arzanlou M. Antibiotic resistance pattern and serotype distribution of Streptococcus agalactiae isolated from pregnant women, Ardabil, Iran. Available at: http://ovidsp.ovid.com/ovidweb.cgi?T=JS&PAGE=reference&D=emed10&NEWS=N&AN=70496609. Accessed (Jannati) Young Researcher Club, Islamic Azad University, Ardabil Branch, Ardabil, Iran, Islamic Republic of.
331. Martins ER, Pessanha MA, Ramirez M, et al. Analysis of group B streptococcal isolates from infants and pregnant women in Portugal revealing two lineages with enhanced invasiveness. Available at: http://ovidsp.ovid.com/ovidweb.cgi?T=JS&PAGE=reference&D=emed8&NEWS=N&AN=2007505096. Accessed (Martins, Pessanha, Ramirez, Melo-Cristino) Instituto de Microbiologia, Faculdade de Medicina, Universidade de Lisboa, Lisbon, Portugal.
332. Sadeh M, Firouzi R, Derakhshandeh A, Khalili MB, Kong F, Kudinha T. Molecular characterization of streptococcus agalactiae isolates from pregnant and non-pregnant women at yazd university hospital, Iran. Jundishapur Journal of Microbiology **2016**; 9(2): no pagination.
333. Puertas A, Liebana C, Vico I, Rodriguez-Granger J, Aguilar T, Carrillo MP. Distribution of serotypes of group B (GBS) streptococci isolated from newborn and pregnant women in Andalusia (Spain). Available at:

http://ovidsp.ovid.com/ovidweb.cgi?T=JS&PAGE=reference&D=emed9&NEWS=N&AN=70201291. Accessed (Puertas, Liebana, Vico, Rodriguez-Granger, Aguilar, Carrillo) Microbiology Service, Virgen de las Nieves Hospital, Granada, Spain.
334. Liebana C, Cabrera J, Moreno E, Rodriguez-Granger J, Navarro-Mari JM, Molina FS. Comparison of two capsular serotyping methods among isolates of streptococcus agalactiae from pregnant women. Available at: http://ovidsp.ovid.com/ovidweb.cgi?T=JS&PAGE=reference&D=emed9&NEWS=N&AN=70201289. Accessed (Liebana, Cabrera, Moreno, Rodriguez-Granger, Navarro-Mari, Molina) Microbiology Service, Virgen de las Nieves Hospital, Granada, Spain.
335. Perez-Ruiz M, Rodriguez-Granger JM, Bautista-Marin MF, Romero-Noguera J, Rosa-Fraile M. Genetic diversity of Streptococcus agalactiae strains colonizing the same pregnant woman. Epidemiology and infection **2004**; 132(2): 375-8.
336. Savoia D, Gottimer C, Crocilla C, Zucca M. Streptococcus agalactiae in pregnant women: Phenotypic and genotypic characters. Available at: http://ovidsp.ovid.com/ovidweb.cgi?T=JS&PAGE=reference&D=emed8&NEWS=N&AN=2008057105. Accessed (Savoia, Gottimer, Zucca) Department of Clinical and Biological Sciences, University of Turin, at S. Luigi Gonzaga Hospital, Regione Gonzole 10, 10043 Orbassano, TO, Italy.
337. Ekin IH, Gurturk K, Ilhan Z, Arabaci C, Gulaydin O. Detection of enzyme activities and their relation to serotypes of bovine and human group B streptococci. Journal of Medical Microbiology **2015**; 64(9): 985-9.
338. Ekin IH, Gurturk K. Characterization of bovine and human group B streptococci isolated in Turkey. Journal of Medical Microbiology **2006**; 55(5): 517-21.
339. Udo EE, Boswihi SS, Al-Sweih N. Genotypes and virulence genes in group B streptococcus isolated in the maternity hospital, Kuwait. Medical Principles and Practice **2013**; 22(5): 453-7.
340. Boswihi SS, Udo EE, Al-Sweih N. Serotypes and antibiotic resistance in group B streptococcus isolated from patients at the Maternity Hospital, Kuwait. Available at: http://jmm.sgmjournals.org/content/61/1/126.full.pdf+html

http://ovidsp.ovid.com/ovidweb.cgi?T=JS&PAGE=reference&D=emed10&NEWS=N&AN=2011696388. Accessed (Boswihi, Udo, Al-Sweih) Department of Microbiology, Faculty of Medicine, Kuwait University, Kuwait.
341. Hannoun A. SM, Khairallah M-T., Sabra A., Abi-Rached R., Bazi T., Yunis K.A., Araj G.F., Matar G.M. Correlation between Group B Streptococcal Genotypes, Their Antimicrobial Resistance Profiles, and Virulence Genese among Pregnant Women in Lebanon. International Journal of Microbiology **2009**; Article ID 796512.
342. Marchaim D, Efrati S, Melamed R, et al. Clonal variability of group B Streptococcus among different groups of carriers in southern Israel. European Journal of Clinical Microbiology and Infectious Diseases **2006**; 25(7): 443-8.
343. Bisharat N, Jones N, Marchaim D, et al. Population structure of group B streptococcus from a low-incidence region for invasive neonatal disease. Microbiology (Reading, England) **2005**; 151(Pt 6): 1875-81.
344. Van Der Mee-Marquet N, Jouannet C, Domelier AS, Arnault L, Lartigue MF, Quentin R. Genetic diversity of Streptococcus agalactiae strains and density of vaginal carriage. Available at: http://jmm.sgmjournals.org/cgi/reprint/58/2/169

http://ovidsp.ovid.com/ovidweb.cgi?T=JS&PAGE=reference&D=emed9&NEWS=N&AN=2009073172. Accessed (Van Der Mee-Marquet, Domelier, Lartigue, Quentin) Universite Francois-Rabelais, IFR 136, Faculte de Medecine, EA 3854 Bacteries et Risque Materno-Foetal, Tours, France.

345. Lamy M-C, Dramsi S, Billoet A, et al. Rapid detection of the "highly virulent" group B Streptococcus ST-17 clone. Microbes and infection / Institut Pasteur **2006**; 8(7): 1714-22.
346. von Both U, Ruess M, Mueller U, Fluegge K, Sander A, Berner R. A serotype V clone is predominant among erythromycin-resistant Streptococcus agalactiae isolates in a southwestern region of Germany. Journal of clinical microbiology **2003**; 41(5): 2166-9.
347. Muller AE, Valkenburg-van den Berg AW, Kreft D, Oostvogel PM, Sprij AJ, van Belkum A. Low rate of carriage of macrolide-resistant group B streptococci in pregnant women in The Netherlands. European Journal of Obstetrics Gynecology and Reproductive Biology **2008**; 137(1): 17-20.
348. van Elzakker E, Yahiaoui R, Visser C, et al. Epidemiology of and prenatal molecular distinction between invasive and colonizing group B streptococci in The Netherlands and Taiwan. Eur J Clin Microbiol Infect Dis **2009**; 28.
349. Fröhlicher S, Reichen G, Müller M, et al. Serotype distribution and antimicrobial susceptibility of group B streptococci in pregnant women: Results from a Swiss tertiary centre. Swiss Medical Weekly **2014**; 144.
350. Law KSK, Parmar P, Gregora M, Abbott J. A comparative study assessing the efficacy and acceptability of anorectal swabs for antenatal gbs screening. Journal of Medical Screening **2013**; 20(1): 46-8.
351. El Aila NA, Tency I, Claeys G, et al. Comparison of different sampling techniques and of different culture methods for detection of group B streptococcus carriage in pregnant women. BMC Infectious Diseases **2010**; 10.
352. Goudarzi G, Ghafarzadeh M, Shakib P, Anbari K. Culture and Real-Time PCR Based Maternal Screening and Antibiotic Susceptibility for Group B Streptococcus: An Iranian Experience. Global journal of health science **2015**; 7(6): 233-9.
353. Kieran E, Matheson M, Mann AG, Efstratiou AA, Butler K, Gorman W. Group B streptococcus (GBS) colonisation among expectant Irish mothers. Irish medical journal **1998**; 91(1): 21-2.
354. Hoogkamp-Korstanje JA, Gerards LJ, Cats BP. Maternal carriage and neonatal acquisition of group B streptococci. The Journal of infectious diseases **1982**; 145(6): 800-3.
355. Kovavisarach E, Sa-adying W, Kanjanahareutai S. Comparison of combined vaginal-anorectal, vaginal and anorectal cultures in detecting of group B streptococci in pregnant women in labor. Journal of the Medical Association of Thailand **2007**; 90(9): 1710-4.
356. Dillon HC, Jr., Gray E, Pass MA, Gray BM. Anorectal and vaginal carriage of group B streptococci during pregnancy. The Journal of infectious diseases **1982**; 145(6): 794-9.
357. Philipson EH, Palermino DA, Robinson A. Enhanced antenatal detection of group B streptococcus colonization. Obstetrics and gynecology **1995**; 85(3): 437-9.
358. Jamie WE, Edwards RK, Duff P. Vaginal-perianal compared with vaginal-rectal cultures for identification of group B streptococci. Available at: http://ovidsp.ovid.com/ovidweb.cgi?T=JS&PAGE=reference&D=emed6&NEWS=N&AN=2005148764. Accessed (Jamie, Edwards, Duff) Department of Obstetrics, Division of Maternal-Fetal Medicine, Univ. of Florida College of Medicine, Gainesville, FL, United States.
359. Quinlan JD, Hill DA, Maxwell BD, Boone S, Hoover F, Lense JJ. The necessity of both anorectal and vaginal cultures for group B streptococcus screening during pregnancy. The Journal of family practice **2000**; 49(5): 447-8.
360. Badri MS, Zawaneh S, Cruz AC, et al. Rectal colonization with group B streptococcus: relation to vaginal colonization of pregnant women. Journal of Infectious Diseases **1977**; 135(2): 308-12.

361. Platt MW, McLaughlin JC, Gilson GJ, Wellhoner MF, Nims LJ. Increased recovery of group B Streptococcus by the inclusion of rectal culturing and enrichment. Diagnostic microbiology and infectious disease **1995**; 21(2): 65-8.
362. Elsayed S, Gregson DB, Church DL. Comparison of direct selective versus nonselective agar media plus LIM broth enrichment for determination of group B streptococcus colonization status in pregnant women. Archives of Pathology and Laboratory Medicine **2003**; 127(6): 718-20.
363. Bosch-Mestres J, Martín-Fernández RM, Jiménez de Anta-Losada MT. Comparative study of three culture media for detecting group B Streptococcus colonization in pregnant women. Enfermedades Infecciosas y Microbiologia Clinica **2003**; 21(7): 346-9.
364. Silver HM, Struminsky J. A comparison of the yield of positive antenatal group B Streptococcus cultures with direct inoculation in selective growth medium versus primary inoculation in transport medium followed by delayed inoculation in selective growth medium. Available at: http://ovidsp.ovid.com/ovidweb.cgi?T=JS&PAGE=reference&D=emed4&NEWS=N&AN=1996260622. Accessed (Silver, Struminsky) Department of Obstetrics/Gynecology, 101 Dudley St., Providence, RI 02905, United States.
365. Orsello C, Dommermuth R. Maximizing neonatal early onset group B streptococcal disease prevention with universal culture screening at 35 to 37 weeks gestation: A comparison of GBS detection rates between LIM broth and CNA culture media. Family Medicine **2003**; 35(6): 411-3.
366. Diaz TM, Nieves BM. Comparison between culture media and procedures to detect Streptococcus agalactiae in pregnant women. Available at: http://www.scielo.cl/pdf/rci/v25n2/art03.pdf

http://ovidsp.ovid.com/ovidweb.cgi?T=JS&PAGE=reference&D=emed8&NEWS=N&AN=18483641. Accessed (Diaz) Universidad de los Andes, Facultad de Farmacia Y Bioanalisis, Departamento de Bioanalisis Clinico, Merida, Venezuela.
367. Thinkhamrop J, Limpongsanurak S, Festin MR, et al. Infections in international pregnancy study: performance of the optical immunoassay test for detection of group B streptococcus. Journal of clinical microbiology **2003**; 41(11): 5288-90.
368. Altaie SS, Dryja D. Detection of group B Streptococcus. Comparison of solid and liquid culture media with and without selective antibiotics. Diagnostic microbiology and infectious disease **1994**; 18(3): 141-4.
369. Nguyen TM, Gauthier DW, Myles TD, Nuwayhid BS, Viana MAG, Schreckenberger PC. Detection of group B streptococcus: Comparison of an optical immunoassay with direct plating and broth-enhanced culture methods. Available at: http://ovidsp.ovid.com/ovidweb.cgi?T=JS&PAGE=reference&D=emed4&NEWS=N&AN=1998282943. Accessed (Nguyen, Gauthier, Myles, Nuwayhid, Viana, Schreckenberger) Div. of Maternal-Fetal Medicine, Dept. of Obstetrics and Gynecology, University of Illinois, 820 S. Wood St., Chicago, IL 60612-7313, United States.
370. Baker CJ, Goroff DK, Alpert SL. Comparison of bacteriological methods for the isolation of group of B Streptococcus from vaginal cultures. Available at: http://ovidsp.ovid.com/ovidweb.cgi?T=JS&PAGE=reference&D=emcl2&NEWS=N&AN=0977177520. Accessed (Baker, Goroff, Alpert) Channing Lab., Dept. Med., Boston City Hosp., Boston, Mass. United States.
371. Mason EO, Jr., Wong P, Barrett FF. Evaluation of four methods for detection of group B streptococcal colonization. Journal of clinical microbiology **1976**; 4(5): 429-31.
